# Polymer Adsorbents vs. Functionalized Oxides and Carbons: Particulate Morphology and Textural and SurfaceCharacteristics

**DOI:** 10.3390/polym13081249

**Published:** 2021-04-12

**Authors:** Volodymyr M. Gun’ko

**Affiliations:** Chuiko Institute of Surface Chemistry, 17 General Naumov Street, 03164 Kyiv, Ukraine; vlad_gunko@ukr.net; Tel.: +38-04-4422-9627

**Keywords:** porous polymers, silica adsorbents, activated carbons, functionalized adsorbents, textural and morphological characteristics, adsorbent characterization methods

## Abstract

Various methods for morphological, textural, and structural characterization of polymeric, carbon, and oxide adsorbents have been developed and well described. However, there are ways to improve the quantitative information extraction from experimental data for describing complex sorbents and polymer fillers. This could be based not only on probe adsorption and electron microscopies (TEM, SEM) but also on small-angle X-ray scattering (SAXS), cryoporometry, relaxometry, thermoporometry, quasi-elastic light scattering, Raman and infrared spectroscopies, and other methods. To effectively extract information on complex materials, it is important to use appropriate methods to treat the data with adequate physicomathematical models that accurately describe the dependences of these data on pressure, concentration, temperature, and other parameters, and effective computational programs. It is shown that maximum accurate characterization of complex materials is possible if several complemented methods are used in parallel, e.g., adsorption and SAXS with self-consistent regularization procedures (giving pore size (PSD), pore wall thickness (PWTD) or chord length (CLD), and particle size (PaSD) distribution functions, the specific surface area of open and closed pores, etc.), TEM/SEM images with quantitative treatments (giving the PaSD, PSD, and PWTD functions), as well as cryo- and thermoporometry, relaxometry, X-ray diffraction, infrared and Raman spectroscopies (giving information on the behavior of the materials under different conditions).

## 1. Introduction

The textural characteristics of unmodified and functionalized solid adsorbents and polymer fillers, such as metal and metalloid oxides (synthetic silica, alumina, titania, complex materials, natural zeolites, clays, etc.) and carbons (chars, activated carbons (AC), carbon blacks, carbon nanotubes (CNT), carbon nanofibers (CNF), carbon nanocomposites (CNC), graphene, graphene oxides (GO), etc.), are typically better-studied (as relatively rigid solids with stable characteristics) than those of polymer adsorbents [1,2,3,4,5,6,7,8,9,10,11,12,13,14,15,16,17,18,19,20,21,22,23,24,25]. The latter may be characterized by lower stability of the textural characteristics than stable and rigid solids due to various effects of dispersion media, swelling, aging, freezing with liquids, heating, mechanical loading, as well as due to high fractality, strongly tortuous pores, and disordered texture of nonrigid polymers [26,27,28,29,30,31,32,33,34]. Various composites [35,36,37,38], nanostructured polymers [39,40,41,42,43,44,45], polymer/oxides [46,47,48], and polymer/carbons [49,50,51,52] composites are of interest from a practical point of view. However, accurate textural characterization of polymeric composites, complex filler–polymer and filler–polymer–polymer systems is a more difficult task than a similar challenge for individual oxide, carbon, or polymeric materials. To solve the characterization tasks for complex and hybrid materials, several experimental methods should be used in parallel. Among the textural characterization methods, the adsorption of probes (nitrogen, argon, carbon dioxide, or benzene) is most frequently used [1,2,3,4,5,6]; however, for composites and hybrids, some problems arise. Therefore, additional methods, such as small-angle X-ray or neutrons scattering (SAXS, SANS) [53,54,55,56,57,58], X-ray diffraction (XRD) [59,60,61], high-resolution transmission (HRTEM) and scanning (SEM) electron microscopies [59,62], nuclear magnetic resonance (NMR) spectroscopy with cryoporometry and relaxometry [15,63,64,65], differential scanning calorimetry (DSC) and thermoporometry [66], infrared spectroscopy (FTIR), thermogravimetry (TG) and thermoporometry, dielectric relaxation spectroscopy (DRS), thermally stimulated depolarization current (TSDC) and relaxometry, theoretical simulations, etc. [67,68,69,70,71,72,73,74,75,76,77,78,79,80,81,82,83,84,85,86] should be used. Besides the textural characteristics, the data of these methods allow one to obtain information on the materials’ behavior under different conditions, which could model real situations related to practical applications. However, even accurate experimental data cannot guarantee accurate characterization of the materials because the data could be indirect and, therefore, need additional and typically complex computational treatments. In other words, certain appropriate physicomathematical tools should be applied to obtain anticipated accurate characteristics of complex materials. Typically, the treatment tools include model equations describing various experimental dependences, such as adsorption vs. pressure (gaseous phase) or concentration (liquid phase), scattering intensity vs. scattering angle, freezing/melting or heat flow vs. temperature, etc. To analyze these dependencies (typically complex for complex materials), certain model equations should be used and treated with the applied mathematics methods (used, e.g., to solve integral and differential equations, to minimize functionals, to regularize experimental noise effects, etc.) and related computer programs [3,4,5,6,7,8,31,32,53,54,55,56,63,64,74,75,76,77,78,79,80,81,82,83,84,85,86,87]. Even if the experimental methods give direct information (e.g., TEM, SEM) that certain computer methods and programs should be used to obtain quantitative characteristics, e.g., the particulate morphology, porosity, particle and crystallite size distributions, etc. There are additional problems with the correspondence of the models to complex materials, experimental methods, data, and conditions. For example, using inappropriate models (e.g., the BJH adsorption model developed for mesoporous materials for computation of the PSD for nanoporous materials) or unreasonable ignoration of the top possibilities of a method used (e.g., obtained SAXS data are not treated for comprehensive textural characterization) can result in an incomplete or incorrect picture on the textural characteristics [28,54,87,88]. Clearly, the tasks mentioned above become more complex for nonuniform and multicomponent systems. Note that firm software used with various adsorption analyzers is oriented on such simple materials as silica or carbon, i.e., the treatment results with this software are rather incorrect for complex and hybrid composites. Therefore, to increase the reliability and validity of the characterization of complex systems, several experimental and theoretical methods should be used in parallel with software, including models for complex materials [28,32,54,89,90,91], and this aspect is analyzed here in detail.

There are several adsorbent classes with different textural features. Therefore, it is difficult to accurately describe their various blends using firm software [87,89,90]. There are (i) synthetic highly disperse oxides (fumed silica, alumina, titania, binary and ternary fumed oxides) composed of spherical-like nonporous nanoparticles (NPNP); (ii) natural nanostructured oxides (clays, zeolites) with a complex shape of pores; (iii) porous oxides (silica gels, ordered mesoporous silicas, complex oxides) mainly with cylindrical pores, but with certain deviations and surface roughness; (iv) carbons (chars, activated carbons, graphene, graphene oxide, exfoliated graphite, nanotubes, carbon blacks, fullerites), which can have not only slit-shaped pores but also spherical, cylindrical, wedge-shaped and other pores; (v) polymers (1D, 2D, 3D hydrophobic and hydrophilic, functionalized, synthetic and natural) with complicated and tortuous pore networks; (vi) metal–organic framework structures with complex pore shapes; (vii) complex and hybrid systems with components of different kinds or classes [1,2,3,4,5,6,7,8,9,10,11,12,28,32]. Composites and hybrids can have pores with a shape different from that characteristic for individual components because of the possible penetration of one component into pores of other components. Therefore, the additive models could not appropriately work for composites.

The main characteristics of adsorbents or polymer fillers are linked to their specific surface area (rather accessible than total), porosity (pore volume, pore size and pore wall thickness distributions), adsorption surface sites (types and content), hydrophobicity/hydrophilicity, swelling, freezing of pore-confined liquids (especially water), conformation stability in different media vs. temperature, effects of dispersion media, adsorbates, co-adsorbates, etc. These characteristics could be changed during aging, mixing, wetting, drying, mechanical loading, etc. Additionally, these changes could be different for different components of the composites. Despite only a certain part of the characteristics of adsorbents or fillers can play a crucial role on the practical applications, the accurate determination of these characteristics remains a complex task.

In this work, some aspects of relatively comprehensive characterization of individual and complex, unmodified and functionalized materials with oxides, carbons, and polymers are analyzed to show the possibilities of parallel applications of several experimental methods with the treatment of the data with simple regularization/functional minimization or more complex self-consistent regularization procedures [87,89,90,91,92] and other applied mathematics and computational methods. Note that some experimental methods, which complement each other well, can give different pictures on the same materials. For example, nitrogen (or Ar, CO_2_, C_6_H_6_) adsorption and SAXS can give significantly discriminate information (specific surface area, *S*, pore size distributions, PSD, pore wall thickness distributions, PWTD, particle size distributions, PaSD, etc.) on accessible (open) and both accessible/inaccessible (open/closed) pores, respectively [54,55,56,93]. Additionally, the degree of pore accessibility depends on an adsorbed probe’s molecular size; therefore, different probes can give different information [3,4,5,6,94,95,96]. However, accurate and intelligent comparison of all the data allows one to obtain much more information on the materials than in the case of using only one method. Note that it is important to use the models accurately describing each component of the materials studied. For example, for highly disperse silicas composed of NPNP, a model of voids between spherical particles in random aggregates is better than a model of cylindrical pores in silica. For composites, various models, including different pore shapes for different components, e.g., for carbon/nanooxides, polymer/nanooxides, polymer/carbons, etc. (described here), are much better than any simple model (from firm software) for any individual material as a component of composites because it poorly describes other components with different textures [87,89,90]. Unfortunately, the simplified approach for textural characterization of composites based on the firm adsorption methods with firm software is typically used.

The effects of temperature, media, swelling, freezing/melting, confined space effects, etc., which may affect the textural characteristics, could be analyzed using a set of methods, such as NMR cryoporometry and relaxometry, DCS thermoporometry, TSDC relaxometry, TG thermoporometry, probe adsorption from liquid and gaseous phases, electron microscopies, etc. The application of some additional methods in parallel to the adsorption, SAXS, and TEM/SEM can give relatively comprehensive information on complex materials being under different conditions. Additionally, this allows one to forecast changes in the characteristics, temperature and interfacial behaviors of the complex materials under different conditions that are of importance from a practical point of view. Thus, this work aims to show certain aspects of relatively comprehensive characterization of complex materials of different types affected by dispersion media, temperature, pressure, aging time, and other conditions using a set of experimental and theoretical methods. In these approaches, the differences in the component characteristics are considered at a model level and considered upon using the self-consistent regularization procedures giving information on the contributions of the components into total characteristics, such as the PSD and PaSD functions. Some additional details on the materials analyzed here and used methods are given in Supplementary Materials file.

## 2. Materials and Methods

### 2.1. Materials

In this comparative study, various unmodified, modified and complex materials are used. First, highly disperse fumed metal and metalloid oxides (FMO with silica, alumina, titania, and related binary and ternary oxides, Pilot plant of Chuiko Institute of Surface Chemistry (CISC), Kalush, Ukraine) and Cab–O–Sil HS5 (Cabot Corp., Boston, USA) are characterized by certain distributions of NPNP and the specific surface area *S*_BET_ in the range of 30–500 m^2^/g, bulk density ρ_b_ = 0.04–0.15 g/cm^3^, pore volume *V*_p_ = 0.1–1.5 cm^3^/g, and large empty volume in the powders *V*_em_ = 4–25 cm^3^/g. Second, porous silica gels Si-40, Si-60, Si-100 (Merck); HP39 and Gasil 200DF (Crosfield Ltd., Warrington, UK), ordered mesoporous silicas MCM-41, MCM-48, and SBA-15 (CISC), and precipitated silica Sipernat 50 (Evonik) represent spherical-like particles in the millimeter range at *S*_BET_ = 300–1200 m^2^/g. Third, highly porous chars and activated carbons (AC) (MAST Carbon Technology Ltd., UK; Hypersil, Astmoor, UK; Wood Dry Distillation Works and HPSD, Hajnówka, and PSO MASKPOL, Poland, CISC) at S_BET_ = 500–3500 m^2^/g and *V*_p_ = 0.5–3.0 cm^3^/g. Four, polymers, such as natural (starch, etc.) and synthetic (poly(vinyl pyrrolidone (PVP), poly(vinyl alcohol) (PVA), polydimethylsiloxane (PDMS), polymethylsiloxane (PMS), polystyrene, copolymers of styrene and divinylbenzene, acrylic ester polymer, a copolymer of 1,4-phenylene dimethacrylate and divinylbenzene, and others were studied under different conditions. Additionally, complex and hybrid systems with PVA/AC, fumed silica/PDMS, etc., are described here mainly concerning the morphological and textural characteristics. All these materials can be considered as representatives of various classes of simple and complex adsorbents and polymer fillers described in detail elsewhere [4,5,6,7,8,9,10,11,12,28,31,32,54,55,56,74,75,76,77,78,79,80,81,82,83,84,85,86,87,89,90,91]. Some details on the materials used here are given in the Supplementary Materials file.

### 2.2. Methods

The adsorption of probe (nitrogen, benzene) compounds was used to evaluate the accessible surface area, pore volume, and pore and particle size distributions. The nitrogen adsorption–desorption isotherms (Micromeritics ASAP 2010, 2020, 2405N, or 2420 and Quantachrome Autosorb adsorption analyzers) could be used to compute the pore size distributions (differential PSD *f*_V_(*R*) ~d*V*_p_/d*R* and *f*_S_(*R*) ~d*S*/d*R*) using various approaches [32,87,89,90]. Some simple approaches could include various systematic errors caused by an inappropriate model of pores (e.g., cylindrical pores poorly model voids between NPNP in supra-NPNP structures), inappropriate parameters of solids (e.g., parameters of carbons poorly describe polymeric adsorbents), etc. As a whole, for materials with the complex topology of pores or/and composed of several different phases (e.g., FMO and polymers or carbons, etc.), firm (Micromeritics, Quantachrome, etc.) software can give incorrect results with systematic errors. Better results could be obtained using complex pore models with slit-shaped (S) and cylindrical (C) pores and voids (V) between spherical nanoparticles (SCV method) with the corresponding parameters for different phases [87,89,90]. Additionally, the chemical structure of a solid surface (e.g., hydroxyls or other functionalities) can affect the interactions (and orientation, i.e., effective area of a surface occupied by a molecule) of nitrogen or other probe molecules with a surface that can be studied using quantum chemistry methods (see Supplementary Materials file). The SCV method with a self-consistent regularization (SCR) procedure [87] allows one to consider the presence of several phases since the parameters of several types of surfaces (e.g., silica, alumina, titania, carbon, carbohydrate polymers, etc.) could be simultaneously used with an appropriate pore model for each component. The SCR/SCV procedure gives information on contributions (weight coefficients) of different pore types and different components into the total porosity and specific surface area. As a whole, the model errors can remain upon using the SCV/SCR method because the texture of any adsorbent is not strongly ordered (pores can have very complex shapes) and affected by surface roughness, etc. However, the SCV/SCR method reduces the systematic errors appearing upon applying the firm software for complex materials. Note that the PSD could be calculated using molecular density functional theory (DFT) methods, such as nonlocal DFT (NLDFT), quenched solid DFT (QSDFT), 2D-NLDFT, well-developed modified Nguyen–Do (MND) method or others. For better view of the PSD at large values of *R*, the differential PSD concerning the pore volume *f*_V_(*R*) ~d*V*/d*R*, ∫*f*_V_(*R*)d*R* ~*V*_p_ could be recalculated to incremental PSD (IPSD) at Φ_V_(*R_i_*) = (*f*_V_(*R_i_*_+1_) + *f*_V_(*R_i_*))(*R*_i+1_ − *R*_i_)/2 at ∑Φ_V_(*R_i_*) = *V*_p_. The *f*_V_(*R*) and *f*_S_(*R*) functions could also be used to calculate contributions of nanopores (*V*_nano_ and *S*_nano_ at the radius in the range 0.35 nm < *R* < 1 nm), mesopores (*V*_meso_ and *S*_meso_ at 1 nm < *R* < 25 nm), and macropores (*V*_macro_ and *S*_macro_ at 25 nm < *R* < 100 nm) into the total pore volume and specific surface area. Clear, an incorrect PSD results in incorrect values of the textural characteristics. Some additional information on the adsorption methods is given in Figures S1–S16 and Table S1 in the Supplementary Materials file.

The spectral (NMR (Varian 400 Mercury or Agilent DD2 600 MHz NMR spectrometer, Agilent, Santa Clara), FTIR (FTIR 1725× PerkinElmer (PerkinElmer Inc., Waltham, MA, USA) or Specord M80 (Carl Zeiss, Jena, Germany), and Raman (inVia Reflex, Renishaw, Charfield, UK) spectroscopy) methods were used to analyze the chemical structure of the materials, content and composition of surface functionalities, structure of the adsorption layers at a surface of different materials [28]. Some IR spectra are shown in Figures S17–S20.

The differential PSD functions *f*(*r*) based on the SAXS data (Empyrean diffractometer, PANalytical, Cu K_α_ radiation at λ = 0.15418 nm, 2θ = 0.5–5°) were calculated using Fredholm integral equation of the first kind for scattering intensity *I*(*q*), as well as the total surface area, pore, pore wall and particle size distributions [54,55,56] (see Supplementary Materials file, Figures S21–S25). The main advantage of the SAXS method upon the textural characterization is that all open and closed pores could be analyzed in contrast to the adsorption methods giving the characteristics only of pores accessible for probe molecules. The SAXS patterns could be used to compute the PaSD for spherical, cylindrical, or lamellar particles alone or in any mixture (see Supplementary Materials file). For the latter, the self-consistent regularization procedure allows us to estimate the contributions of particles of different shapes [55,56].

The TEM (TECNAI G2 F30 microscope, FEI–Philips or JEM–2100 F, Tokyo, Japan) and SEM (Quanta^TM^ 3D FEG, FEI, FE–SEM, Hitachi S–4700, Tokyo, Japan, or FEI NovaSEM 230) methods were used to analyze the particulate morphology, particle size distributions, and porosity (see Figures S26–S31). The TEM/SEM PaSD can be calculated using such software as ImageJ (with granulometry plugin) [97], Fiji (with local thickness plugin) [98], and some others [85,86].

For the textural and morphological characterization of various materials, the thermal characteristics NMR, DSC (PYRIS Diamond (PerkinElmer Instruments, Waltham, MA, USA), TSDC (Novocontrol Technologies), and TG (Derivatograph Q-1500 D apparatus, MOM, Budapest, Hungary) with related cryo- and thermoporometry, and relaxometry) and colloidal data (QELS for PaSD) could be effectively used as additional tools [28]. Their use is based on the confined space effects depending on pore sizes and features of the temperature behavior (freezing/melting, adsorption/evaporation, relaxation time, dipolar currents, etc.) of adsorbates located in different pores (see Figures S32–S39).

A certain set of methods could be selected for each type of the materials (being under certain conditions in certain dispersion media) and characteristics to obtain appropriate and quite comprehensive information. Here some aspects of the textural and morphological characterization of various materials are analyzed. This is done concerning the selection of experimental methods, models, and related treatments of the data using certain approaches and methods. They provide a set of the distribution functions of the characteristic parameters, such as PSD, PaSD, and PWTD, with the parallel evaluation of contributions of pores and particles of different shapes for a more comprehensive description of the materials. These approaches include model integral equations solved with the self-consistent regularization procedures. Some details of the used methods (experimental, Sections S1–S9, Figures S1–S39, Table S1, Equations (S1)–(S34), and theoretical, Section S10, Figures S40–S50, Tables S2 and S3) are described in detail in Supplementary Materials file.

## 3. Results and Discussion

There is a complex problem of accurate evaluation of the textural characteristics of composites using adsorption methods [87,89,90]. The estimated textural characteristics depend on a kind of probe adsorbate, pretreatment and measurement conditions, used data treatment methods, as well as on chemical composition, particulate morphology and texture of the materials. Even estimation of the specific surface area (*S*_BET_) (as a simple task in the textural characterization) depends on several factors: (i) a type of probe adsorbate (nitrogen, argon, benzene, carbon dioxide, etc.), (ii) the pressure range applied on calculations, (iii) the surface area (σ_0_) occupied by an adsorbate molecule (e.g., the σ_0_ value for nitrogen adsorbed onto carbons and metal/metalloid oxides differs as 0.162 and 0.137 nm^2^, respectively, due to adsorbed molecule orientation effects), (iv) pore shapes and accessibility, etc. [5,93] (see Figure S1 in Supplementary Materials file).

The tasks of estimation of the pore volume (*V*_p_ and its components *V*_nano_ at radius *R* < 1 nm, *V*_meso_ at 1 nm < *R* < 25 nm, and *V*_macro_ at *R* > 25 nm) and PSD have several aspects. Some adsorbents composed of relatively stable nanoparticles (e.g., nanosilica, Figure S2) demonstrate textural instability of upper-hierarchy structures, such as aggregates of NPNP and agglomerates of aggregates (supra-NPNP structures). Therefore, any treatment (e.g., mechanical loading, mechanochemical activation (MCA), hydrocompaction, etc.) results in significant changes in the supra-NPNP structures concerning the textural characteristics (*V*_p_ and PSD, Figure S3). It is also affected by aging (Figure S4), mixing with polymers (Figure S5), surface modification (Figure S11) in contrast to the PaSD of stable NPNP (Figure S2). Some other adsorbents, e.g., complex nanooxides (Figures S6, S7, and S10), carbons (Figures S8, S9 and S13), porous polymers (S12), can undergo significant textural changes upon activation, modification, washing or wetting–freezing. However, their chemical structure practically does not change (Figures S19 and S20). Note that the analysis of the water adsorption (Figures S14–S16, and appearing in several bands in the IR spectra, Figures S17–S20) needs special approaches (Equations (S9)–(S12)) because water tends to be adsorbed in various clusters [28] in contrast to nitrogen or argon, which weakly sense the surface chemistry [3,93,94]. Thus, the textural and morphological characterization of various materials is not a simple task.

The use of several different parallel methods that consider the nature of the materials (especially complex and hybrid) may improve the reliability of obtained morphological and textural characteristics. For example, such a set of methods as adsorption and SAXS (Figures S21–S25), TEM/SEM with appropriate treatment of images, adsorption and infrared spectroscopy (Figures S26–S31), SAXS and QELS (Figure S32), NMR cryoporometry (Figures S33 and S34), DSC thermoporometry (Figure S35), TSDC relaxometry (Figures S36–S38), confocal laser scanning microscopy (Figure S39), and theoretical modeling (Figures S40–S50) gives much more morphological, textural, and structural information. This may provide a much better and complete characterization of complex materials than the use only of one method (e.g., adsorption or TEM) from this set. As a whole, using the adsorption, microscopic, spectral (FTIR, Raman, XPS, etc.), and structural (XRD, NMR, EDAX, and TPD MS) methods allows one to obtain a practically comprehensive characterization of simple, complex, and hybrid materials.

**Figure 1:**
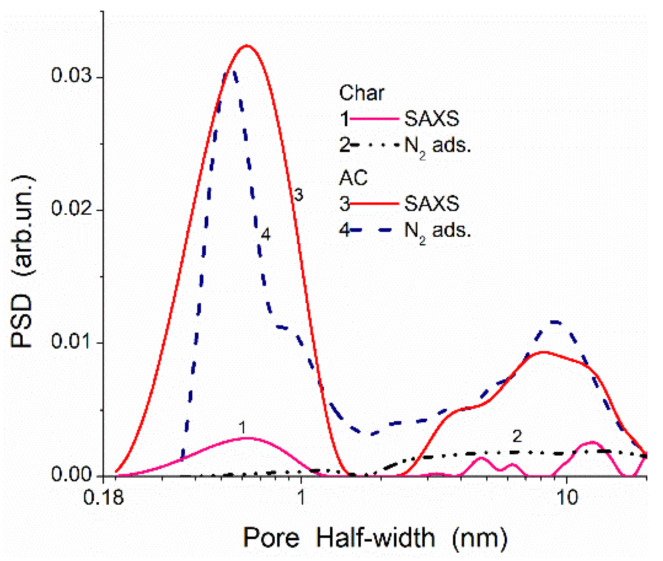
Pore size distributions of a char/bentonite (20/80 *w/w*) composite (*S*_BET_ = 122 m^2^/g and *S*_SAXS_ = 262 m^2^/g), prepared upon carbonization of resorcinol–formaldehyde resin added to bentonite (curves 1 and 2), and activated carbons (AC) (carbonization of phenol–formaldehyde resin and subsequent activation by CO_2_ at 1183 K with 60% burn-off) (*S*_BET_ = 1999 m^2^/g and *S*_SAXS_ = 2211 m^2^/g, curves 3 and 4) calculated using SAXS (curves 1 and 3) and nitrogen adsorption–desorption isotherms with a model of slit-shaped and cylindrical pores and voids between nanoparticles with self-consistent regularization procedure (SCV/SCR) (curves 2 and 4) methods.

**Figure 2:**
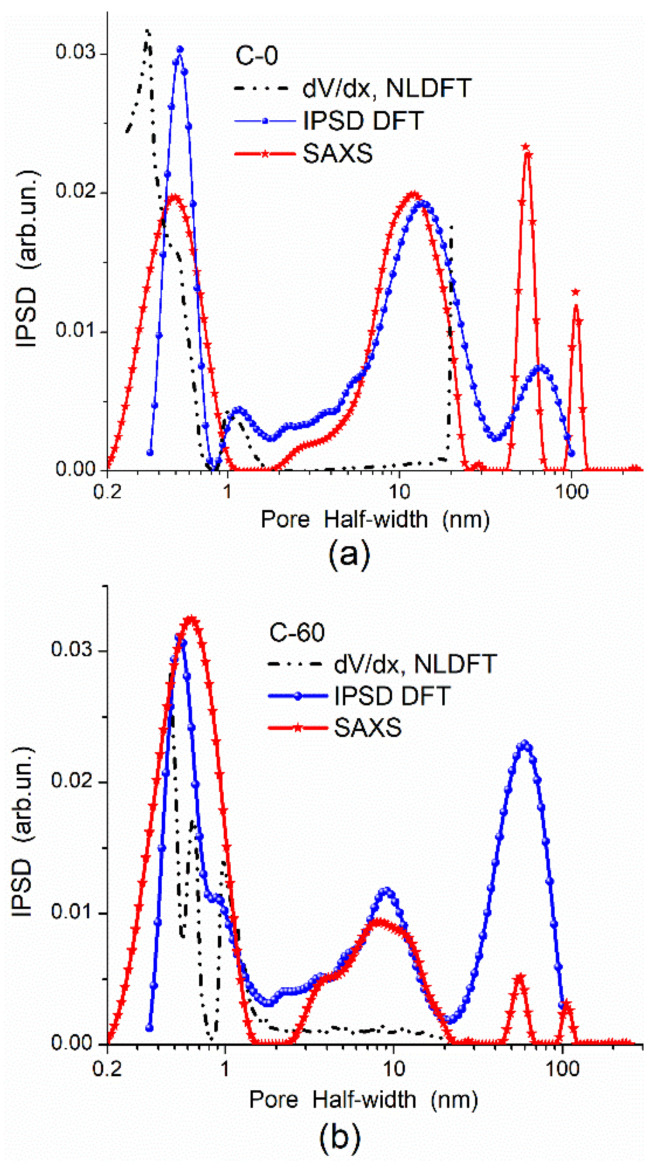
Comparison of pore sizes (PSDs) calculated from the N_2_ adsorption and SAXS data for samples (**a**) C−0 (phenol–formaldehyde char, *S*_BET_ = 549 m^2^/g, char with zero burn-off) and (**b**) C-60 (AC with 60% burn-off, *S*_BET_ = 1999 m^2^/g).

**Figure 3:**
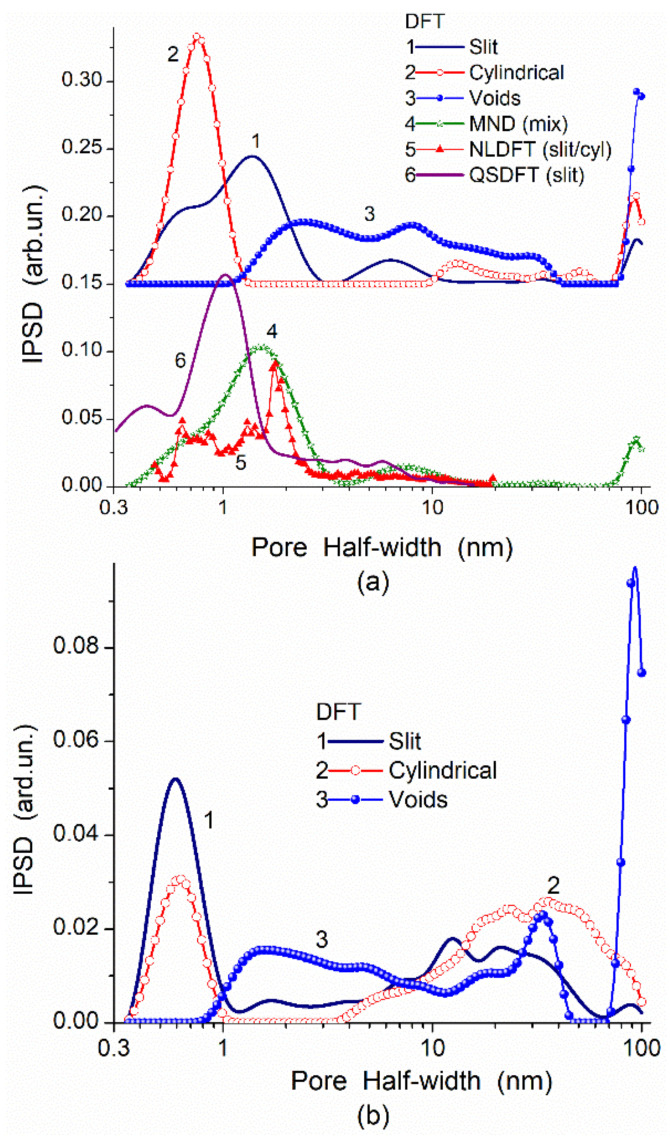
Comparison of incremental PSDs (IPSDs) for (**a**) C-86 (86% burn-off, *S*_BET_ = 3461 m^2^/g, pore volume *V*_p_ = 2.32 cm^3^/g) and (**b**) C-30 (30% burn-off, *S*_BET_ = 1145 m^2^/g, *V*_p_ = 1.19 cm^3^/g) computed from the nitrogen adsorption isotherms using different models of pores (slit-shaped and cylindrical pores, voids between spherical particles, mixtures with slit/cylindrical pores or slit/cylindrical/voids) calculated with molecular density functional theory (DFT), modified Nguyen–Do (MND), nonlocal DFT (NLDFT), and quenched solid DFT (QSDFT) methods.

**Figure 4:**
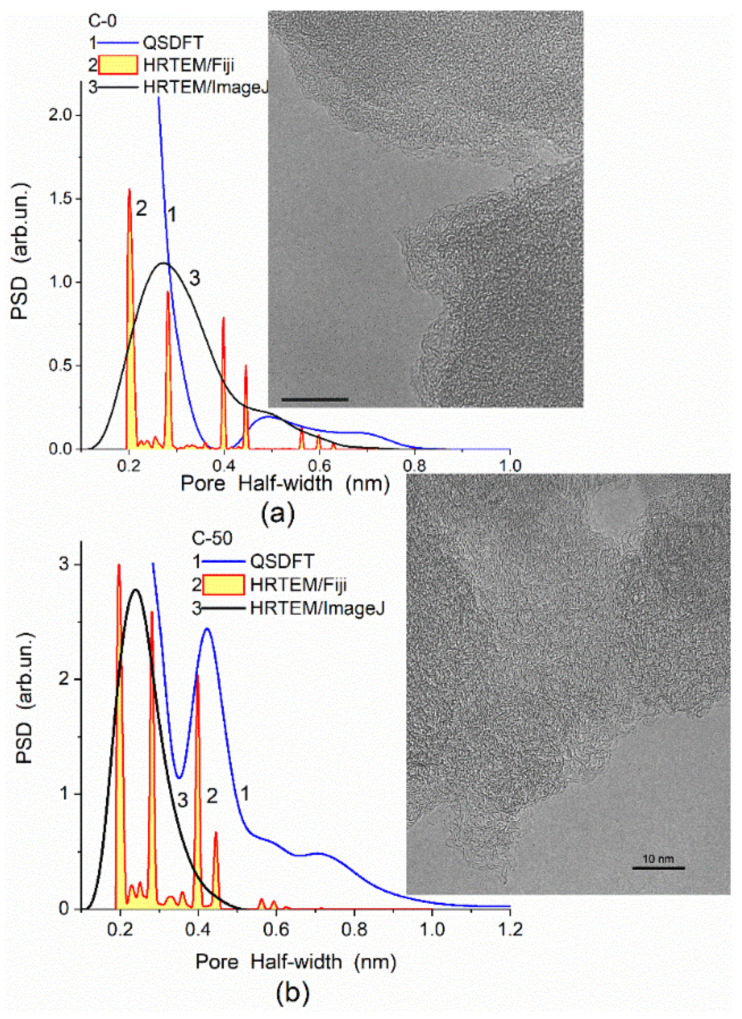
PSD calculated using the QSDFT method with slit-shaped pore model for (**a**) char (porous phenol–formaldehyde resin beads were heated in a CO_2_ flow to 1073 K at a ramp rate of 3 K/min, *S*_BET_ = 590 m^2^/g) and (**b**) AC with 50% burn-off (*S*_BET_ = 1664 m^2^/g); inserts show HRTEM images of these carbons (scale bar 10 nm) used to obtain the PSD with Fiji (local thickness plugin) and ImageJ (granulometry plugin) software.

**Figure 5:**
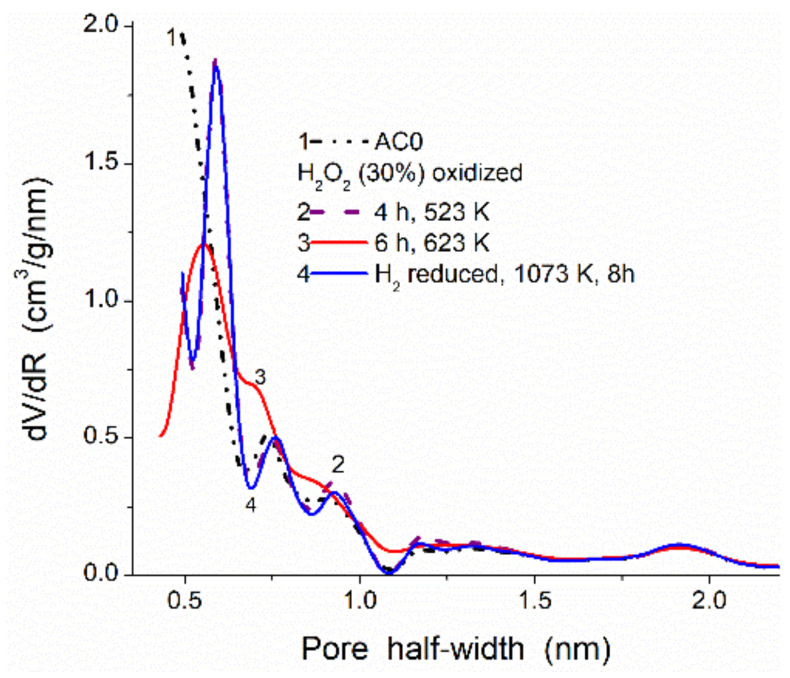
NLDFT PSD for AC initial (prepared from plum stones [99]), oxidized by H_2_O_2_ for 4 and 6 h, and reduced by H_2_ at 1073 K for 8 h (*S*_BET_ = 1054, 1149, 1162, 1201 m^2^/g, *V*_p_ = 0.720, 0.723, 0.713, 0.733 cm^3^/g, respectively).

**Figure 6:**
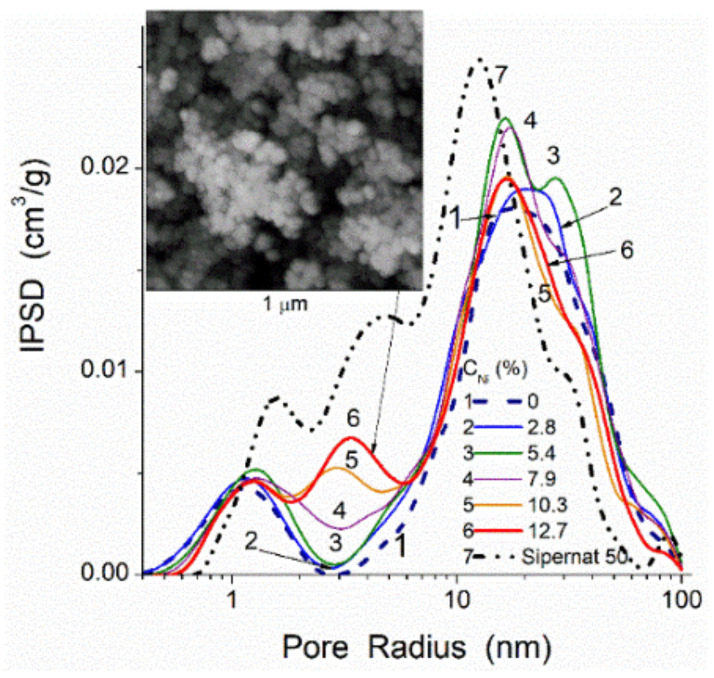
SCV/SCR PSD of carbon/silica/Ni composites formed upon carbonization (500 °C, nitrogen atmosphere, 3 h) of potato starch with addition of Ni(NO_3_)_2_ (0.5 to 2.5 mmol per 3 g of silica Sipernat 50 (503 m^2^/g, 1.29 cm^3^/g) and 27 g of starch): resulting final Ni content from 2.8 to 12.7 wt % in composites at *S*_BET_ = 292, 326, 330, 322, 322, 320 m^2^/g; *V*_p_ = 0.89, 0.98, 1.07, 1.05, 0.98, and 1.00 cm^3^/g, respectively.

**Figure 7:**
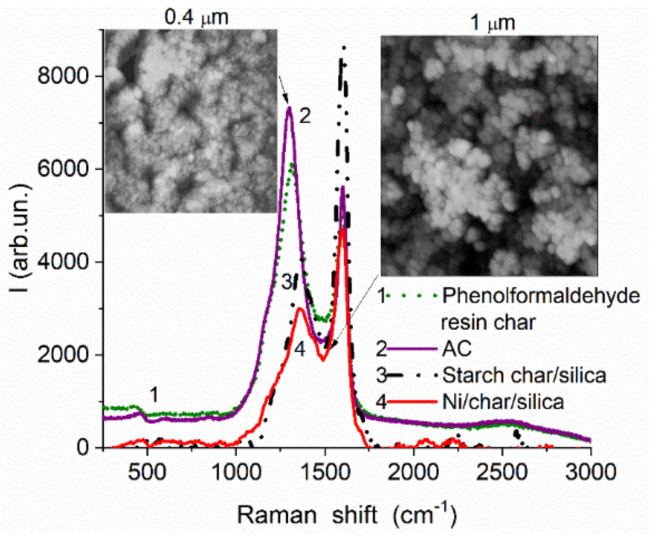
Raman spectra (inVia Reflex, Renishaw, UK) of materials containing carbons of different origin: phenol–formaldehyde resin char (curve 1, *S*_BET_ = 534 m^2^/g, *V*_p_ = 0.9 cm^3^/g) and related AC with 65% burn-off (curve 2, S_BET_ = 2019 m^2^/g, *V*_p_ = 1.86 cm^3^/g); char formed using starch without (curve 3, 292 m^2^/g, 0.89 cm^3^/g) and with addition of Ni(NO_3_)_2_ (curve 4, 12.7 wt % Ni in composite, 320 m^2^/g, 1.0 cm^3^/g) at Sipernat 50 surface.

Certain features inherent in each method should be considered upon the parallel use of a set of different methods. For example, there is a significant difference in the material characterization based on the adsorption and SAXS methods. The former gives information on accessible (open) surface/pores, but the latter gives information on both open and closed pores. This difference is especially large for carbon adsorbents with a small degree of activation (i.e., at a large contribution of closed pores), e.g., for the pore size distributions (Figure 1, comp. char with zero burn-off and AC with various burn-off activation, and Figure 2, Figure 3, Figure 4, Figure 5, Figure 6 and Figure 7, S13 and S22). The difference in the results based on the SAXS and adsorption data strongly decreases with increasing activation degree (contribution of closed pores decreases at a high burn-off degree) [54]. Typically, the specific surface area evaluated from the SAXS data (*S*_SAXS_) is larger than the *S*_BET_ value estimated from the adsorption data. The *S* difference (as well as PSD) decreases with increasing activation degree. For example, for char/bentonite, *S*_BET_ = 122 m^2^/g and *S*_SAXS_ = 262 m^2^/g (i.e., only 46.6% of the total surface area is accessible for the nitrogen molecules), but for AC at 60% burn-off, 90.4% of the surface is accessible for the nitrogen molecules (Figure 1).

In the adsorption methods, the adsorption–desorption isotherms of various probes can give different textural characteristics because the accessibility of a surface in narrow pores depends strongly on the molecular sizes of probe adsorbates. To obtain accurate textural (or morphological) characteristics, the adsorption data should be treated using molecular density functional theory (DFT) methods (such as nonlocal DFT (NLDFT), quenched solid DFT (QSDFT), 2D-NLDFT) or other well-developed methods. For example, a DFT method developed [87,89] gives better PSD than NLDFT since the DFT PSD correspond to the SAXS PSD in a broad range of pore sizes in contrast to the NLDFT PSD (Figure 2) or QSDFT and modified Nguyen–Do (MND) PSD [7,28,32,89,90] (Figure 3). However, in the range of narrow nanopores (<0.5 nm in half-width), the QSDFT method gives better PSD than NLDFT, DFT, or MND PSD compared to the PSD HRTEM computed with ImageJ (granulometry plugin) [97] or Fiji (local thickness plugin) [98] (Figure 4). Thus, the accuracy of the textural analyses depends strongly on a correct choice of experimental methods, conditions, and data treatment methods [89,90].

**Figure 8:**
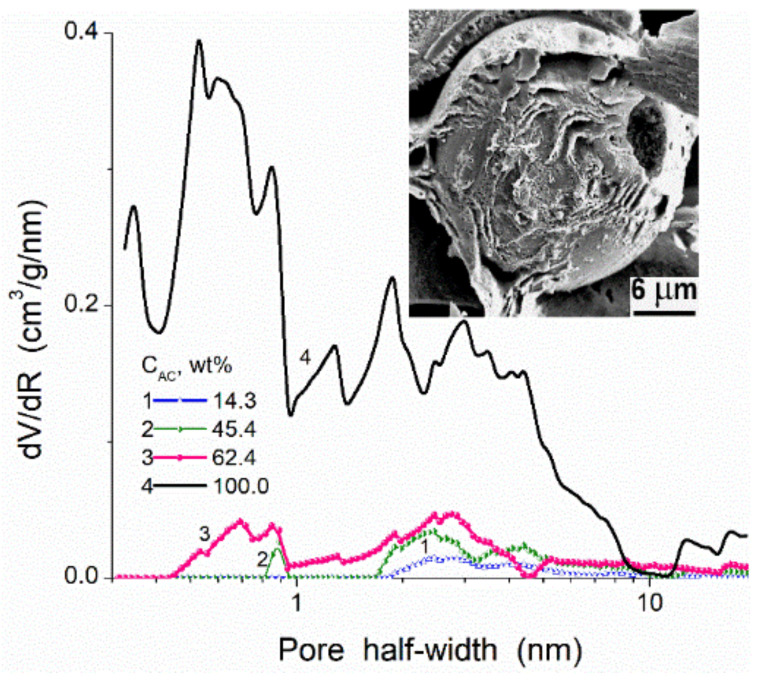
Pore size distributions (NLDFT, model of slit/cylindrical pores in carbons) for poly(vinyl alcohol) (PVA) cryogels (glutaraldehyde as a crosslinker) filled by activated carbon microparticles (*S*_BET_ = 979 m^2^/g, *V*_p_ = 1.33 cm^3^/g) at different amounts (curves 1–3) and initial AC alone (curve 4); insert: SEM image of AC particle covered by PVA.

Despite carbons (chars, AC, carbon black, graphite, carbon composites, etc.) are chemically stable materials, they can be activated, oxidized, reduced, functionalized, etc. (Figure 5, Figure 6, Figure 7 and Figure 8 and S13) under certain conditions. These processes result in changes not only in the surface chemistry of the carbon materials but also in their textural and morphological characteristics. Under relatively soft activation conditions in the controlled atmosphere, the textural changes of the carbon adsorbents could not be very great (Figure 5 and Figure S13). However, chemical modification of a surface (e.g., appearing or removal of polar O-containing surface functionalities) can affect the σ_0_ value for adsorbed nitrogen molecules. Therefore, for comparison of texturally similar materials, but having different surface functionalities (e.g., O- and H-containing groups), it will better to use the adsorption of argon instead of nitrogen [93] or to use a set of additional characterization methods (e.g., SAXS. TEM, etc.).

For hybrid adsorbents or polymer fillers, e.g., carbon deposited onto nanooxides (composed of NPNP) or porous oxides, the morphological and textural characteristics of final composites depend on many factors. First, the morphology, texture, chemical structure of a matrix and other components (e.g., carbon precursors, atmosphere composition) play an important role. Second, carbon precursor structure and reactivity and reaction products, as well as the formation of phases catalytically active in carbonization, e.g., metals, metal oxides (Figure 6) or effectively interacting with a matrix (e.g., hydrothermal treatment in formed water vapor due to the presence of O-, H-, OH-containing groups in the precursors) are additional and important factors. Third, reaction conditions (temperature, pressure, flow rate) and post-reaction treatments can affect the morphology and texture of the final composites. These conditions can also affect contributions of different kinds of carbons characterized by various contributions of disordered/ordered structures, which can be analyzed using Raman spectroscopy. Structures with sp^3^ C (D band in the Raman spectra at lower Raman shifts, Figure 7) and more ordered structures with sp^2^ C (G band at higher Raman shifts) are well distinguished in the Raman spectra. Typically, the D band is broader (due to a variety in disordered structures) than the G band, and their shapes depend on many factors. Note that for mechanically treated blends of more (silicas) and less (carbons) rigid components, it is the possible deposition of fragments of soft components onto the outer surface or into the pores of rigid particles. The analysis of these effects is possible within the scope of SCV/SCR using models of various shapes of pores in various materials [87,90]. The use of any firm software does not give correct results. Thus, the morphological, textural, and chemical characteristics of carbons and related composites determine their properties as adsorbents, fillers, catalysts, etc. Therefore, accurate evaluation of the characteristics of the composites is of importance to forecast the behavior of the materials upon their practical applications under different conditions, e.g., upon interactions with polymers.

The properties and characteristics of all components alone and used on preparation, e.g., polymer/filler composites, allow one to evaluate possible consequences concerning possible changes in the characteristics of the final materials. For example, one can assume that using highly porous AC (microparticles) as a filler of macroporous poly(vinyl alcohol), PVA cryogels can result in blocking of nano/mesopores of AC by PVA. Really, in these composites, a great carbon content, even at 62.4 wt % of AC in the AC/PVA composite [100], does not provide high nano/mesoporosity of the composite (Figure 8). The use of these composites in aqueous or other liquid media can sightly improve the accessibility of pores due to swelling effects. However, this improvement could be small due to penetration of linear polymer (PVA) or glutaraldehyde (used as a crosslinker) oligomers into nano/mesopores of AC and strongly blocking of them. Thus, there is no sense in using highly porous AC fillers of polymers. The use of carbon blacks as fillers of polymers could be better than AC since their surface chemistry, PaSD, mechanical and thermal properties are close, but carbon black particles are practically nonporous in contrast to AC.

For nanostructured fillers, such as fumed silica or other fumed oxides, there is a problem of a correct choice of the filler content because filler NPNP, having high external surface area, can very effectively interact with polymers. This interaction depends on pretreatment of the blends, polymer–polymer and polymer–NPNP interactions, and the dispersion media. The hydroxyl groups play an important role in the properties not only of nanooxides [1,2] but also of other nanostructured materials based on the siloxane bridges ≡Si–O–Si≡, ≡Si–OH, and functionalized groups. For example, commercial polymethylsiloxane (PMS) hydrogel, synthesized using methyltrichlorosilane as a precursor, contains ~7–8 wt % of PMS and 93–92 wt % of water (Enterosgel, Kreoma-Pharm, Ukraine) [101]. It is characterized by incomplete crosslinking of residual silanols; therefore, it is hydrophilic and contains adsorbed water (see Figure S20). After drying at room temperature for a week, the amount of water bound in PMS is small (0.7 wt %). Dried and stirred PMS rehydrated (*h* = 1 g/g) and stirred again is hydrophilic, but to a smaller degree than the initial hydrogel due to partial crosslinking of residual silanols. The bulk density of hydrocompacted wetted PMS powder is ρ_b_ ≈ 0.5 g/cm^3^ at *V*_em_ ≈ 1.5 cm^3^/g, i.e., it remains as a disperse material, as well the blends with nanosilica A-300 [101]. Air-dried PMS and dry nanosilica A-300 (Pilot plant of Chuiko Institute of Surface Chemistry, Kalush, Ukraine) mixed in a porcelain mortar, and then with distilled water (*h* = 1 g/g) give a hydrophilic system. If the system is stirred without any strong mechanical loading (simple mixing), that ρ_b_ ≈ 0.5 g/cm^3^ (PMS/A-300). If the system is stirred under strong mechanical loading (careful grinding in a porcelain mortar with strong hand-loading giving ~20 atm, estimated from the mortar’s geometry and a pestle used and a loading weight, for 15 min) that ρ_b_ ≈ 0.6 g/cm^3^. This is a hydrocompacted sample (cPMS/A-300). Hydrocompaction effects for various nanomaterials depend on their structure, water amounts, mechanical loading, and treatment time [28,32,101].

**Figure 9:**
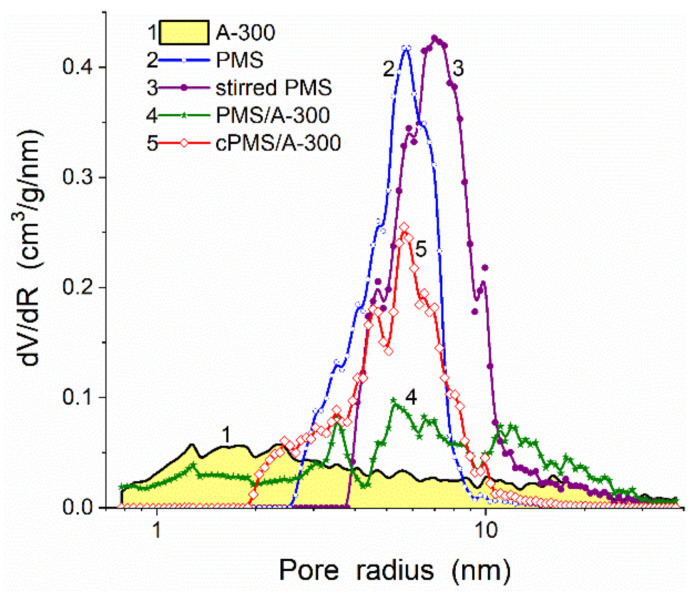
Differential (*dV*/*dR*) NLDFT PSD for A-300 (curve 1), dried polymethylsiloxane (PMS) hydrogel (2), dried, rehydrated, hydrocompacted, and dried PMS (3), stirred wetted PMS/A-300 without (curve 4) and with (curve 5).

If a polymer has a middle 2D–3D structure, e.g., PMS with CH_3_ group at each Si atom, which is linked with neighboring Si atoms by the ≡Si–O–Si≡ bridges (see additional information on PMS in Supplementary Materials file), that the PSD for its blends with nanosilica A-300 can be similar to that of initial PMS or A-300 depending on the blend pretreatment conditions (Figure 9 and Table 1). As a whole, linear polymers with relatively weak polymer–polymer interactions are well distributed in the blends with nanooxides even at low content of solvents (30–50% of water or water/alcohol) [6,7,8,9,28,32,75,77,79,84]. However, at comparative interaction energies for polymer–polymer and polymer–NPNP (e.g., PVA/A-300) [32], the uniform distribution of NPNP in the polymer matrix becomes difficult similar to the systems with chemical bonding of polymers to surface functionalities at NPNP of nanooxides [77].

**Figure 10:**
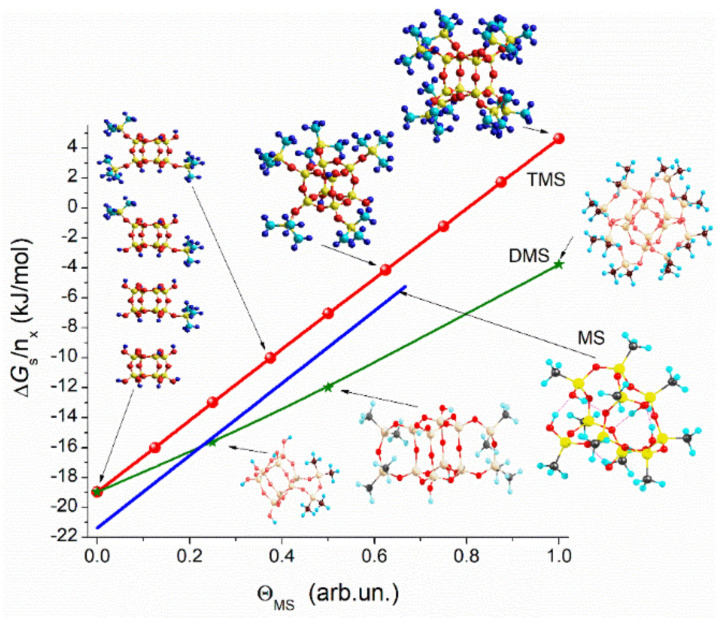
Gibbs free energy of solvation (SMD/ωB97X-D/cc-pVDZ [102]) divided by a number of hydroxyls in the initial clusters vs. the degree of substitution of the hydroxyls by the trimethylsilyl (TMS), dimethylsilyl (DMS) and methyl groups.

**Figure 11:**
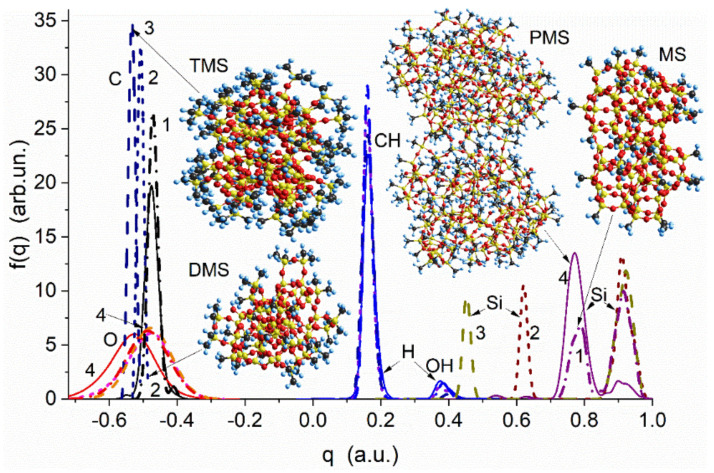
Charge distribution functions of the H, C, O, and Si atoms in nanoparticles of silica with methyl silyl (MS, dot-dashed lines, curves 1), dimethylsilyl (DMS, short-dashed lines, curves 2), and trimethylsilyl (TMS, dashed lines, curves 3) groups and two PMS nanoparticles (solid lines, curves 4) (PM7 [107,108] method).

Quantum chemical calculations of functionalized nanoparticles (using the Gaussian program suit with solvation SMD method [102]) show that despite their different hydrophilicity (Figure 10), the charge distribution functions (CDF) for the H atoms in the ≡Si–CH_3_ groups (Figure 11) are practically the same for four kinds of materials, such as modified silicas with methyl silyl (MS), dimethylsilyl (DMS), and trimethylsilyl (TMS) groups and PMS nanoparticles. The difference in the hydrophobic characteristics of these materials can be explained by the difference in the number of the ≡Si–CH_3_ groups per square unit of a surface, surface topology, and presence of residual silanols. The particle and supra-NPNP geometry can affect the hydrophobicity. Therefore, Δ*G*_s_/*n*_x_ vs. the degree of hydroxyl substitution (Θ_MS_) shows greater hydrophobicity of MS-silica than DMS–silica (Figure 10). The presence of certain structural hierarchies from nano- to micro- and macro-scales of functionalized silicas (alone or in composites with polymers) with the same functional groups can provide superhydrophobicity at contact angles for water drops greater than 150° [103,104,105,106] that is impossible for any uniform functionalized surface.

The sizes of the theoretical models used here (see Supplementary Materials file) correspond to real sizes of nanoparticles (Figures S40–S42) using the DFT or ab initio [102] and semiempirical (e.g., PM7 and PM6 [107,108]) methods. This allows one to accurately model the interfacial layers for various adsorbates (Tables S2 and S3), showing, e.g., variations in the orientation of the adsorbed molecules concerning a surface plane. The latter explains the diminution of the area occupied by a nitrogen molecule at a surface with hydroxyl groups, as well as the formation of water clusters, which can be attributed to strongly (changes in Gibbs free energy Δ*G* < −0.5 kJ/mol) and weakly (Δ*G* > −0.5 kJ/mol) bound or strongly (chemical shift of proton resonance δ_H_ = 4–6 ppm) and weakly associated (δ_H_ = 1–2 ppm) waters, the interaction energy between polymers and NPNP, etc. [7,28,32]. Thus, theoretical modeling provides a deeper insight into various phenomena occurring at a surface of simple and composite adsorbents.

**Figure 12:**
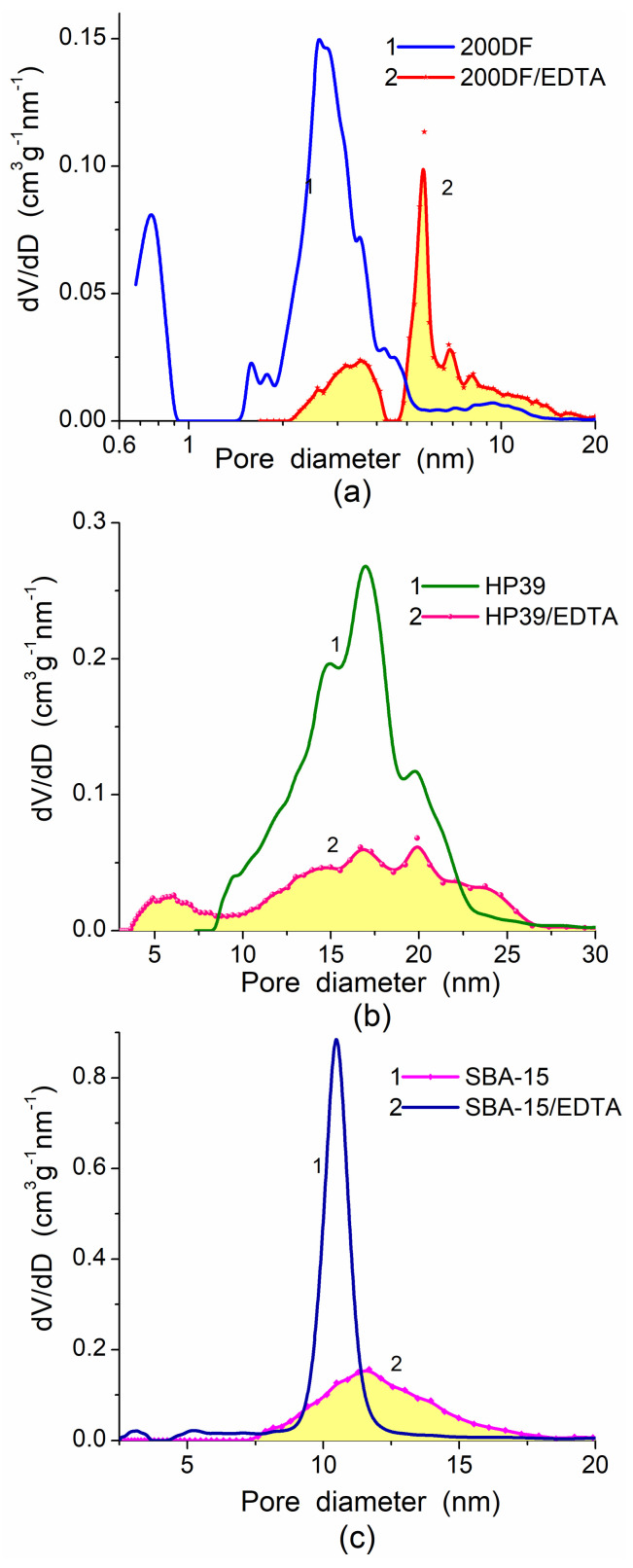
Differential NLDFT PSD of Crosfield silica gels (**a**) 200DF and (**b**) HP39, and (**c**) SBA-15 [109,110] initial and modified by *N*-(triethoxysilylpropyl)ethylenediaminetriacetic acid (EDTA) (Gelest, Morrisville, USA).

**Figure 13:**
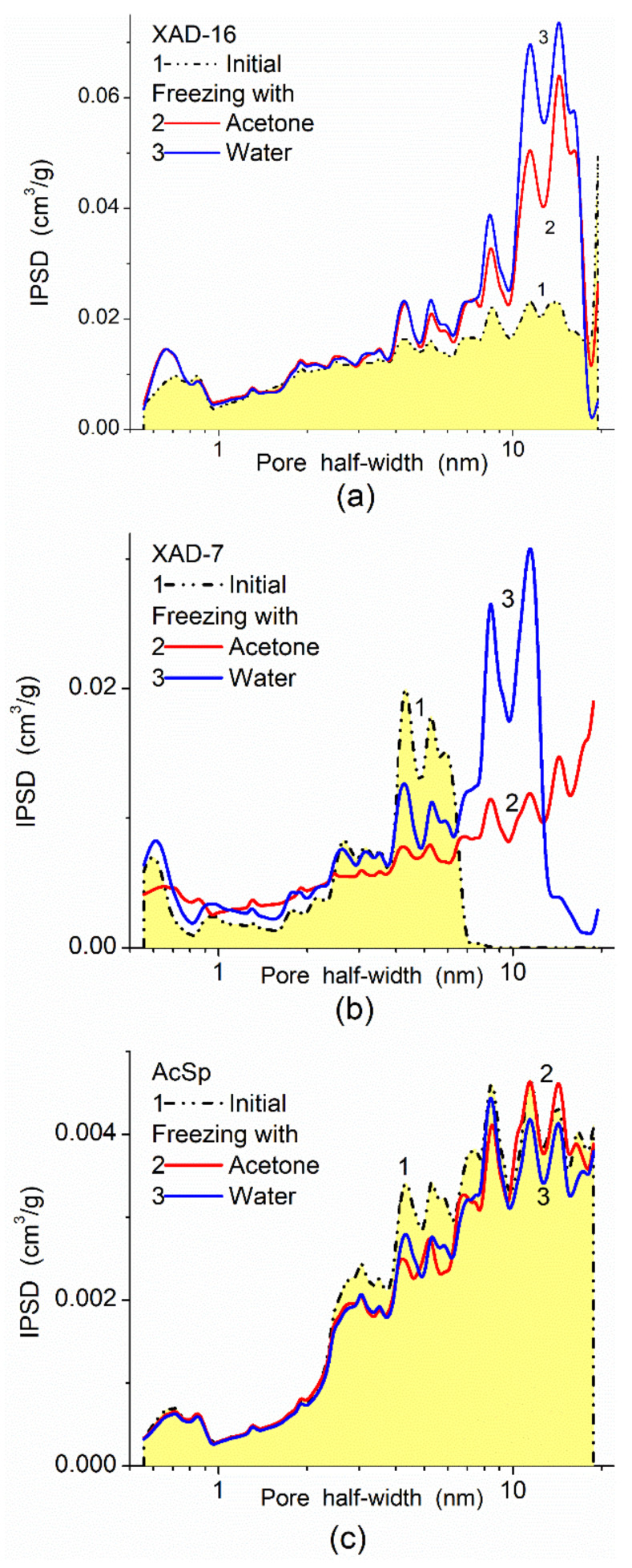
NLDFT IPSD of porous polymers initial and frozen with water of acetone (polymers were treated with liquids for 24 h, then frozen by liquid nitrogen for 2 h, and then unfrozen and degassed before nitrogen adsorption): (**a**) Amberlite XAD-16 (polystyrene, Fluka), (**b**) Amberlite XAD-7 (acrylic ester polymer, Fluka), and (**c**) AcSp (UMCS, Lublin, [31]) is a copolymer of a mixture of 4,4′-diphenyl sulfone dimethacrylate and 4-hydroxydiphenyl sulfone with divinylbenzene.

There is another textural/structural aspect related to the pore accessibility for adsorbed probes or target compounds upon chemical modification of a surface of adsorbents by low- or high-molecular-weight compounds. The surface functionalization could be used to improve the adsorption characteristics concerning certain target adsorbates (both from gaseous and liquid media), as well as to improve compatibility with filled polymers, to change their mechanical and other characteristics and properties. Typically, any chemical modification (functionalization) of porous adsorbents results in the diminution of their textural characteristics, such as PSD (Figure 12), and contributions of nano-, meso, and macropores to the specific surface area, *S*_BET_ and pore volume, *V*_p_ (Table 2). For example, for modified silica gel 200DF/EDTA (EDTA is *N*-(triethoxysilylpropyl)ethylenediaminetriacetic acid), *S*_nano_ decreases from 289 m^2^/g (for initial 200DF at *S*_BET_ = 484 m^2^/g) to 35 m^2^/g, and *V*_nano_ decreases from 0.05 to 0.02 cm^3^/g. Thus, the adsorption capacity of functionalized porous silicas can be more strongly decreased for the adsorbents with a significant contribution of nanopores (similar to 200DF) in contrast to the adsorbents with broader pores (similar to HP39 or SBA-15), which are characterized by a significant loss of the porosity, but it is not catastrophic [7,109,110]. Certain effects of the textural instability of polymers can be due to the significant contribution of narrow pores at a high value of the specific surface area (e.g., comp. AcSp and XAD-7 or XAD-16, Table 3, Figure 13).

The textural characteristics of porous polymers can be unstable depending on their composition and crosslinking degree, as well as on conditions. To demonstrate these effects, the characteristics of polymer beads (Table 3) were studied for initial samples and after suspending in water or acetone for 24 h and freezing by liquid nitrogen (77.4 K) for 2 h, then heated to room temperature and degassed before the nitrogen adsorption [31]. Additionally, some polymers (Table 4) were differently treated with water: washed–sonicated, washed, washed–sonicated–swollen, washed–swollen, and washed–sonicated–swollen–frozen. All the polymer samples were degassed (10^−3^ Torr) at 353–373 K for ca. 4 h before the nitrogen adsorption measurements. Since polymers XAD-7 and XAD-16 are characterized by the greatest structural changes due to washing–swelling–freezing, other treatment conditions have also been applied (Table 4): adding some quantity of water to a polymer, shaking in an ultrasonic bath (~2 min), storage for 24 h, freezing in liquid nitrogen, filtration, drying under mild conditions then at 353 K in the air; and degassing in vacuum at 353 K before nitrogen adsorption/desorption measurements. The treatments of some porous polymers under different conditions can strongly change their textural characteristics if they can be swollen and their 3D network is not enough strength (e.g., due to a low degree of crosslinking). However, a high value of *S*_BET_ and a significant contribution of narrow pores could not always result in textural instability (see Figure S12c). LiChrolut EN composed of poly(ethylvinylbenzene/divinylbenzene) is relatively stable (due to rigid crosslinking) upon wetting–freezing–drying, despite high *S*_BET_ = 1512 m^2^/g with a great contribution of nanopores at *S*_nano_ = 1024 m^2^/g [111].

Lignins, polymer/lignins and other related composites are of interest from a practical point of view [112,113]. However, the analysis of their texture is not a simple task. Therefore, a set of methods could be used in parallel, such as direct (TEM, SEM) and indirect (probe adsorption, SAXS, NMR cryoporometry, DSC thermoporometry, etc.) methods [9,28,31,54,55,56,64,65,66,74,75,76,77,78,79,80,81,82,83,84,85,86,87,111,114,115,116,117,118,119,120,121,122,123,124,125,126,127,128,129,130]. More accurate results can be obtained if several methods are used in parallel (e.g., SAXS and adsorption).

**Figure 14:**
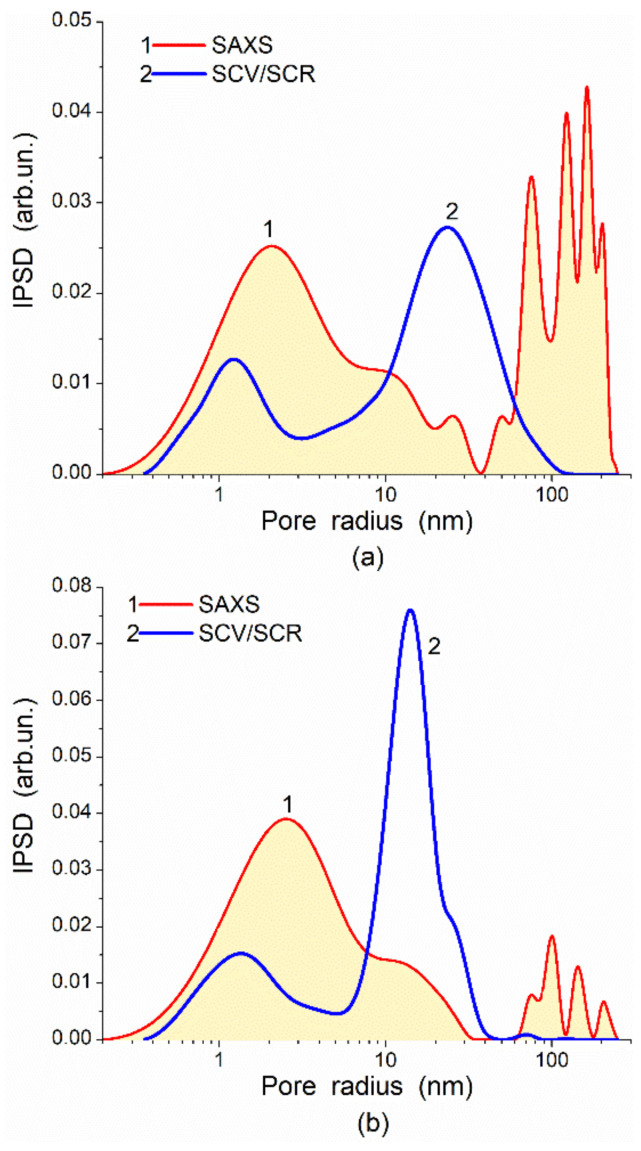
Incremental pore size distributions calculated from nitrogen adsorption–desorption isotherms and SAXS data for samples (**a**) kraft lignin (M_W_ = 1300 Da)/PDVB (2:5) (*S*_BET_ = 409 m^2^/g, *S*_SAXS_ = 1146 m^2^/g, *V*_p_ = 0.9 cm^3^/g) and (**b**) PDVB (*S*_BET_ = 542 m^2^/g, *S*_SAXS_ = 1286 m^2^/g, *V*_p_ = 1.3 cm^3^/g) (some details on the materials are given elsewhere [55]).

To change the adsorption characteristics of polymers, e.g., nonpolar polymers, such as poly(divinylbenzene) (PDVB), polystyrene (PS), copolymers of divinylbenzene and styrene (PDVBS), they could be filled not only by solid fillers (metal oxides, carbons, etc.) but also by polymers of various nature, e.g., polar polymers, such as various lignins, polyurethanes, poly(hydroxyethyl methacrylate), etc. For composites, there is a certain decrease in the *S*_BET_ value for lignin/PDVB as Δ*S*_BET_ = −133 m^2^/g and for Δ*S*_SAXS_ = −140 m^2^/g. However, *S*_SAXS_ >> *S*_BET_ due to the significant contribution of closed nanopores and narrow mesopores at *R* < 10 nm in polymer particles (Figure 14). Condition *S*_SAXS_ >> *S*_BET_ for these polymers (Figure 14) is similar to that for chars and AC (Figure 1). This result could be explained by the strong compaction of neighboring polymer chains in particles but without chemical bonds between them. Non-crosslinked chains containing aromatic rings are located close to other similar (concerning closed pores) carbon structures in neighboring graphene planes without chemical bonding. Therefore, probe (nitrogen) molecules cannot penetrate in narrow voids between neighboring polymer chains. Thus, a surface of weakly spatially separated polymeric chains or graphene sheets in adsorbent particles is inaccessible for nitrogen molecules, but it can take part in the X-ray scattering as separated structures giving a contribution to the *S*_SAXS_ values.

The difference in the *S*_SAXS_ and *S*_BET_ values allows one to evaluate a possible increase in the specific surface area due to certain activation, e.g., burn-off activation of carbons (Figure 1) or washing–swelling–freezing–drying of polymers (Table 3 and Table 4, Figure 13 and Figure 14). Additionally, some results of swelling of soft polymeric structures affecting the textural characteristics can be observed using NMR cryoporometry or DSC thermoporometry (e.g., for cryogels or hydrogels). These effects can also appear upon ions’ adsorption, low-molecular-weight compounds from the gaseous (vapor) phase (e.g., water, see Figures S14–S16), or solutes from liquid media. This is because the polymeric adsorbents or related composites can demonstrate a much greater swelling degree in aqueous media than oxide or carbon adsorbents. Thus, to analyze the complex phenomena for composites being in different dispersion media, a maximum large set of methods should be used.

## 4. Conclusions

Thus, accurate textural and morphological characterization of relatively simple but nonuniform materials, such as metal and metalloid oxides, carbons, polymers, etc., is not a simple task due to various factors related to the experiments per se and the treatment methods of the experimental data [1,2,93,131,132,133,134]. This task becomes much more complex for composites and hybrids, such as polymers/fillers, carbon, metal or metal oxides/polymers, nonuniform composite blends with different phases, etc. [28,54,55,56,85,86,87,88,89,90]. Therefore, to separate the results with large and small systematic errors, a set of various methods should be used for accurate morphological, textural, and structural characterization of composites and hybrid systems, considering all their components, as well as interactions of these components in composites [28,89,90,91].

This study’s main aim was to show some ways to improve information extraction for more complete and accurate characterization of complex adsorbents, polymer fillers, and composites of different kinds. This could be provided by using the models, which correctly describe each component in composites concerning material kinds and shapes of pores and particles, and by using the self-consistent regularization procedures for simultaneously solving a set of related integral equations [89,90,91]. For effective data treatments and maximal information extraction, it is of importance to use appropriate methods to treat the experimental (especially indirect) data with accurate physicomathematical models of the experimental dependences on pressure, concentration, temperature, and other parameters and related computational programs, e.g., with the SCR procedures. Besides this way, a set of additional methods may be used. Among these methods are electron microscopies (TEM, SEM), small-angle X-ray scattering (SAXS), X-ray diffraction, cryoporometry, relaxometry, thermoporometry, quasi-elastic light scattering, Raman and infrared spectroscopies and some others. Note that even infrared spectroscopy could be used for the textural characterization of silica-containing materials [135].

Thus, a more accurate description of complex and hybrid materials is possible if several methods complemented one another are used in parallel, e.g., adsorption and SAXS with the SCR procedures giving pore size, pore wall thickness, and particle size distributions functions for each component with consideration of the influence of one component onto others; as well as the specific surface area of open and closed pores, TEM/SEM/CLSM with quantitative treatments of images giving the PaSD, PSD, and PWTD functions for whole composite and each component. Some other methods, such as cryo- and thermoporometry and relaxometry, may give information not only on the textural characteristics of composites but also on the structure and temperature and interfacial behaviors of adsorbates confined in pores. As a whole, the software complex developed for the treatment of various experimental data includes more than 200 programs for more than 30 experimental methods, and only a small part of these programs is described here. In future works, this software complex may be expanded to include more types of materials, pore and particle shapes, other experimental, theoretical, and applied mathematics methods, etc.

## Figures and Tables

**Table 1:** Textural characteristics of PMS alone and PMS/A-300.

Sample	*S*_BET_ (m^2^/g)	*S*_DFT_ (m^2^/g)	*S*_nano_ (m^2^/g)	*S*_meso_ (m^2^/g)	*S*_macro_ (m^2^/g)	*V*_p_ (cm^3^/g)	*V*_nano_ (cm^3^/g)	*V*_meso_ (cm^3^/g)	*V*_macro_ (cm^3^/g)	<*R*_V_> (nm)	<*R*_S_> (nm)
PMS	507	471	2	504	1	1.320	0.002	1.304	0.014	6.08	5.28
Stirred PMS	572	581	1	558	13	2.604	0.001	2.248	0.355	16.86	9.42
PMS/A-300	354	322	35	306	13	1.265	0.019	1.084	0.163	15.25	7.64
cPMS/A-300	407	357	8	399	1	1.021	0.006	1.005	0.011	6.56	5.17
A-300	294	289	44	229	16	0.850	0.023	0.567	0.259	20.41	6.14

Note: The values of *V*_nano_ and *S*_nano_, *V*_meso_ and *S*_meso_, and *V*_macro_ and *S*_macro_ were calculated by integration of the *f*_V_(*R*) and *f*_S_(*R*) functions at 0.35 nm < *R* < 1 nm, 1 nm < *R* < 25 nm, and 25 nm < *R* < 100 nm, respectively. The values of <*R*_V_> and <*R*_S_> as the average pore radii were calculated as a ratio of the first moment of *f*_V_(*R*) or *f*_S_(*R*) to the zero moment (integration over the 0.35–100 nm range) <*R*> = ∫*f*(*R*)*RdR*/∫*f*(*R*)*dR.*

**Table 2:** Textural characteristics of initial and modified silicas.

Silica	Modifier	*S*_BET_ (m^2^/g)	*V*_p_ (cm^3^/g)
200DF	-	484	0.31
200DF	APTS	28	0.06
200DF	Triamine	2	0.002
200DF	EDTA	178	0.25
HP39	-	449	1.96
HP39	EDTA	246	0.81
SBA-15	-	577	1.34
SBA-15	EDTA	286	0.89

Note: modifiers: APTS—3-aminopropyltriethoxysilane, Triamine—3-(trimethoxysilylpropyl) diethylenetriamine, EDTA—*N*-(triethoxysilylpropyl)ethylenediaminetriacetic acid.

**Table 3:** Structural characteristics of initial and treated porous polymers.

Sample	*S*_BET_ (m^2^/g)	$\frac{\Delta S_{BET}}{S_{BET}}\;(\%)$	*S*_nano_ (m^2^/g)	*S*_meso_ (m^2^/g)	*S*_macro_ (m^2^/g)	*V*_p_ (cm^3^/g)	$\frac{\Delta V_{p}}{V_{p}}\;(\%)$	*V*_nano_ (cm^3^/g)	*V*_meso_ (cm^3^/g)	*V*_macro_ (cm^3^/g)	Δ*w*_cyl_
AcSp ^a^	93	-	9	80	4	0.252	-	0.004	0.207	0.069	0.030
AcSp ^b^	86	−8.3	21	63	3	0.231	−7.5	0.007	0.190	0.058	−0.051
AcSp ^c^	85	−2.7	22	61	3	0.220	−8.6	0.007	0.184	0.058	−0.055
XAD-7 ^a^	341	-	72	269	-	0.440	-	0.030	0.409	0.002	0.041
XAD-7 ^b^	462	35.5	108	321	33	0.905	105.7	0.044	0.540	0.589	0.059
XAD-7 ^c^	488	43.1	109	378	1	0.798	81.4	0.044	0.767	0.015	0.054
XAD-16 ^a^	853	-	92	751	11	1.347	-	0.029	1.247	0.170	0.148
XAD-16 ^b^	982	15.1	119	860	3	1.889	40.2	0.037	1.825	0.047	0.142
XAD-16 ^c^	984	15.4	106	876	2	2.026	50.4	0.034	1.984	0.031	0.139

Note: Amberlite XAD-16—polystyrene (Fluka), Amberlite XAD-7—acrylic ester polymer (Fluka), and AcSp (UMCS, Lublin, [31]) is a copolymer of a mixture of 4,4′-diphenyl sulfone dimethacrylate and 4-hydroxydiphenyl sulfone with divinylbenzene. ^a^ Initial, suspended in ^b^ acetone or ^c^ water and frozen by liquid nitrogen for 2 h; micro- (*R* < 1 nm), meso- (1 < *R* < 25 nm), and macropores (*R* > 25 nm); Δ*w*_cyl_ is the deviation (average relative errors concerning the specific surface area) of the pore model with cylindrical pores in polymers [31].

**Table 4:** Structural characteristics of initial and treated XAD-7 and XAD-16.

Sample	*S*_BET_ (m^2^/g)	$\frac{\Delta S_{BET}}{S_{BET}}\;(\%)$	*V*_p_ (cm^3^/g)	$\frac{\Delta V_{p}}{V_{p}}\;(\%)$
XAD-7 ^a^	338	-	0.462	-
XAD-7 ^b^	444	31.4	0.610	32.0
XAD-7 ^c^	422	24.9	0.562	21.6
XAD-7 ^d^	466	37.9	0.623	34.8
XAD-7 ^e^	485	43.5	0.667	44.4
XAD-7 ^f^	421	24.6	0.564	22.1
XAD-16 ^a^	836	-	1.524	-
XAD-16 ^b^	932	11.5	1.824	19.7
XAD-16 ^c^	878	5.0	1.633	7.2
XAD-16 ^d^	936	12.0	1.844	21.0
XAD-16 ^f^	937	12.1	1.824	19.7

Note: ^a^ initial, ^b^ washed–sonicated, ^c^ washed, ^d^ washed–sonicated/swelled, ^e^ washed–swollen, and ^f^ washed–sonicated–swollen–frozen samples (all the samples were dried and degassed before nitrogen adsorption measurements) [31].

## Data Availability

The data presented in this study are available on request from the corresponding author.

## Supplementary Materials

The following are available online at https://www.mdpi.com/article/10.3390/polym13081249/s1. Figure S1: *S*_BET_ vs. maximal *p*/*p*_0_ for MCM-41, MCM-48, and SBA-15. Figure S2: PaSD for nanosilica based on SEM image and adsorption data. Figure S3: IPSD for initial and MCA-treated nanosilica. Figure S4: Nitrogen adsorption-desorption isotherms and PSD for fresh and aged A-500. Figure S5: IPSD for PDMS-modified A-300. Figure S6: PSD and nitrogen adsorption energy distributions (NAED) for alumina/silica. Figure S7: PSD and NAED for titania/silica. Figure S8: PSD and NAED for various carbons. Figure S9: PSD of various AC estimated from nitrogen and benzene adsorption. Figure S10: *S*_BET_ estimated for nanooxides using different probes. Figure S11: Effects of silica hydrophobization on NAED. Figure S12: PSD for treated polymers. Figure S13: PSD for treated AC. Figure S14: Water adsorption onto nanosilicas. Figure S15: *S*_Ar_ vs. silanol concentration for nanosilicas. Figure S16: Modeling of clustered adsorption of water. Figure S17: IR spectra of A-300. Figure S18: Decomposition of the IR spectrum of A-300 over the OH range. Figure S19: FTIR spectra of treated polymers. Figure S20: Infrared spectra of PMS and PDMS. Figure S21: SAXS and SCV/SCR PSD for a char. Figure S22: SAXS and SCV/SCR PSD for AC. Figure S23: SAXS and V/SCR PaSD for A-300. Figure S24: SAXS PaSD for a polymer and related char. Figure S25: Chord size distributions for a polymer and related char. Figure S26: SEM and TEM images of fresh and aged nanosilicas. Figure S27: AFM PaSD for AC and char/silica. Figure S28: TEM images of PMS and PMS/A-300. Figure S29: TEM PaSD of PMS and PMS/A-300. Figure S30: SEM images of PDMS-modified A-300. Figure S31: SEM PaSD of PDMS-modified A-300. Figure S32: PaSD of A-300 computed using different experimental data. Figure S33: NMR-cryoporometry PSD for nanosilica dispersions. Figure S34. NMR-cryoporometry PSD for treated nanosilica. Figure S35: DSC thermoporometry for different silicas. Figure S36: TSDC relaxometry PSD for nanosilica dispersions. Figure S37: TSDC relaxometry and SCV/SCR PSD for nanosilicas. Figure S38: TSDC relaxometry and N_2_ PSD for MCM-48. Figure S39: CLSM images, PSD and PWTD for a cryogel. Figure S40: Cluster models of silica nanoparticles. Figure S41: Models of nonporous and porous particles of silica. Figure S42: HRTEM image and cluster model of AC. Figure S43: Interaction energy vs. charge transfer for probes interacting with nanosilicas. Figure S44: Theoretical ^1^H NMR spectra of silica with different adsorbates. Figure S45: Theoretical ^1^H NMR spectra of water bound to nanoparticles. Figure S46: Models of hydrophobic AM1, PMS and PDMS. Figure S47: Theoretical ^1^H NMR spectra of water and methane bound to silicas. Figure S48: MD model of water bound to silica vs. temperature. Figure S49: MD calculations of dehydration of silicas. Figure S50: MD calculations of dehydration of silica gel. Table S1: Textural characteristics of PMS and PMS/A-300. Table S2: Adsorption energy and charge transfer for probes adsorbed onto silica (DFT). Table S3: Adsorption energy of various probes bound to silica and AC (PM7).
