# Supplementary material for: Polymer Adsorbents vs. Functionalized Oxides and Carbons: Particulate Morphology and Textural and SurfaceCharacteristics
Source: Polymers (Basel). 2021 Apr 12;13(8):1249. doi: 10.3390/polym13081249 (PMC8069040; doi:10.3390/polym13081249)
Supplement: Supplementary file 1 [file polymers-13-01249-s001.pdf]

polymers

MDPI

Article

# Supplementary Materials

# Polymer adsorbents vs. functionalized oxides and carbons: particulate morphology and textural and surface characterization

**Volodymyr M. Gun'ko***

Chuiko Institute of Surface Chemistry, 17 General Naumov Street, 03164 Kyiv, Ukraine; vlad_gunko@ukr.net

* Correspondence: vlad_gunko@ukr.net; Tel.: +38044 4229627 (V.M.G.)

## S1. Adsorption

Typically, to estimate the textural characteristics of samples degassed at increased temperature (350–470 K for several hours), low-temperature (77.4 K) nitrogen (or argon) adsorption–desorption isotherms are recorded using an adsorption analyzer (e.g., Micromeritics ASAP 2010, 2020, or Quantachrome Autosorb). The specific surface area ($S_{BET}$) is calculated (using the adsorption isotherms) according to the BET method [1], which, however, has some disadvantages causing some systematic errors [1,2]. For example, the $S_{BET}$ value depends strongly on the pressure range used (Figure S1) due to (i) unfinished monolayer adsorption at low $p/p_{0,max}$ values or started multilayer adsorption at larger $p/p_{0,max}$ values and these changes could depend on the types of both adsorbent and adsorbate; (ii) changes in an occupied area of a surface by a probe (nitrogen) molecule depending on pore sizes and structure of a solid surface (e.g., the content of hydroxyls or other surface functionalities affecting the orientation of adsorbed $N_2$ molecules), etc. Additionally, the dependence is stronger for adsorbents with narrower pores (Figure S1). Therefore, the specific surface area of adsorbents should be estimated using not only the adsorption method but also SAXS, cryoporometry, etc., which, however, can generate other systematic errors.

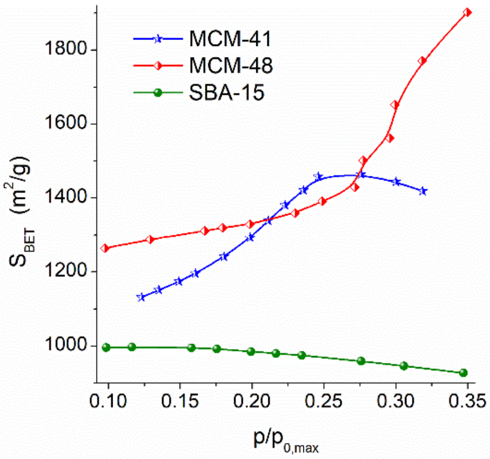


**Figure S1.** The value of $S_{BET}$ as a function of the maximal $p/p_0$ value of the pressure range (0.05– $p/p_{0,max}$) used upon calculation of the $S_{BET}$ values for mesoporous ordered silicas MCM-41, MCM-48, and SBA-15.

The total pore volume ($V_p$) could be evaluated from the nitrogen adsorption at $p/p_0 \approx 0.98$–0.99, where $p$ and $p_0$ are the equilibrium and saturation pressure of nitrogen at 77.4 K, respectively [2]. However, if an adsorbent possesses macropores, especially broad ones, the $V_p$ value can be strongly underestimated. For example, loose powders with fumed oxides of metals or metalloid (FMO) composed of nonporous nanoparticles (NPNP) (3–120 nm in size depending on the FMO types) forming aggregates (< 1 μm in size) and agglomerates of aggregates (> 1 μm) are characterized by a low bulk density $\rho_b$ = 0.04–0.15 g/cm$^3$ [3,4]. The low bulk density corresponds to a very large empty volume in the loose powders $V_{em} = 1/\rho_b - 1/\rho_0$ (e.g., 24.5 cm$^3$/g for A-300 at $\rho_0$ = 2.2 g/cm$^3$ and $\rho_b$ = 0.04 g/cm$^3$), which is greater by 10–20 times than the $V_p$ values of FMO because nitrogen cannot completely fill macropores due to very weak interactions with distant pore walls (NPNP surface). Additionally, for "soft" (e.g., polymeric) adsorbents, adsorption of nitrogen fluid can lead to swelling that results in open hysteresis loops. Upon the adsorption of liquids (e.g., water) more strongly interacting with adsorbents, even solid adsorbents, such as fumed silica, can demonstrate slow swelling of nanoparticles [3–7]. There

are the opposite effects upon strong heating of FMO because removal of intact water and surface hydroxyls leads to a decrease of NPNP sizes ($S_{BET}$ increases), and then NPNP sintering results in a decrease in the $S_{BET}$ value. All these effects related to the prehistory of samples can change the final results, and sometimes these results could seem unexpected.

The nitrogen adsorption–desorption isotherms could be used to compute the pore size distributions (PSD, differential $f_V(R)$ ~d$V_P$/d$R$ and $f_S(R)$ ~d$S$/d$R$) using various approaches. These approaches could include various systematic errors caused by an inappropriate model of pores (e.g., cylindrical pores poorly modeling voids between NPNP in aggregates of FMO), inappropriate parameters of solids (e.g., parameters of carbons poorly describing polymeric adsorbents), etc. Therefore, for materials with a complex topology of pores or/and composed of several different phases (e.g., FMO and polymers or carbons, etc.), firm (Micromeritics, Quantachrome, etc.) software can give incorrect results with systematic errors. Better results could be obtained using a complex pore model with slit-shaped (S) and cylindrical (C) pores and voids (V) between spherical nanoparticles (SCV method) with the corresponding parameters for the different phases [8,9]. Additionally, the chemical structure of a solid surface (e.g., hydroxyls or other functionalities) can affect the interactions (and orientation, i.e., effective area of a surface occupied by a molecule) of nitrogen or other probe molecules with a surface. The SCV method with a self-consistent regularization (SCR) procedure [9] allows one to consider the presence of several phases since the parameters of several types of surfaces (e.g., silica, alumina, titania, carbon, carbohydrate polymers, etc.) could be simultaneously used with an appropriate pore model for each component. The SCR procedure gives information on contributions (weight coefficients) of different pore types and different components into the total porosity and specific surface area. As a whole, the model errors remain upon the use of the SCV/SCR method because the texture of any adsorbent is not strongly ordered (pores can have very complex shape), there is surface roughness, etc. However, the SCV/SCR method reduces the systematic errors appearing upon the application of the firm software for complex materials. Note that the PSD could be calculated using molecular density functional theory (DFT) methods, such as nonlocal DFT (NLDFT), quenched solid DFT (QSDFT), 2D-NLDFT, well-developed modified Nguyen–Do (MND) method or others.

For better view of the PSD at large values of $R$, the differential PSD concerning the pore volume $f_V(R)$ ~d$V$/d$R$, $\int f_V(R)dR$ ~$V_P$ could be recalculated to incremental PSD (IPSD) at $\Phi_V(R_i) = (f_V(R_{i+1}) + f_V(R_i))(R_{i+1} - R_i)/2$ at $\sum\Phi_V(R_i) = V_P$). The $f_V(R)$ and $f_S(R)$ functions could also be used to calculate contributions of nanopores into the total pore volume and specific surface area ($V_{nano}$ and $S_{nano}$ at the radius in the range 0.35 nm < $R$ < 1 nm), mesopores ($V_{meso}$ and $S_{meso}$ at 1 nm < $R$ < 25 nm), and macropores ($V_{macro}$ and $S_{macro}$ at 25 nm < $R$ < 100 nm). Clear, an incorrect PSD results in incorrect values of the textural characteristics.

The average values of the pore radii could be determined concerning the pore volume ($X = V$) and the specific surface area ($X = S$), respectively, as the corresponding moments of the distribution functions

$$< R_X > = \int_{R_{min}}^{R_{max}} R f_X(R) dR \Big/ \int_{R_{min}}^{R_{max}} f_X(R) dR. \qquad \text{(S1)}$$

Additionally, $f_S(R)$ could be used to estimate the deviation ($\Delta w$) of the pore shape from the model using [8,9]

$$\Delta w = S_{BET} \Big/ \int_{R_{min}}^{R_{max}} f_S(R) dR - 1, \qquad \text{(S2)}$$

where $R_{max}$ and $R_{min}$ are the maximal and minimal pore radii, respectively. The $S^*_{nano}$, $S^*_{meso}$ and $S^*_{macro}$ values could be corrected by multiplication by ($\Delta w$+1) that gives $S^*(\Delta w + 1) = S_{sum} = S_{nano} + S_{meso} + S_{macro} = S_{BET}$. The effective $w$ value ($w_{ef}$) can be estimated with equation

$$w_{ef} = (S_{BET} / V_p) \int_{R_{min}}^{R_{max}} R f_V(R) dR \Big/ \int_{R_{min}}^{R_{max}} f_V(R) dR \cdot \qquad \text{(S3)}$$

However, the reliability of the $\Delta w$ value depends on the reliability of both $S_{BET}$ and PSD.

The PSD functions could also be calculated using various density functional theory (DFT) methods, such as nonlocal DFT (NLDFT), quenched solid DFT (QSDFT), 2D-NLDFT, etc. (present in firm (Micromeritics, Quatachrome, etc.) software) or DFT version with overall equation [9–11]

$$W(p) = v_M \left[ \int_{\sigma_{ss}/2}^{r_k(p)} \rho_f(R) f(R) dR + \int_{r_k(p)}^{R_{max}} \frac{t}{R - \sigma_{ss}/2} \rho_M(R) f(R) dR \right] \qquad \text{(S4)}$$

where $W$ is the adsorption, where $v_M$ the liquid molar volume, $\rho_f$ the fluid density in occupied pores, $\rho_m$ the density of the multilayered adsorbate in pores, $r_k$ the radius of pores occupied at the pressure $p$, $\sigma_{ss}$ is the collision diameter of the surface atoms. To calculate the density of a gaseous adsorbate (nitrogen) at a given pressure $p$, Bender equation [12] could be used. The transition from gas (subscript g) to liquid (l) or fluid in the form of multilayered adsorbate in pores (m) can be linked to the corresponding fugacity $f$:

$$\ln\frac{f(T,\rho)}{R_gT\rho} = \frac{p(T,\rho)}{R_gT\rho} - 1 + \frac{1}{R_gT}\int_0^{\rho}[p(T,\rho) - R_gT\rho]\frac{d\rho}{\rho^2} \tag{S5}$$

and

$$f_{l,m} = f_g \exp(\frac{E_{i,m}}{RT}), \tag{S6}$$

where $E$ is the interaction energy of an adsorbate molecule with the pore walls and neighboring molecules calculated with the Lennard–Jones potentials. Thus, advantages of the SCV/SCR method are the possibilities to estimate (i) contributions of several phases of complex materials into the textural characteristics; (ii) adequate PSD of complex adsorbents; (iii) both PSD and particle size distribution (PaSD); (iv) errors of the model used that could correspond to a surface roughness of adsorbents [8–10].

To better describe the porosity of various adsorbents, an additional regularizer could be derived with maximum entropy principle [13] applied to the distribution function $f(R)$ that can be written as $N$-dimension vector ($N$ is the number of the grid points for $f$). This procedure was used to modify the CONTIN algorithm [14] (CONTIN/MEM-$j$ where $j$ denotes the order of $\vec{p}^{\,j}(\vec{f})$) and applied to study different silicas [15].

The specific surface area ($S_\varphi$) of materials composed of spherical nanoparticles characterized by the particle size distribution, PaSD $\varphi(a)$ (normalized to 1), can be calculated with equation

$$S_\varphi = \int_{a_{\min}}^{a_{\max}} \frac{3}{2a^3\rho}\left[2(a+t)^2 + Nr_m \arcsin\left(\frac{a}{A}\right)\sqrt{A^2-a^2} - N(a+t)(\frac{ar_m}{A}+t)\right]\varphi(a)da \tag{S7}$$

where $A = a + t + r_m$, $a$ is the particle radius $\rho$ the density of the material, $N$ the average coordination number of nanoparticles in aggregates, $t$ the thickness of an adsorbed nitrogen layer, and $r_m$ is the meniscus radius determined at $0.05 < p/p_0 < 0.2$, corresponding to the effective radius $R$ of voids between spherical particles. Condition $S_\varphi = S_{BET}$ can be used to estimate the $N$ value in Equation (S7).

To calculate the adsorption energy distribution functions, the Fowler–Guggenheim (FG) equation was used to describe localized monolayer adsorption with lateral interactions:

$$\theta_i(p,E) = \frac{Kp\exp(zw\Theta/k_BT)}{1+Kp\exp(zw\Theta/k_BT)}, \tag{S8}$$

where $K = K_0(T)\exp(E/k_BT)$ are the Langmuir constant for adsorption on energetically uniformed sites and the pre-exponential factor $K_0(T)$ is expressed in terms of the partition functions for isolated gas and surface phases, $z$ is the number of nearest neighbors of an adsorbate molecule (assuming $z = 4$), $w$ is the interaction energy between a pair of nearest neighbors, $k_B$ is the Boltzmann constant, e.g., $zw/k_B = 380$ K for nitrogen. The right term of Equation (S8) was used as the kernel in the overall adsorption isotherm equation to calculate the distribution function $f(E)$ of the nitrogen adsorption energy.

Any mechanical action on FMO results in certain changes in the textural characteristics of soft powders [8,16] (Figures S2–S5). However, mechanochemical activation (MCA in a ball-mill for several hours) of wetted nanosilica weakly affects the PaSD of NPNP (Figure S2) as well as long aging (Figure S4), but the structure of secondary particles (NPNP aggregates and agglomerates) strongly changes due to compaction of the powder that is well seen in changes in the PSD (Figure S3). These changes increase with increasing MCA time that results in enhancement of the bulk density with decreasing pore volume $V_p$ at $t_{MCA} = 6$ h. However, the $V_p$ value increases at $t_{MCA} = 1$ h on the initial stage of the compaction, despite the value of $V_{em}$ monotonically decreases from 21.8 cm$^3$/g (initial A-300) to 4.2 cm$^3$/g ($t_{MCA} = 1$ h) and 2.08 cm$^3$/g (6 h), but the inequality $V_{em} > V_p$ remains. Note that the IPSD with DFT (model of voids between silica NPNP) and NLDFT (cylindrical pores in silica) quantitatively differ, but they are qualitatively similar since both show an increase in IPSD intensity with no shift of the main peaks at treatment time $t_{MCA} = 1$ h and a shift of the main peaks toward smaller $R$ values and a decrease in PSD intensity at $R > 10$ nm at $t_{MCA} = 6$ h (Figure S3). The textural characteristics strongly

change upon mixing of nanooxides with polymers (Figure S5, Table S1) or upon preparation of mixed oxides (Figures S6 and S7).

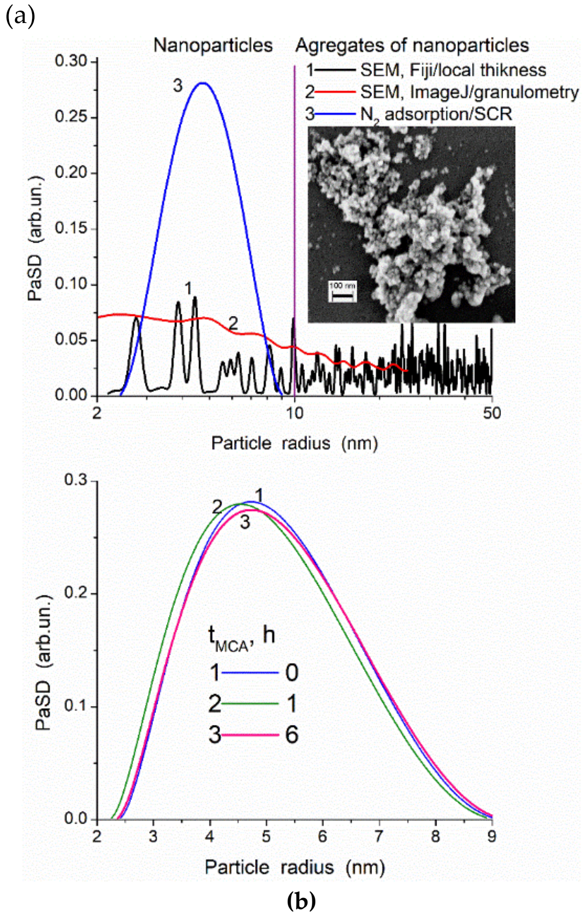


**Figure S2**. Particle size distribution (PaSD) for silica (a) initial calculated (with Fiji/local thickness plugin, curve 1, and ImageJ/granulometry plugin, curve 2) using SEM image and nitrogen adsorption isotherm (curve 3), and (b) primary particle size distributions for silica initial ($S_{BET}$ = 330 m²/g) and MCA-treated wetted powder for 1 h ($S_{BET}$ = 345 m²/g) and 6 h ($S_{BET}$ = 332 m²/g) calculated using nitrogen adsorption isotherms and data of nitrogen adsorption (V/SCR) method.

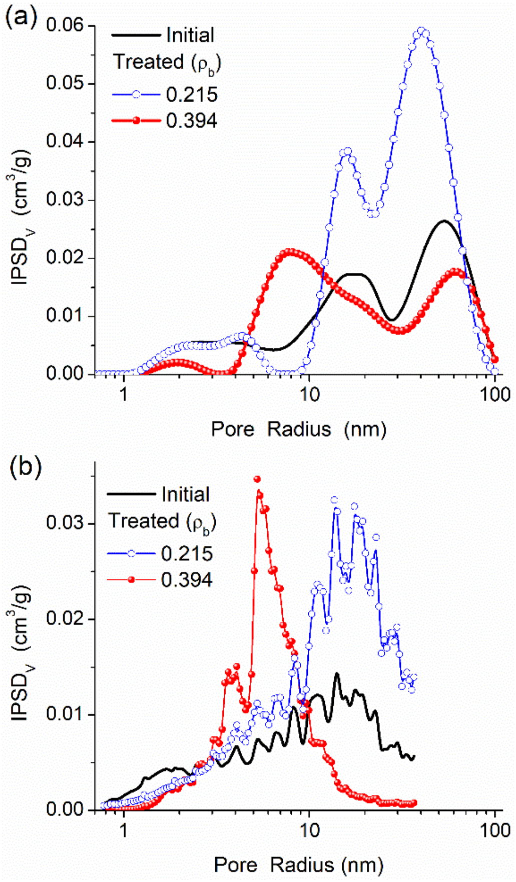


**Figure S3.** Incremental pore size distributions (IPSD) for initial ($V_P$ = 0.83 cm/g) and MCA-treated wetted powders for 1 h ($V_P$ = 1.42 cm/g) and 6 h ($V_P$ = 0.77 cm/g) concerning the pore volume calculated using (a) molecular density functional theory (DFT)/PaSD for the model of voids between spherical particles, and (b) nonlocal DFT(NLDFT) (cylindrical pores in silica).

**Table S1.** Textural characteristics of PMS alone and PMS/A-300.

| Sample | $S_{BET}$ (m²/g) | $S_{DFT}$ (m²/g) | $S_{nano}$ (m²/g) | $S_{meso}$ (m²/g) | $S_{macro}$ (m²/g) | $V_p$ (cm³/g) | $V_{nano}$ (cm³/g) | $V_{meso}$ (cm³/g) | $V_{macro}$ (cm³/g) | <$R_V$> (nm) | <$R_S$> (nm) |
|---|---|---|---|---|---|---|---|---|---|---|---|
| PMS | 507 | 471 | 2 | 504 | 1 | 1.320 | 0.002 | 1.304 | 0.014 | 6.08 | 5.28 |
| Stirred PMS | 572 | 581 | 1 | 558 | 13 | 2.604 | 0.001 | 2.248 | 0.355 | 16.86 | 9.42 |
| PMS/A-300 | 354 | 322 | 35 | 306 | 13 | 1.265 | 0.019 | 1.084 | 0.163 | 15.25 | 7.64 |
| cPMS/A-300 | 407 | 357 | 8 | 399 | 1 | 1.021 | 0.006 | 1.005 | 0.011 | 6.56 | 5.17 |
| A-300 | 294 | 289 | 44 | 229 | 16 | 0.850 | 0.023 | 0.567 | 0.259 | 20.41 | 6.14 |

Note: The values of $V_{nano}$ and $S_{nano}$, $V_{meso}$ and $S_{meso}$, and $V_{macro}$ and $S_{macro}$ were calculated by integration of the $f_V(R)$ and $f_S(R)$ functions at 0.35 nm < $R$ < 1 nm, 1 nm < $R$ < 25 nm, and 25 nm < $R$ < 100 nm, respectively. The values of <$R_V$> and <$R_S$> as the average pore radii were calculated as a ratio of the first moment of $f_V(R)$ or $f_S(R)$ to the zero moment (integration over the 0.35–100 nm range) $<R> = \int f(R)RdR / \int f(R)dR$.

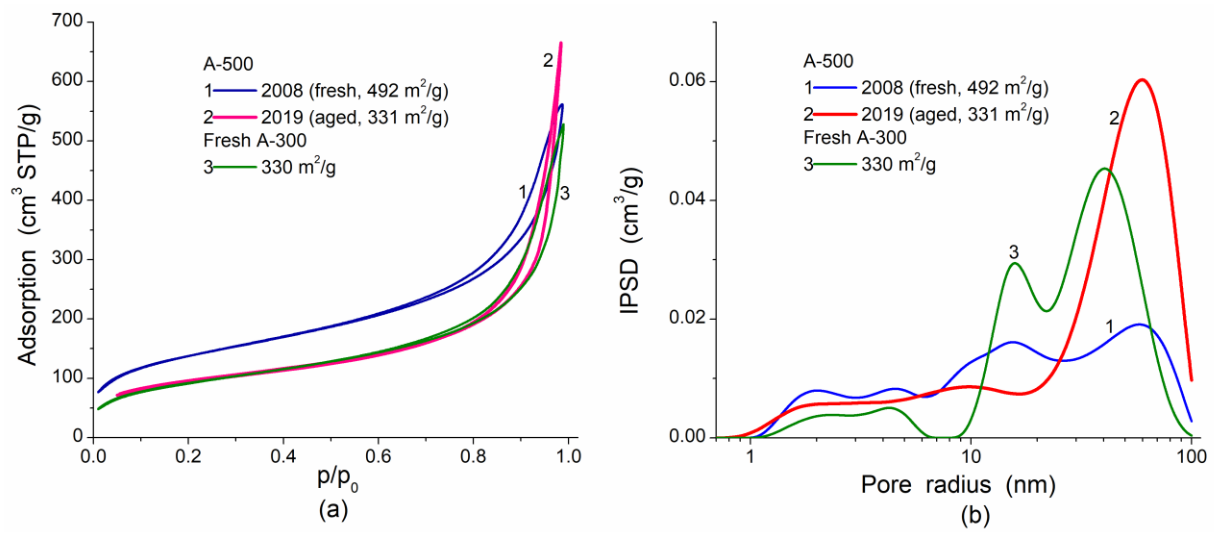


**Figure S4.** (a) Nitrogen adsorption–desorption isotherms for fresh (1) and 11 year-aged A-500 (2), and fresh A-300; (b) related V/DFT PSD (based on Eqs. (S4)–(S6)).

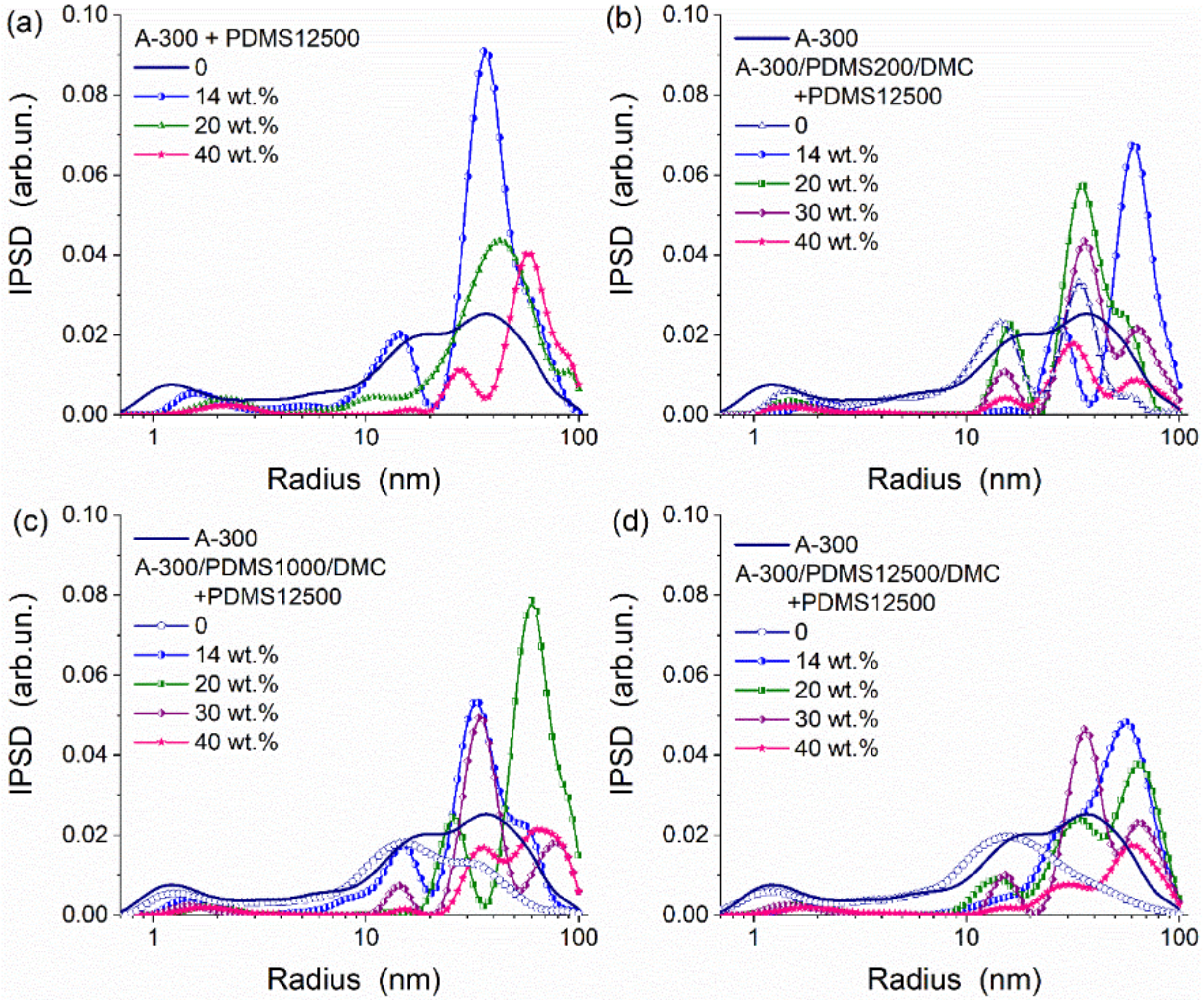


**Figure S5.** Incremental pore size distributions (IPSD) of (a) A-300 unmodified and with physically adsorbed PDMS12500, (b) A-300 unmodified and modified by PDMS200/DMC and with physically adsorbed PDMS12500, (c) A-300 unmodified and modified by PDMS1000/DMC and with physically adsorbed PDMS12500, (d) A-300 unmodified and modified by PDMS12500/DMC and with physically adsorbed PDMS12500 (amounts of physically adsorbed PDMS12500 are shown in Figure) (MND SCV/SCR method).

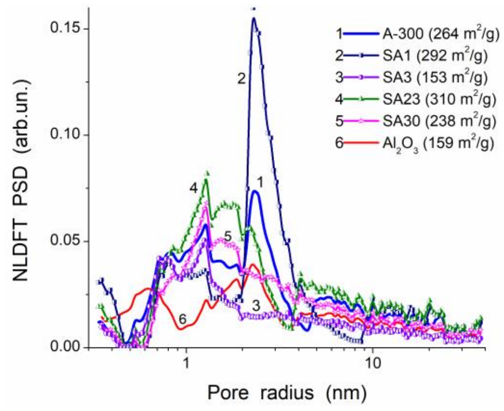


(a)

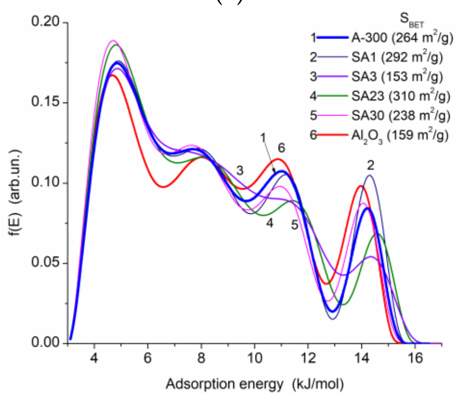


(b)

**Figure S6**. (a) PSD of silica (S), alumina (A), and mixed SA oxides (modified Nguyen–Do (MND) SCV/SCR method); (b) nitrogen adsorption energy using Fowler–Guggenheim kernel of an integral equation.

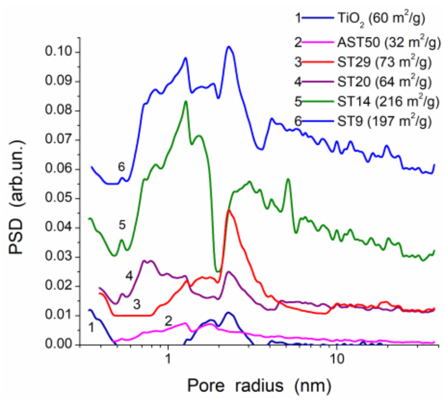


(a)

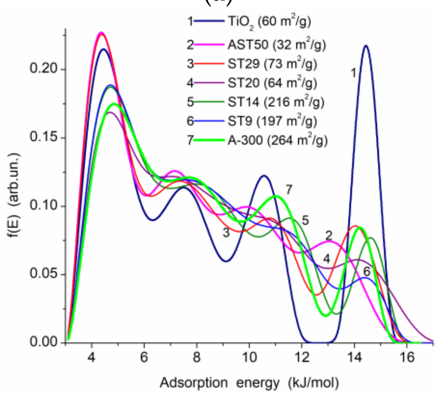


(b)

**Figure S7**. (a) NLDFT PSD (MND SCV/SCR method); (b) nitrogen adsorption energy distributions of titania and mixed silica/titania (ST).

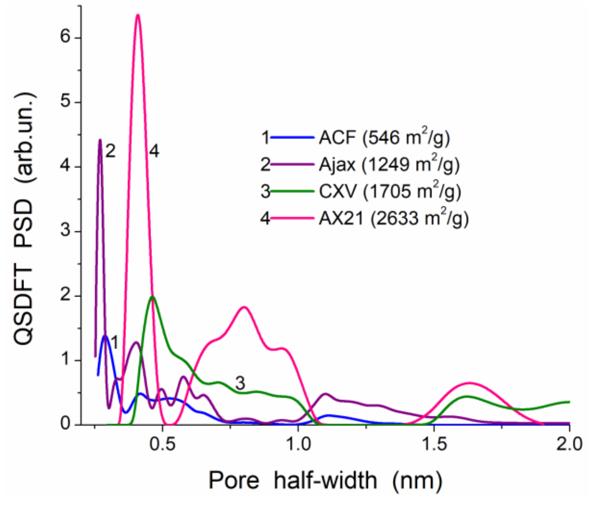


(a)

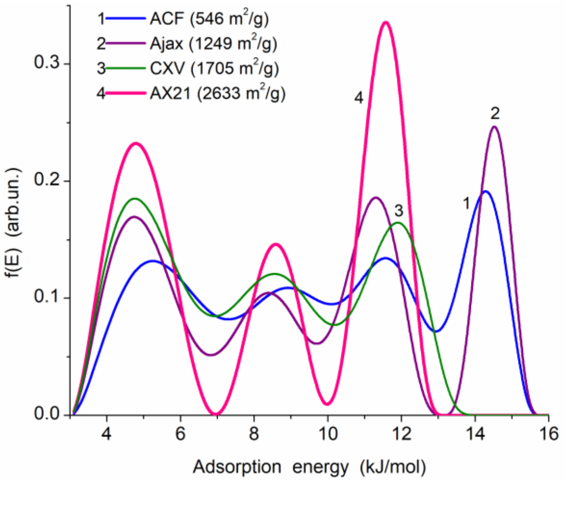


(b)

**Figure S8**. (a) QSDFT PSD; (b) nitrogen adsorption energy distributions of carbons.

There are significant textural changes in carbons with different degrees of activation affecting the PSD, *S* and *V* values and the adsorption energy distributions (Figures S8 and S9).

The effects of confined space, surface nature and the specific area, as well the nature and structure of adsorbates (X), strongly affect the results of adsorption concerning estimation of the specific surface area. The values of $S_{BET,X}$ could be overestimated (due to very strong interaction with active surface sites (strong Brønsted and Lewis acid surface sites in mixed fumed metal oxides, FMO) leading to conformational changes of adsorbed molecules) and underestimated (due to weak interaction of adsorbed molecules with weak surface sites, NPNP aggregation, which increases with decreasing size of FMO NPNP, reducing the accessibility of

the surface for larger molecules) (Figure S10). Note that the adsorption energy of nonpolar nitrogen molecules depends more strongly on the pore sizes than the chemical structure of a surface (Figure S11).

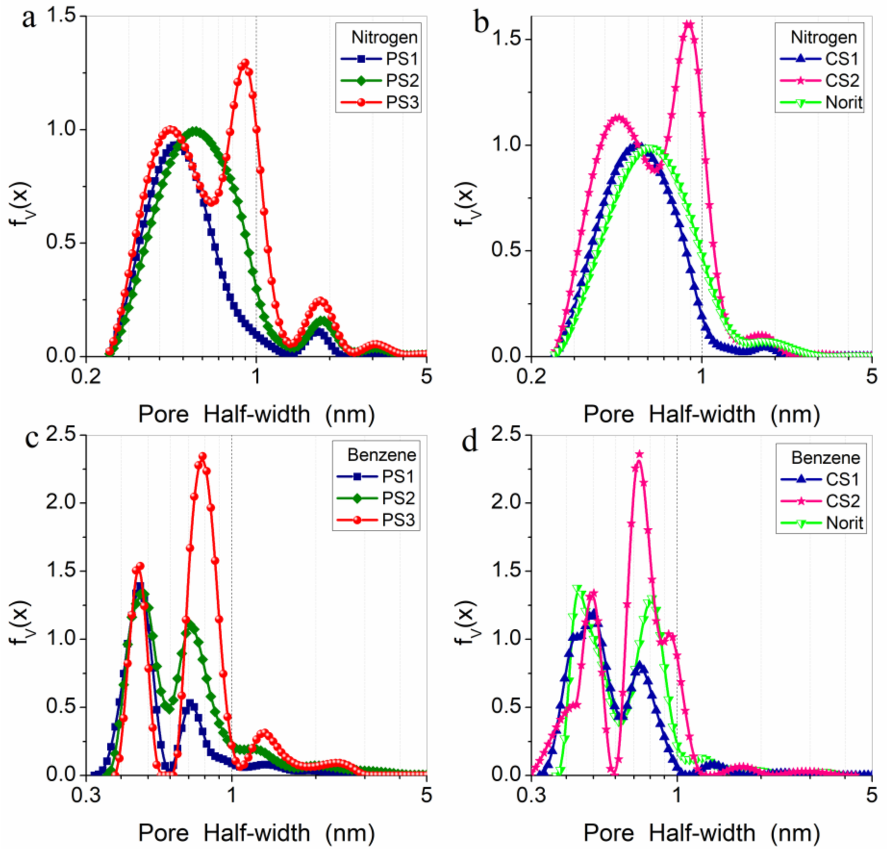


**Figure S9.** Textural characteristics of various AC estimated from nitrogen (a,b) and benzene (c,d) adsorption (PSD are probe dependent) (MND SCV/SCR method).

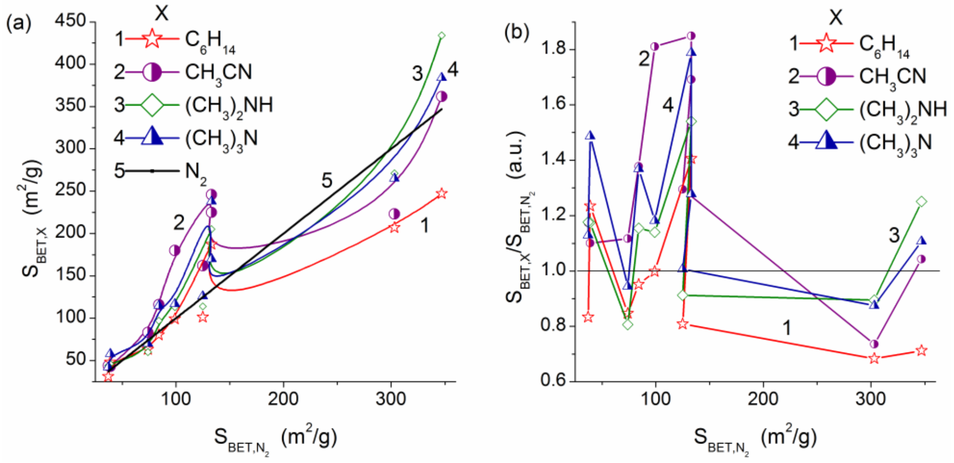


**Figure S10**. Specific surface area (*S*BET) vs. surface and adsorbed probe (X) structures: relationships between the $S_{BET,N2}$ and (a) $S_{BET,X}$ or (b) $S_{BET,X}/S_{BET,N2}$ values for silica, alumina, titania and mixed fumed oxides.

Various measurements could be carried out at low temperatures, and during these experiments, water or other liquids could be adsorbed and frozen at low temperatures $T < T_{fr}$ in pores of the materials studied than in the case of free bulk liquids. The effects of freezing of water (and other liquids, e.g., acetone) confined in pores of different materials can result in changes in the textural characteristics (Figures S12 and S13) [10], and these changes differ from the swelling results because the latter change the structure of the pore walls, but the former leads to increase in the pore volume and specific surface area (this is rather similar to the burn-off effects during activation of carbons). For some porous polymers, the effects are very strong (Figure S12a,b), but for others, they could be much smaller (Figure S12c). Note that the water adsorption has significant features (Figures S14–S18) compared to typical probe adsorbates, such as nitrogen, argon, or benzene [8].

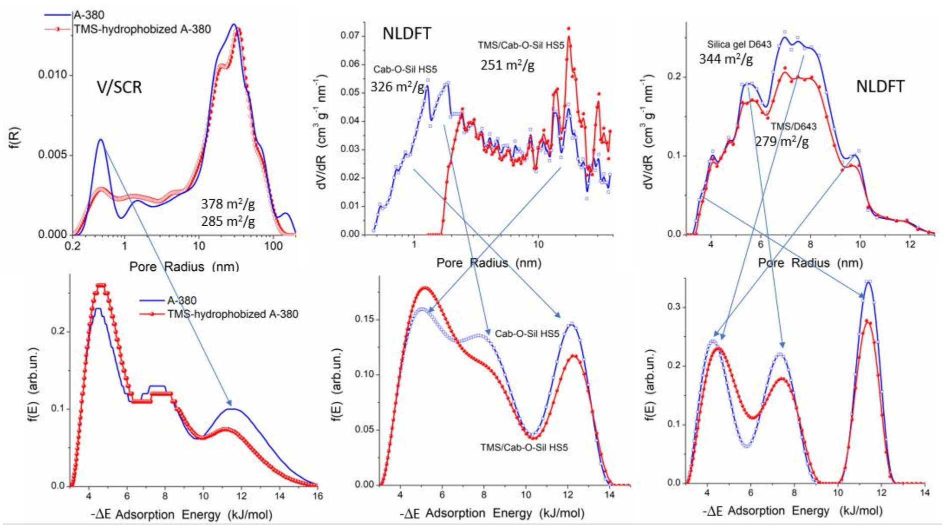


**Figure S11**. Effects of silica hydrophobization on nitrogen adsorption energy distributions.

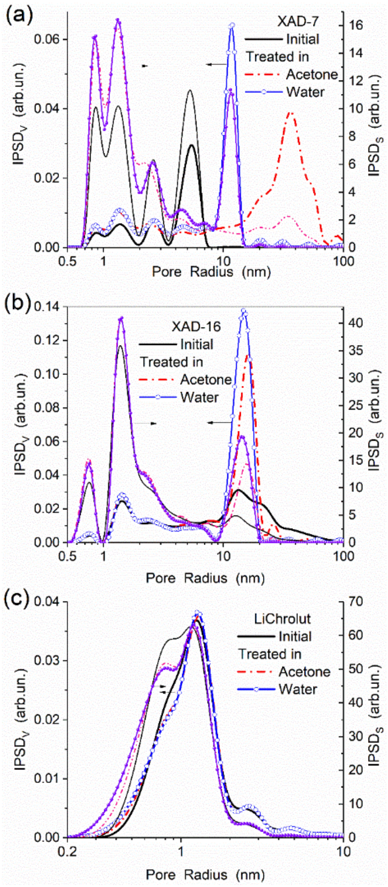


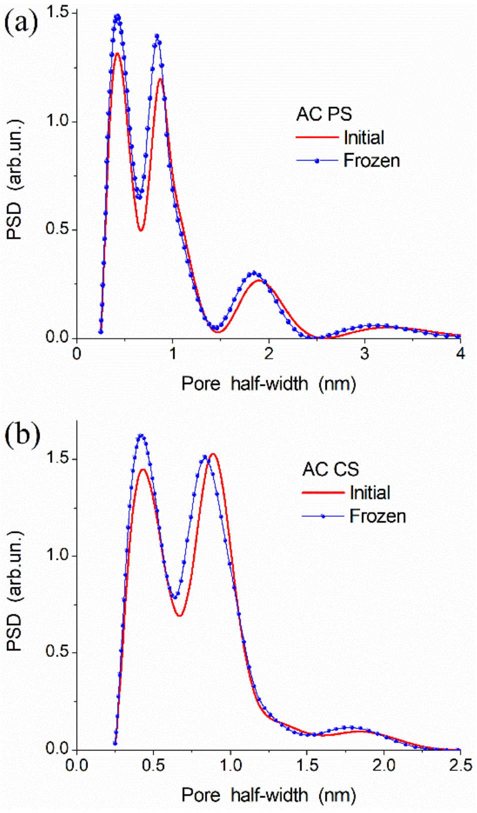


**Figure S12.** Pore size distributions concerning the pore volume (IPSDV) and specific surface area (IPSDS) of porous polymers initial and frozen with acetone or water (by liquid nitrogen for 2 h): (a) Amberlite XAD-7 (acrylic ester polymer, Fluka) (initial $S_{BET}$ = 341 m²/g, $V_P$ = 0.44 cm³/g, 2 h frozen (by liquid nitrogen) with acetone $S_{BET}$ = 462 m²/g, $V_P$ = 0.91 cm³/g, and with water $S_{BET}$ = 488 m²/g, $V_P$ = 0.80 cm³/g); (b) Amberlite XAD-16 (polystyrene, Fluka) (initial $S_{BET}$ = 853 m²/g, $V_P$ = 1.35 cm³/g, 2 h frozen (by liquid nitrogen) with acetone $S_{BET}$ = 982 m²/g, $V_P$ = 1.89 cm³/g, and with water $S_{BET}$ = 984 m²/g, $V_P$ = 2.03 cm³/g); (c) LiChrolut EN (copolymers of styrene and divinylbenzene, Merck) (initial $S_{BET}$ = 1512 m²/g, $V_P$ = 0.82 cm³/g, 2 h frozen (by liquid nitrogen) with acetone $S_{BET}$ = 1479 m²/g, $V_P$ = 0.81 cm³/g, and with water $S_{BET}$ = 1521 m²/g, $V_P$ = 0.82 cm³/g) (MND SCV/SCR method).

**Figure S13.** Pore size distributions of activated carbons (AC) prepared from (a) plum stones, PS, (PSO MASKPOL, Poland) (initial $S_{BET}$ = 1873 m²/g, $V_p$ = 1.04 cm³/g, after suspending/freezing $S_{BET}$ = 2233 m²/g, $V_p$ = 1.26 cm³/g) and (b) activated carbon NPK (carbonized coconut shells, CS) (Gryfskand Hajnówka, Poland) (initial $S_{BET}$ = 2165 m²/g, $V_p$ = 1.044 cm³/g, after suspending/freezing $S_{BET}$ = 2295 m²/g, $V_p$ = 1.118 cm³/g) (MND SCV/SCR method).

A certain enhancement of the porosity upon water freezing is observed for activated carbons (Figure S13). However, these textural effects do not practically change the surface chemistry of the materials. For example, infrared spectra treated polymers are very similar to those of the initial ones (Figure S19). Thus, the textural/morphological effects depend on the types of adsorbents (chemical structure, cross-linking degree, character of interactions between adsorbent and adsorbate) and adsorbate type (e.g., structural changes in water upon freezing with increasing sizes of ice crystallites causing huge disjoining pressure up to 2000 atm). This ice pressure can lead to destroying of core–shell nanoparticles (50–200 nm) of complex FMO, but smaller silica NPNP keep integrity under high pressure by ice crystallites in thick-wall stainless steel cryoreactors [4].

The adsorption potential ($U_0$) and free energy changes ($\Delta G$) upon water adsorption onto the silica surface from the air can be calculated using the Langmuir equation [1,2,8]:

$$\Theta = \frac{bC}{1+bC}, \tag{S9}$$

where $b = \gamma_L e^{-\frac{\Delta G}{R_g T}}$ or $K_L e^{\frac{U_0}{R_g T}}$, $\gamma_L$ and $K_L$ are the constants, using the minimization of the discrepancy functional. Instead of the average values determined with Equation (S9), the distribution functions of the energetic parameters can be calculated using Fredholm integral equation of the first kind with the kernel $\Theta_l$ similar to the right term in Equation (S9):

$$\Theta(T,p) = \int_{x_{\min}}^{x_{\max}} \Theta_l(T,p,x) f(x) dx, \tag{S10}$$

where *f(x)* is the unknown distribution function of a given parameter *x*. To calculate the *f(x)* function, the regularization method can be used, as the solution of Equation (S10) is a well-known ill-posed problem due to a strong influence of noise components on experimental data, which do not allow one to effectively utilize exact inversion formulas or iterative algorithms [14]. Equation (S9) was modified to describe clustered adsorption of water [8]:

$$\frac{a}{a_m} = \frac{bx\left[1+\sum_{i=2}^{m} i\varepsilon_i (bx)^{i-1}\right]}{1+bx\left[1+\sum_{i=2}^{m} \varepsilon_i (bx)^{i-1}\right]}, \tag{S11}$$

where $a_m$ is the monolayer capacity, $b = z_0 q_1/(q_0 q_a)$, $z_0=\exp(\mu_0/R_g T)$ is the absolute activity, $q_i$ is the statistical sum of the adsorption complex with *i* molecules, $\varepsilon_i = \exp(-i(\Delta E_i - \Delta E_0)/R_g T)$, $\Delta E_i$ is the energy of adsorption of the *i-th* molecule, $x = p/p_s$, and *m* is the molecule number at the adsorption site.

Equation (S11) was transformed into the integral equation [8]:

$$\frac{a}{a_m} = \int_{E_{\min}}^{E_{\max}} f(E) \frac{bx\left[1+\sum_{i=2}^{m} i\exp(-E/R_g T)(bx)^{i-1}\right]}{1+bx\left[1+\sum_{i=2}^{m} \exp(-E/R_g T)(bx)^{i-1}\right]} dE, \tag{S12}$$

where *f*(*E*) is the distribution function of the adsorption energy of water molecules forming clusters at the adsorption sites (normalized to one hydrogen bond per one molecule).

Silanols groups, single ≡SiOH and twin =Si(OH)$_2$ at $C_{SiOH}/C_{Si(OH)2}$ = 0.85/0.15–0.6/0.4 depending on the nanosilica type and its heating and other treatment histories [8], are the main adsorption sites for water (as well as for other adsorbates) at a silica surface [5,6]. Many of physicochemical properties of nanosilicas (and the interfacial phenomena at a surface) depend strongly not only on the primary particle and aggregate size distributions but also on the concentration of adsorbed water in the form of bound intact molecules ($C_w$) and ≡SiOH and =Si(OH)$_2$ groups, i.e., dissociatively adsorbed water ($C_{OH}$, Figures S14 and S15) and treatment history of the materials.

The adsorption of water at a surface of porous and nonporous nanoparticles or microparticles is a complex process. Besides molecular adsorption of water, it is possible dissociative adsorption of the molecules onto strained siloxane bonds at the outer and inner surfaces of the particles. Additionally, water molecules can penetrate into the volume of particles that leads to their swelling. This process is very slow and can occur

for 3–10 days, depending on the size of particles and their heating history. The particle swelling results in the diminution of the specific surface area of the materials. These processes are characterized by very different heat of adsorption (*Q*) and activation energy ($E_a$). For strongly dehydrated surfaces, the initial *Q* values can be greater than 200 kJ/mol. For hydrated surfaces, the *Q* values are close to the water condensation heat (45 kJ/mol). The $E_a$ values can be close to zero during adsorption onto the outer surface of particles or $E_a > 100$ kJ/mol on dissociative adsorption of the water molecules at different sites at the outer surface. The reactions in the inner volume of silica particles can be higher due to greater steric effects and hindered diffusion there (vide infra).

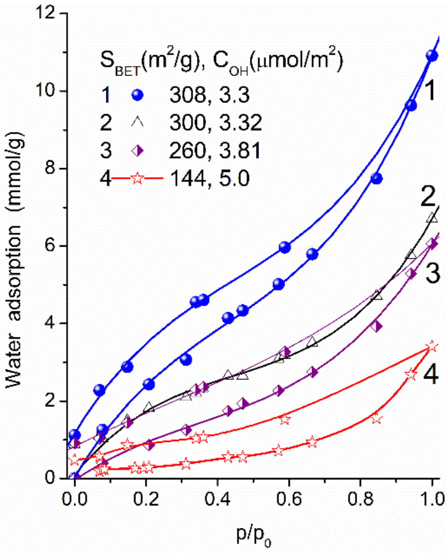


**Figure S14.** Adsorption–desorption isotherms of water onto different fumed silicas at 293 K.

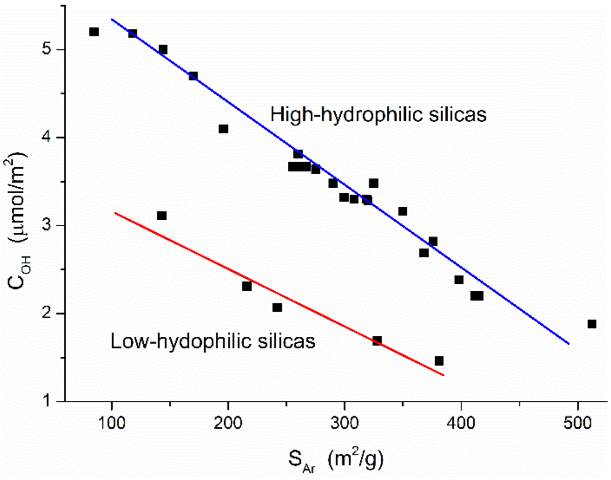


**Figure S15**. Relationship between the specific surface area $S_{Ar}$ and the concentration of free silanols at $\nu_{OH} = 3750$ cm$^{-1}$ for different nanosilicas.

Typically, water tends to be adsorbed in clustered structures, description, of which needs a special approach described by Eqs. (S11) and (S12). These equations give better results than the Langmuir equation for monolayer adsorption (Figure S16).

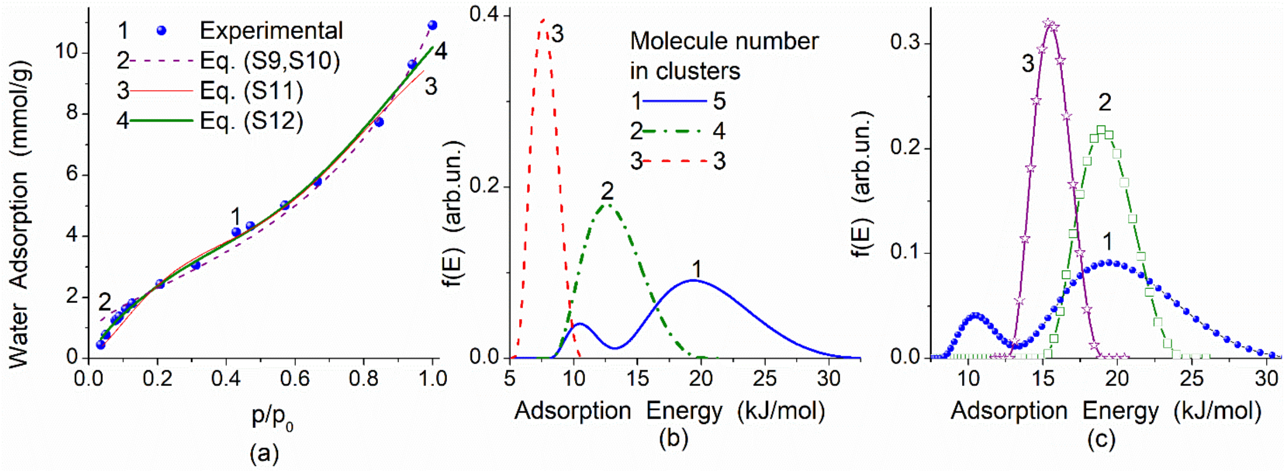


**Figure S16**. (a) Experimental adsorption isotherm (293 K) of water adsorbed onto A-300 (308 m$^2$/g) (curve 1) and fitting with standard Langmuir equation Eqs. (S9)–(S10) (curve 2), and equations for clustered adsorption of water with Equation (S11) (curve 3) and Equation (S12) (curve 4) at $m = 5$; (b) for the same A-300, water adsorption energy per the hydrogen bond per molecule, $f(E)$, with Equation (S12) and $m = 5$, 4, and 3; (c) $f(E)$ with Equation (S12) at $m = 5$ for A-300 (curve 1 (308 m$^2$/g, 3.30 µmol/m$^2$); curve 2 (300 m$^2$/g, $C_{OH} = 3.32$ µmol/m$^2$) and A-150 (curve 3, 144 m$^2$/g, 5.0 µmol/m$^2$) [8].

Thus, the use of the adsorption methods to characterize the textural features of adsorbents has not only certain advantages but also several disadvantages and systematic errors caused by (i) the effects of adsorbate (fluid) on the adsorbent texture (e.g., due to swelling or freezing); (ii) dependences of an occupied area and shape of a probe molecule on pore size and shape, as well on pressure; (iii) partially non-equilibrium

conditions; (iv) partial accessibility of long, curved, narrow pores for adsorbate molecules; (v) as well model and equation errors. Therefore, parallel measurements using various methods to analyze the textural and morphological characteristics are very desirable. Among these methods, the most appropriate and reliable results could be provided by SAXS and high-resolution TEM (HRTEM) methods. However, additional mathematic treatments of the corresponding data should be done.

**S2. Infrared spectroscopy**

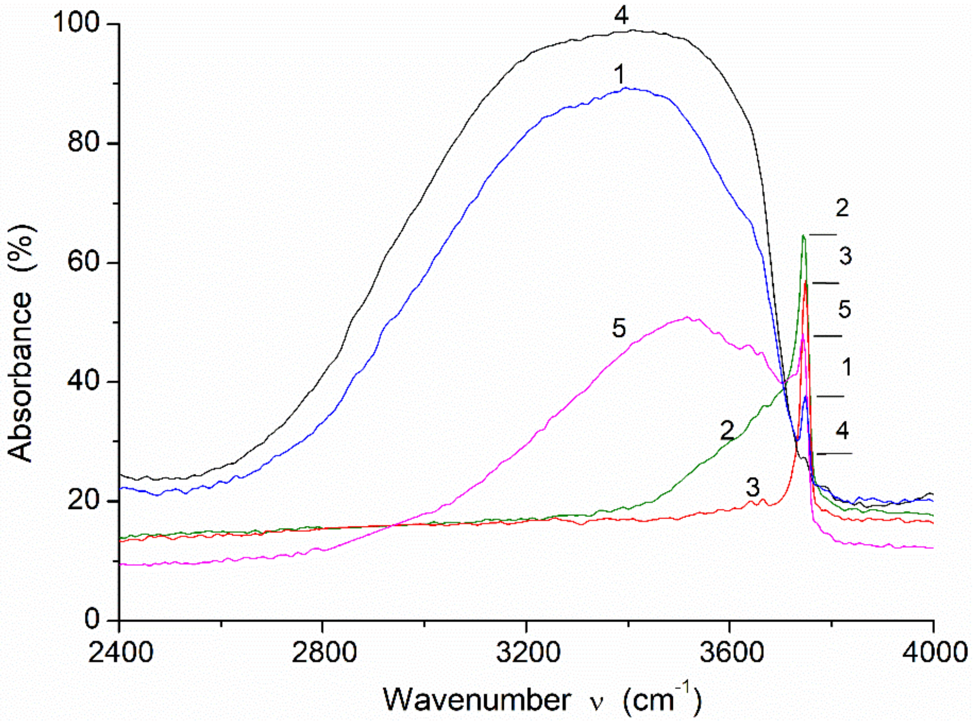


**Figure S17.** IR spectra of fumed silica A-300 (308 m$^2$/g) in air, after degassing at (2) 450 °C and (3) 650 °C, (4) letting in saturated water vapor, (5) degassing at room temperature.

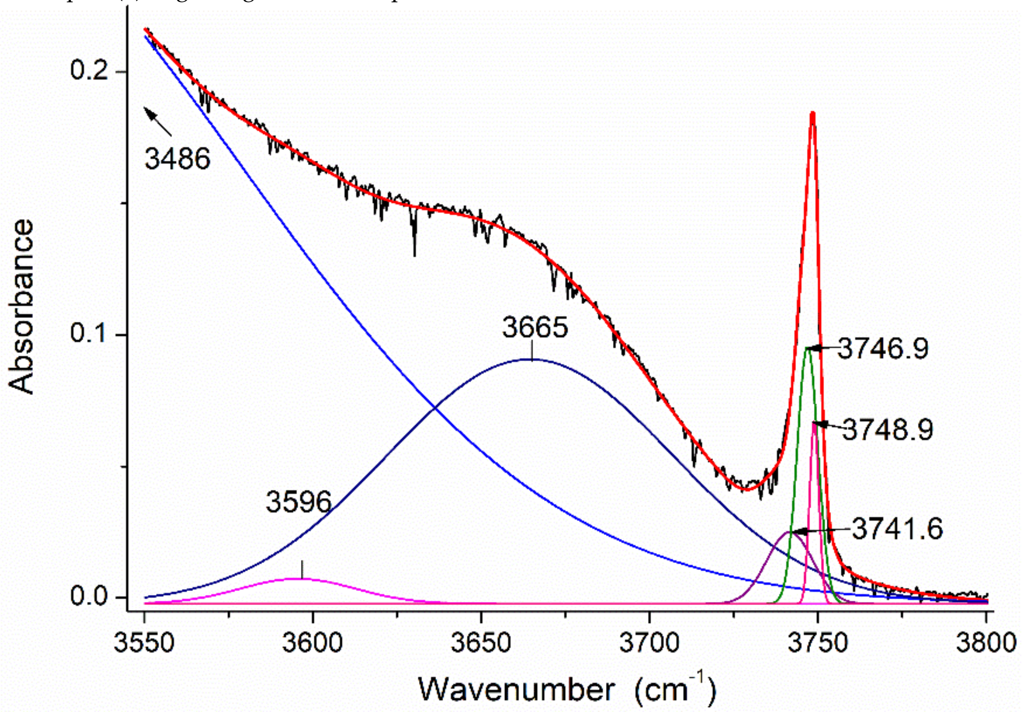


**Figure S18**. Decomposition of the IR spectrum of air-dried A-300 over the range of the OH-stretching vibrations.

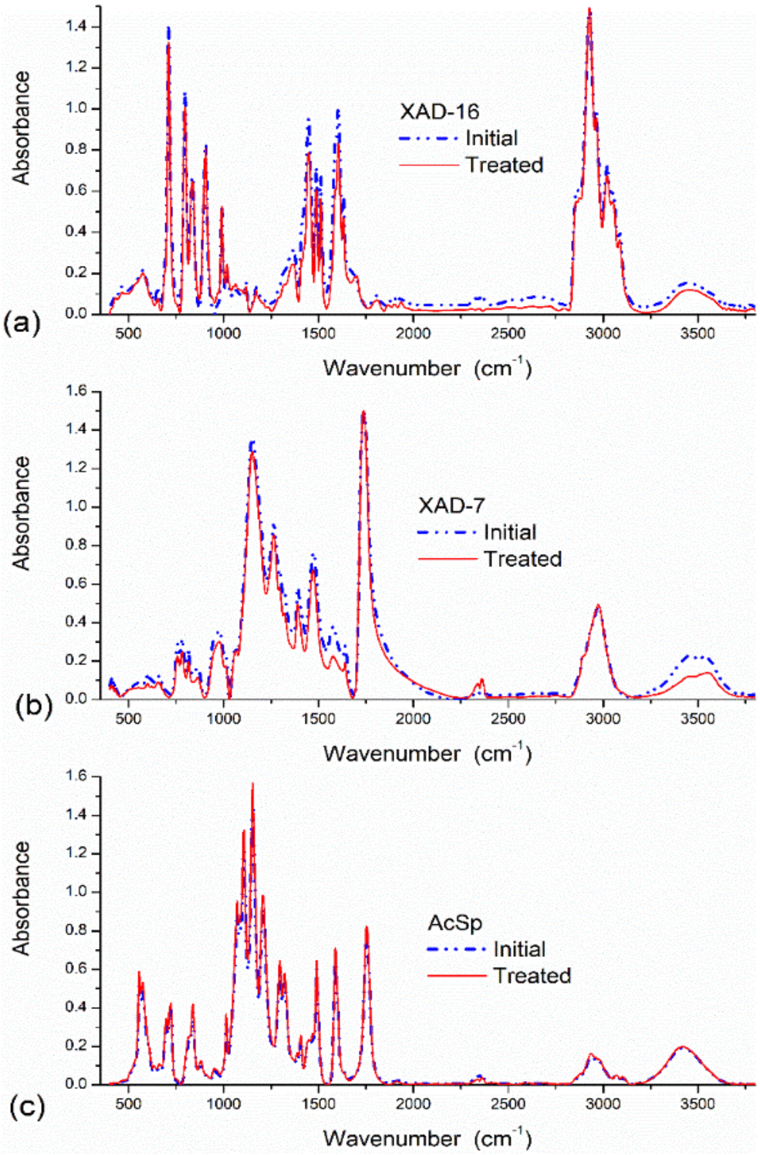


**Figure S19**. FTIR spectra of samples initial and wetted in acetone, frozen by liquid nitrogen and dried and characterized by strong textural changes: (a) XAD-16, (b) XAD-7, and (c) AcSp.

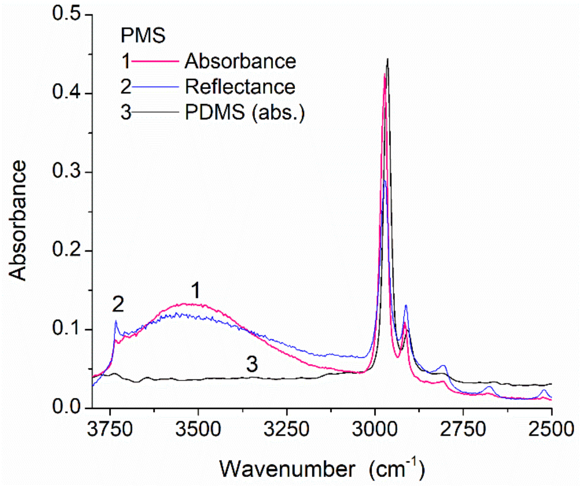


**Figure S20.** Infrared spectra of air-dried PMS (1, 2) and PDMS (3) in the range of 3800–2500 $cm^{-1}$ (pellets with pure polymer samples).

**S3. Small-angle X-ray scattering (SAXS)**

The differential PSD functions $f(r)$ based on the SAXS data could be calculated using the Fredholm integral equation of the first kind for scattering intensity $I(q)$ [17]:

$$I(q) = C\int_{r_{min}}^{r_{max}} \frac{(\sin qr - qr\cos qr)^2}{(qr)^2} V(r) f(r) dr, \quad \text{(S13)}$$

where $C$ is a constant, $q = 4\pi\sin(\theta)/\lambda$ the scattering vector value, $2\theta$ is the scattering angle, $\lambda$ is the wavelength of incident X-ray, $V(r)$ is the volume of a pore with radius $r$ (proportional to $r^3$), and $f(r)dr$ represents the probability of having pores with radius $r$ to $r + dr$. The values of $r_{min}$ (= $\pi/q_{max}$) and $r_{max}$ (= $\pi/q_{min}$) correspond to lower and upper limits of the resolvable real space due to instrument resolution. This equation could be solved using the CONTIN algorithm [14]. The $f(r)$ function could be converted into incremental PSD (IPSD) $\Phi(r_i) = (f(r_{i+1}) + f_V(r_i))(r_{i+1} - r_i)/2$ for better view of the PSD at larger $r$ values (similar to the PSD based on the adsorption data). The main advantage of the SAXS method upon the textural characterization is that all open and closed pores could be analyzed in contrast to the adsorption methods giving the characteristics only of pores accessible for probe molecules (Figures S7–S16); therefore, practically always, $S_{SAXS} > S_{BET}$ (Figures S21–S25). Comparison of the SAXS and adsorption PSD, as well the $S_{SAXS}$ and $S_{BET}$ values, allows one a deeper insight into the textural features of the materials studied under various conditions. Additionally, the SAXS data could be used to compute the PaSD for particles of different shapes [17–20].

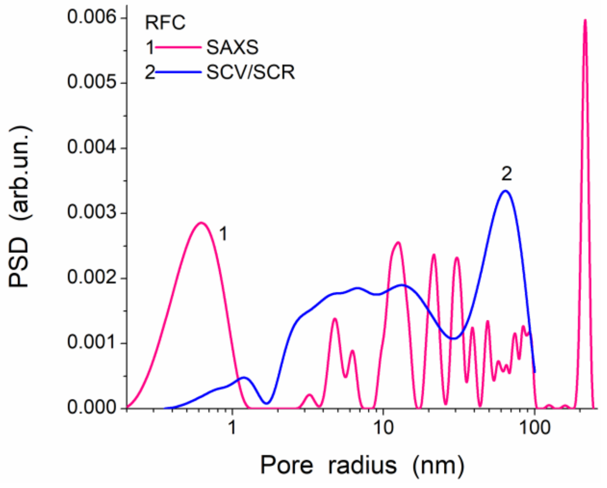


**Figure S21**. Pore size distributions of a char/bentonite (20/80 *w/w*) composite ($S_{BET}$ = 122 m²/g and $S_{SAXS}$ = 262 m²/g), prepared upon carbonization of resorcinol–formaldehyde resin added to bentonite, calculated using SAXS and SCV/SCR methods.

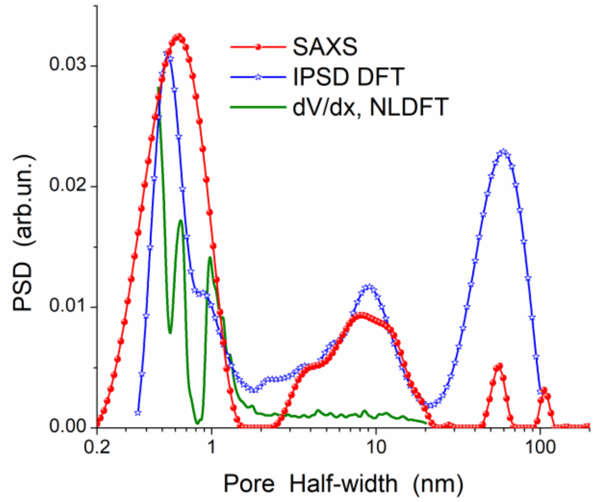


**Figure S22.** Pore size distributions of activated carbon (carbonization of phenol–formaldehyde resin and subsequent activation by $CO_2$ at 1183 K with 60% burn-off) ($S_{BET}$ = 1999 m²/g and $S_{SAXS}$ = 2211 m²/g) calculated using SAXS, DFT (Equation (S7)) and NLDFT methods.

To calculate the particle size distribution (PaSD) functions based on the SAXS data, several models of particles (e.g., spherical, cylindrical, lamellar ones and various blends of them [19,20]) could be used. For spherical particles, an integral equation similar to Equation (S13) could be written as follows:

$$I(q) = C\int_{R_{min}}^{R_{max}} P(q,r) f(r) dr, \quad \text{(S14)}$$

where $C$ is a constant, $r$ is the radius of particles, $f(r)$ is the distribution function (differential PaSD), and $P(r)$ is the form factor for spherical particles (the kernel of the integral equation): $P(q,r) = (4\pi r^3/3)^2 [\Phi(q)]^2$ where $\Phi(q,r) = (3/(qr)^3)[\sin(qr) - qr\cos(qr)]$. The PaSD concerning the volume of particles (as abundance in vol %) could be calculated as follows:

$$\text{abundance(vol\%)} = r^3 f(r) / \int r^3 f(r) dr. \quad \text{(S15)}$$

For cylindrical particles, there are two variable parameters, such as the radius ($r$) and length ($H$) of cylinders:

$$I(q) = C\int_{H_{min}}^{H_{max}} \int_{r_{min}}^{r_{max}} f(H) f(r) P(q,H,r) dH dr, \quad \text{(S16)}$$

where $P(q,H,r) = CV\int_0^{\pi/2} \frac{2J_1(qr\sin\theta)}{qr\sin\theta}\frac{\sin(0.5qH\cos\theta)}{0.5qH\cos\theta}\sin\theta d\theta$, $J_1(x)$ is the first-order Bessel function, $V = \pi r^2 H$ is the cylinder volume, and $C$ is a constant.

For lamellar particles

$$I(q) = C\int_{L_{\min}}^{L_{\max}} P(q,L)f(L)dL, \quad \text{(S17)}$$

where $L$ is the lamellar thickness, and the prefactor ($1/q^2$) is the so-called Lorentz factor required to randomize the orientation of the lamellar particle and $P(q,L) = (L/q)^2\left[\frac{\sin(qL/2)}{(qL/2)}\right]^2$. In the case of complex systems, several models with various blends of spherical, cylindrical and lamellar particles could be used with certain weight coefficients.

For a complex model of particles, the integral equation includes three terms:

$$\begin{aligned} I(q) = {} & c_{sph}\int_{r_{\min}}^{r_{\max}}\left(\frac{4\pi r^3}{3}\right)^2\left[\frac{3}{(qr)^3}(\sin(qr) - qr\cos(qr)\right]^2 f(r)dr + \\ & +c_{lam}\int_{L_{\min}}^{L_{\max}}(L/q)^2\left[\frac{\sin(qL/2)}{(qL/2)}\right]^2 f(L)dL + \\ & +c_{cyl}\int_{H_{\min}}^{H_{\max}} f(H)\int_{r_{\min}}^{r_{\max}}\frac{\pi r^2}{H}f(r)\int_0^{\pi/2}\sin\theta\frac{2J_1(qr\sin\theta)}{qr\sin\theta}\frac{\sin(0.5qH\cos\theta)}{0.5qH\cos\theta}d\theta dR dH \end{aligned} \quad \text{(S18)}$$

where $I(q)$ is the X-ray scattering intensity, $q = 4\pi\sin(\theta)/\lambda$ is the scattering vector value, $\theta$ is the scattering angle, $\lambda$ is the wavelength of incident X-ray, $R$ is the radius of particles, $H$ and $R$ are the length and radius of cylinders, $L$ is the lamellar thickness, $f(r)$, $f(L)$, and $f(H)$ are the distribution functions, $J_1(x)$ is the first-order Bessel function, $c_x$ are the weight coefficients calculated, as well $f(x)$ functions, using a self-consistent regularization procedure [9,21] to solve Equation (S18). For example, Figure S23 shows changes in the PaSD function (calculated with Equation (S18) using the SCR procedure) upon carbonization of resorcinol–formaldehyde resin bound to bentonite particles. These changes show that particle (polymer phase) carbonization results not only in diminution of the particles (e.g., at r < 7 nm) but also in certain consolidation of them (at $r$ = 8–15 nm and $r$ > 30 nm).

Comparison of the PaSD computed using SAXS and adsorption data (e.g., Figure S23) is useful for a deeper analysis of the particulate morphology of various adsorbents. The chord size distribution, $G(h)$ as a geometrical statistic description of a multiphase medium, could be calculated from the SAXS data [17,22]:

$$G(h) = C\int_0^{\infty}\left[K - q^4 I(q)\right]\frac{d^2}{dh^2}\left(-4\frac{\sin qh}{qh}\right)dq, \quad \text{(S19)}$$

where $K$ is the Porod constant (corresponding to scattering intensity $I(q) \sim Kq^{-4}$ in the Porod range).

The specific surface area from the SAXS data could be calculated (in $m^2/g$) using equation

$$S_{\text{SAXS}} = 10^4\pi\phi(1-\phi)\frac{K}{Q\rho_a}, \quad \text{(S20)}$$

where $\phi = \rho_a/\rho_0$ is the solid fraction of adsorbent, and $Q$ is the invariant

$$Q = \int_0^{\infty} q^2 I(q)dq. \quad \text{(S21)}$$

The $Q$ value is sensitive to the range used on the integration of Equation (S21) (since experimental $q$ values are measured between the $q_{\min}$ and $q_{\max}$ values different from 0 and $\infty$). Therefore, the invariant value $Q$ could be calculated using equation

$$Q = \sum_{q_{\min}}^{q_{\max}}(I(q_i) - b)q_i^2\Delta q_i + K/q_{\max} \quad \text{(S22)}$$

where $b$ is a constant determined using equation

$$I(q)q^4 = K + bq^4 \quad \text{(S23)}$$

valid in the Porod range.

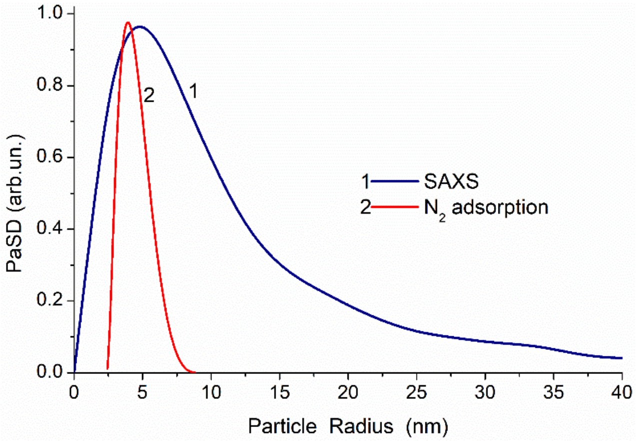


**Figure S23**. PaSD based on small-angle X-ray scattering (SAXS) (model of spherical particles, Equation (S16)) and nitrogen adsorption data (model with voids between spherical nanoparticles, Equation (S7)) for fumed silica A-300.

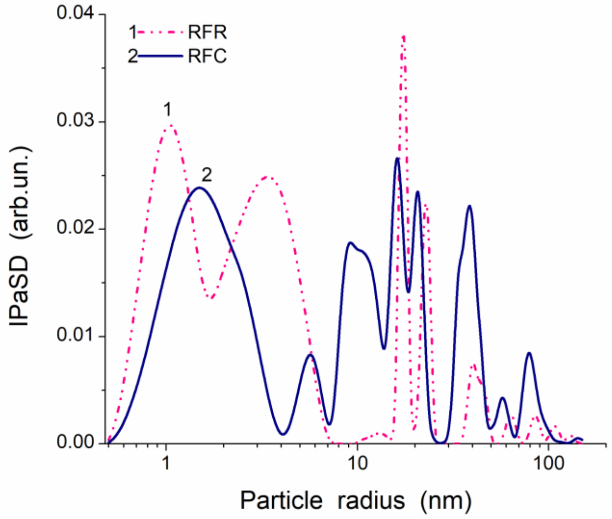


**Figure S24.** Particle size distributions of resorcinol–formaldehyde resin/bentonite ($S_{BET}$ = 160 m$^2$/g and $S_{SAXS}$ = 194 m$^2$/g) and char/bentonite (20/80 *w*/*w*) composite ($S_{BET}$ = 122 m$^2$/g and $S_{SAXS}$ = 211 m$^2$/g), prepared upon carbonization of resorcinol–formaldehyde resin added to bentonite, calculated using SAXS/SCR method with a complex model with spherical, cylindrical and lamellar particles (Equation (S18)).

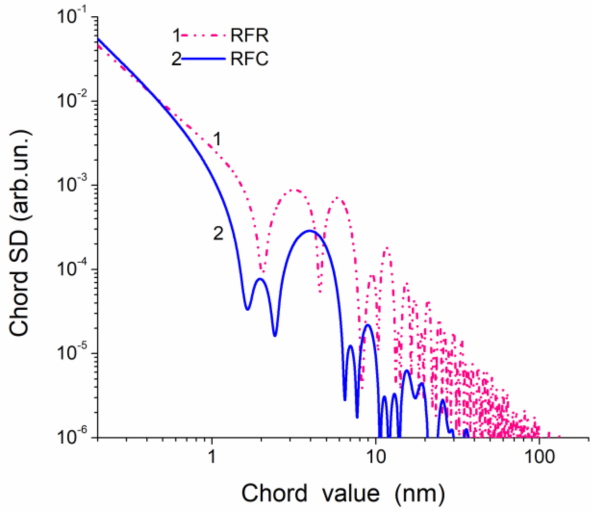


**Figure S25**. Chord size *G*(*h*) distributions of resorcinol–formaldehyde resin/bentonite ($S_{BET}$ = 160 m$^2$/g and $S_{SAXS}$ = 194 m$^2$/g) and char/bentonite (20/80 *w*/*w*) composite ($S_{BET}$ = 122 m$^2$/g and $S_{SAXS}$ = 211 m$^2$/g), prepared upon carbonization of resorcinol–formaldehyde resin added to bentonite, calculated using SAXS method with Equation (S19).

The *G*(*h*) curves (Figure S25) show that carbonization of resorcinol–formaldehyde resin bound to bentonite particles results in a diminution of the thickness of pore walls over the total range. The carbonization results in a decrease in the value of $S_{BET}$ (160 m$^2$/g → 122 m$^2$/g) due to consolidation of particles that causes an increase in a part of closed pores since the $S_{SAXS}$ value increases (194 m$^2$/g → 211 m$^2$/g) during the carbonization (Figure S25). Thus, the SAXS method gives useful information on global textural changes in the materials that significantly complete the adsorption data.

## S4. High-Resolution Transition Electron Microscopy (HRTEM), Scanning Electron Microscopy (SEM) and Atom Force Microscopy (AFM)

TEM and SEM images (Figure S26) could be used to calculate both PSD and PaSD functions (Figure S27). However, this needs certain methods (computer programs) for image treatment using, e.g., such software as ImageJ [23] (with a granulometry plugin), Fiji [24] (with a local thickness plugin) or others [25].

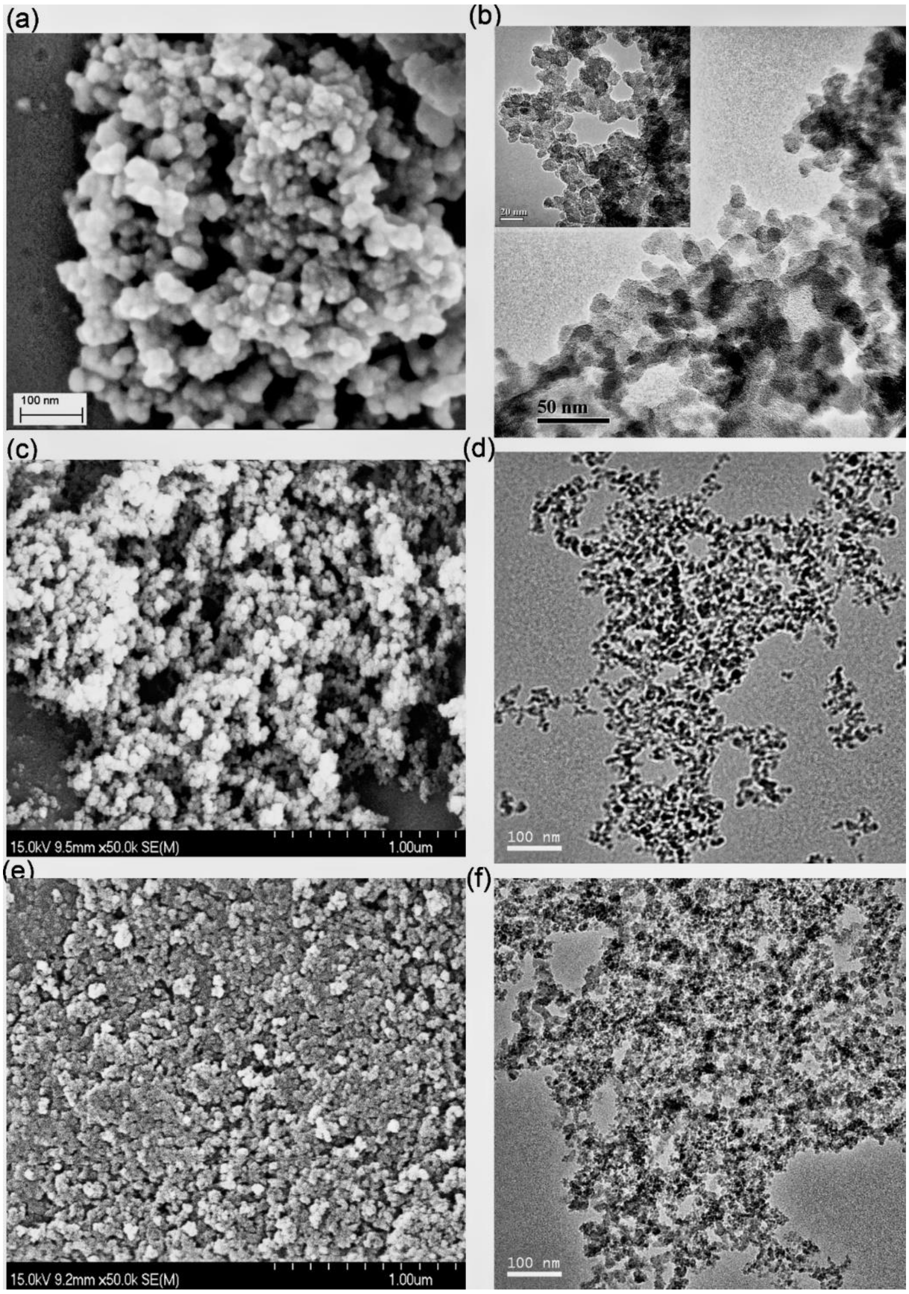


**Figure S26**. (a,c,e) SEM and (b,d,f) TEM images of (a–d) fresh A-300 and (e,f) A-500 (11 year-aged) showing nonporous nanoparticles (NPNP) aggregates and agglomerates of aggregates.

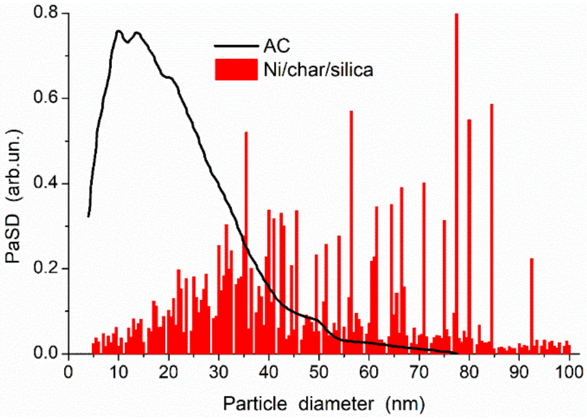


**Figure S27**. Particle size distributions (AFM images treated with Fiji, local thickness plugin) of activated carbon, AC ($S_{BET}$ = 2019 m²/g, MAST Carbon Technology Ltd.), and char prepared using potato starch mixed with $Ni(NO_3)_2$ and Sipernat 50 (precipitated silica, $S_{BET}$ = 503 m²/g, average particle size of 50 μm, Evonik) and carbonized at 500 °C for 3 h (in char, 11.5 wt % of carbon and 12.7 wt % of Ni).

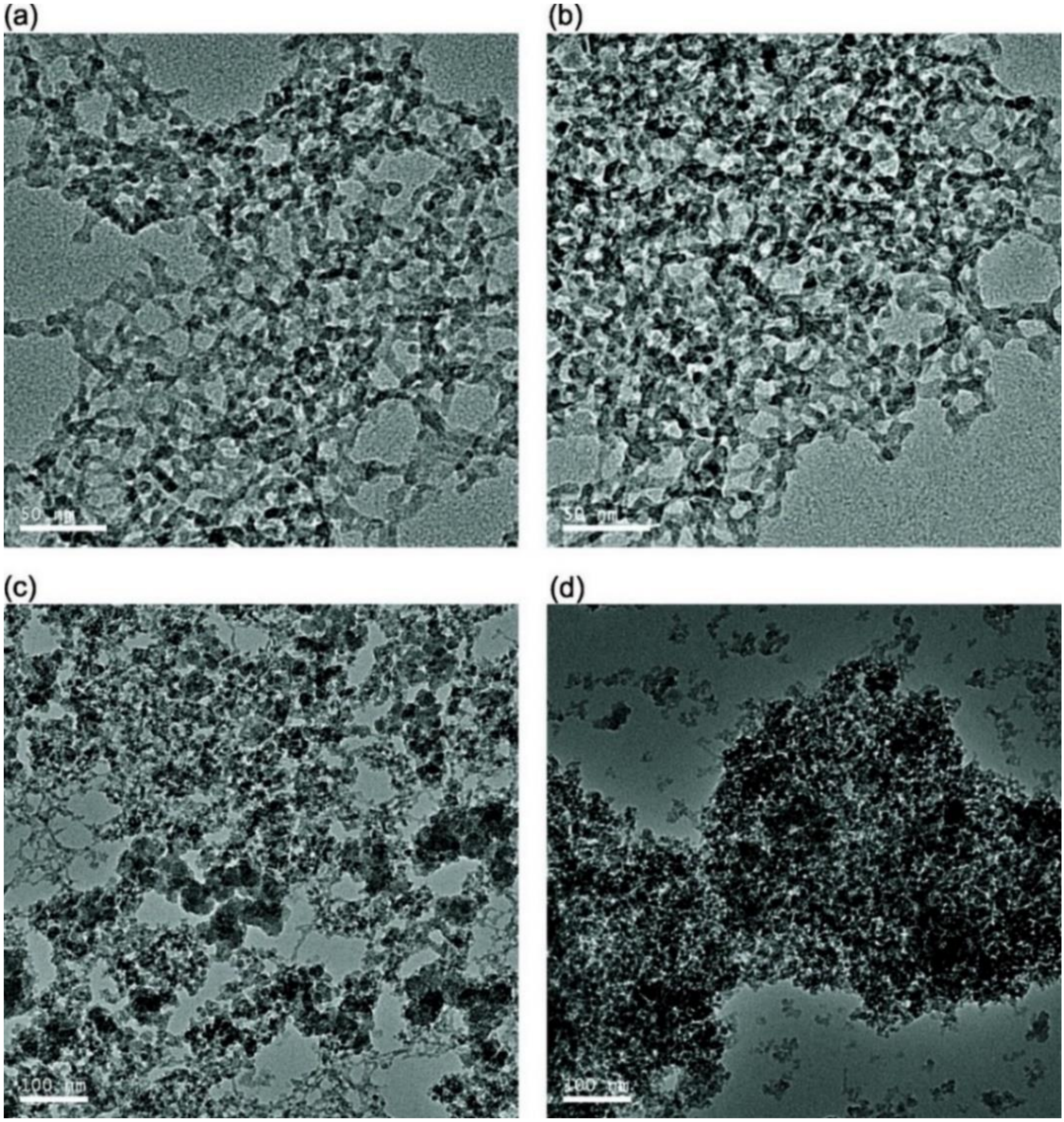


**Figure S28**. TEM images of degassed (a) initial PMS (Enterosgel); (b) PMS dried at room temperature and stirred; (c) PMS/A-300 (1:1); (d) cPMS/A-300 (1:1).

The quantitative analyses of microscopic images have certain advantages and disadvantages described in detail elsewhere [25]. The advantages are due to practically direct quantitative information on the particulate morphology and texture of the materials studied that can be compared with the results based on indirect information based, e.g., on the adsorption and SAXS data. The disadvantages of the approaches are due to certain problems of quantitative analysis of images, especially pore walls and pore sizes, with a certain ambiguity of their representation on grayscale images [25]. Figure S26 shows images of fresh A-300 and aged (about 11 years) A-500. The latter lost the surface area from 492 $m^2/g$ (fresh) to 331 $m^2/g$ (aged). This effect is caused by enhanced aggregation of NPNP (comp. Figures S26a–d and S26e,f) that results in significant changes in the nitrogen adsorption–desorption isotherm (Figure S4a) and PSD (Figure S4b). These textural changes over the total range of pores (voids between NPNP) are similar (but slightly different) to those observed after the mechanical compaction of the FMO powders described above. Thus, FMO based on amorphous silica or other amorphous silicas (which are a hard-moving liquid), e.g., silica gels, are aged-time-sensitive materials. This time-dependent aggregation effect is of importance from a practical point of view.

The TEM/SEM/AFM image treatments in parallel with adsorption, SAXS and spectroscopic data give useful information for complex systems with carbons, polymers, nanooxides, etc. (Figures S2–S31, Table S1).

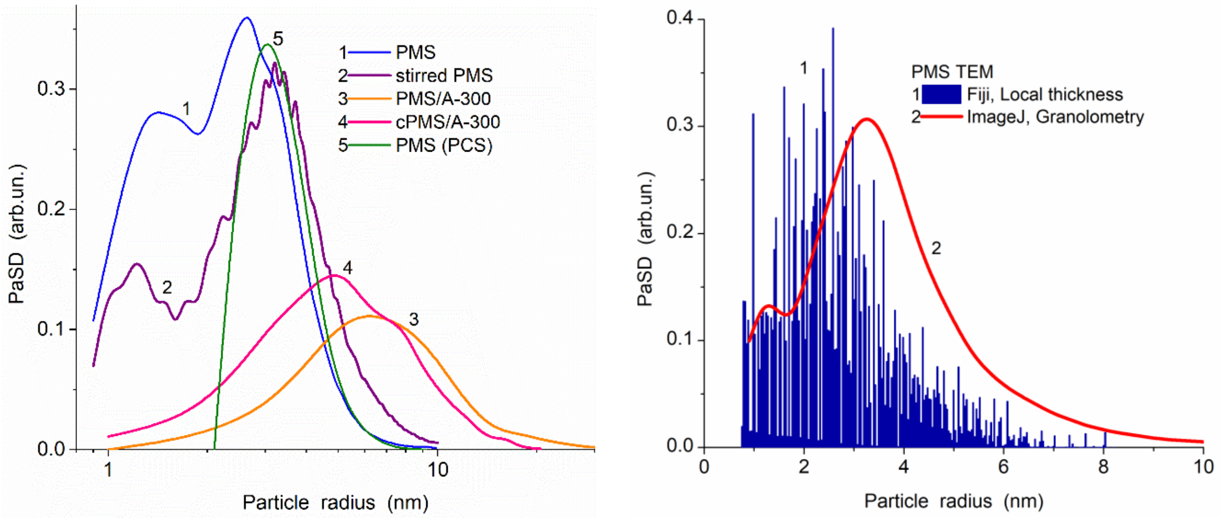


**Figure S29.** (a) Nanoparticle (i.e., with no secondary structures) size distributions (PaSD, curves 1–4) calculated from TEM images shown in Figure S28 (using ImageJ with granulometry plugin, https://imagej.nih.gov/ij/download.html), and (curve 5) PaSD for aqueous suspension of Enterosgel (0.0078 wt %, pH 3.1) measured using photon correlation spectroscopy (Zetasizer 3000, Malvern Ins., λ = 633 nm, scattering angle 90°). (b) Comparison of the PaSD of PMS calculated with Fiji/local thickness plugin and ImageJ/Granulometry plugin.

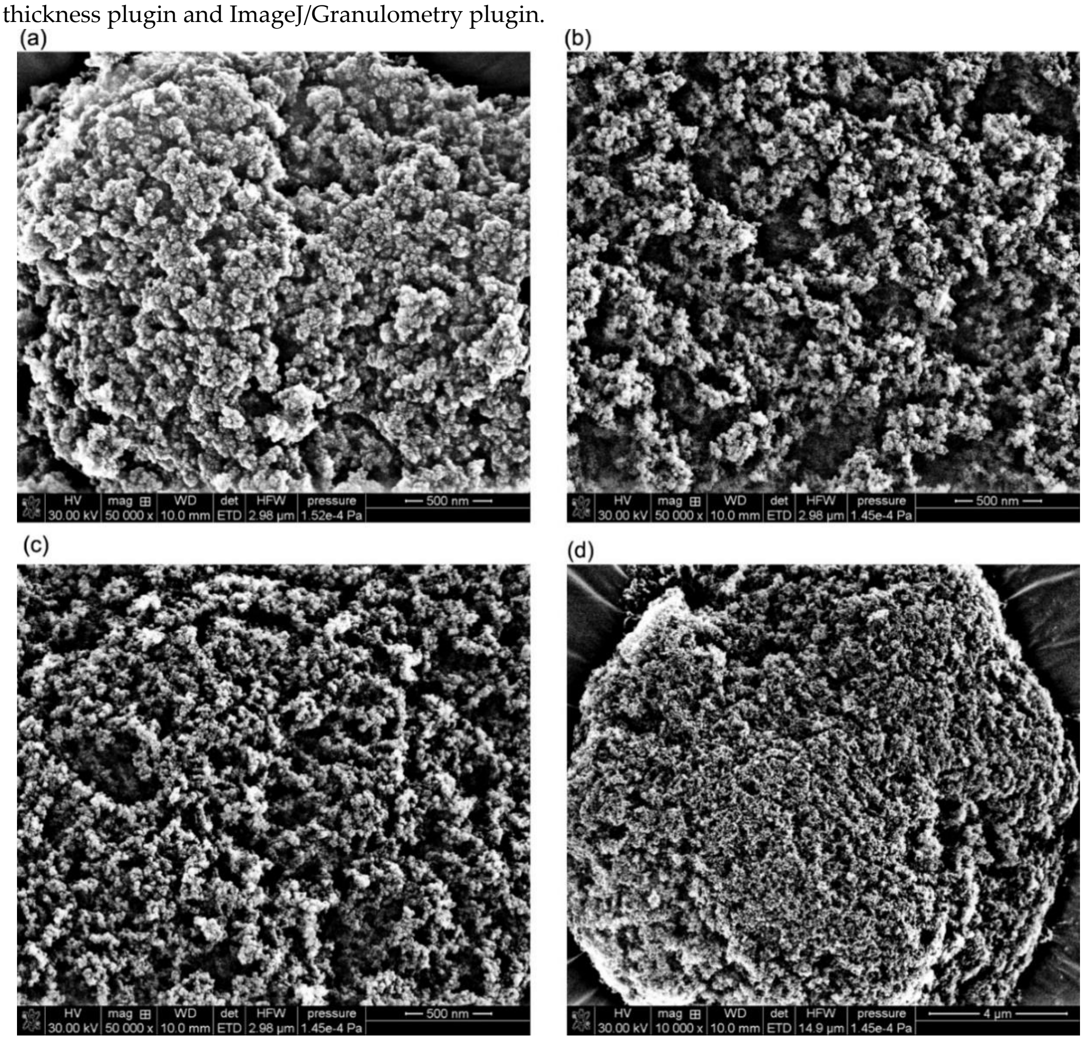


**Figure S30.** SEM images of (a) A-300/PDMS200/dimethyl carbonate (DMC), (b) A-300/PDMS1000/DMC, (c,d) A-300/PDMS12500/DMC at different magnifications with scale bar of (a–c) 500 nm and (d) 4 µ.

Nanosilica A-300 (Pilot plant of Chuiko Institute of Surface Chemistry, Kalush, Ukraine) and commercial polydimethylsiloxanes PDMS200 (viscosity of ca. 200 $mm^2/s$), PDMS1000 (viscosity of ca. 1000 $mm^2/s$) ("Kremniypolymer", Zaporozhye, Ukraine), and PDMS12500 (Wacker® AK 12,500 silicone fluid, purity > 99%, viscosity of ca. 12,500 $mm^2/s$ at 25 °C, density of ca. 0.97 $g/cm^3$) were used for silica modification and preparation of composites [4,8,21]. A series of modified silicas with the same content (16.7 wt %) of chemically bound different PDMS was synthesized (in a glass reactor, volume 1 $dm^3$, with a mixer, 500 rpm) with the presence of dimethyl carbonate (DMC, purity > 99.0 wt %, as a siloxane bond-breaking reagent, which does not contribute the weight of modified silica due to the reaction mechanism) in the gaseous dispersion media (i.e., without a solvent) at 100 °C or 200 °C (using 10 g A-300, 2 g PDMS, and 2 g DMC). Upon the second stage, PDMS12500 (dissolved in *n*-hexane, purity > 99.9%) was physically adsorbed (in the amount of 14–95 wt %) onto unmodified or PDMS/DMC-modified silicas. Before the PDMS adsorption, the samples were dried at 100 °C for 1 h, and then the *n*-hexane solution (3 wt %) of PDMS12500 was added, and the suspension was carefully stirred. It was dried at room temperature for 24 h and then at 100 °C for 3 h.

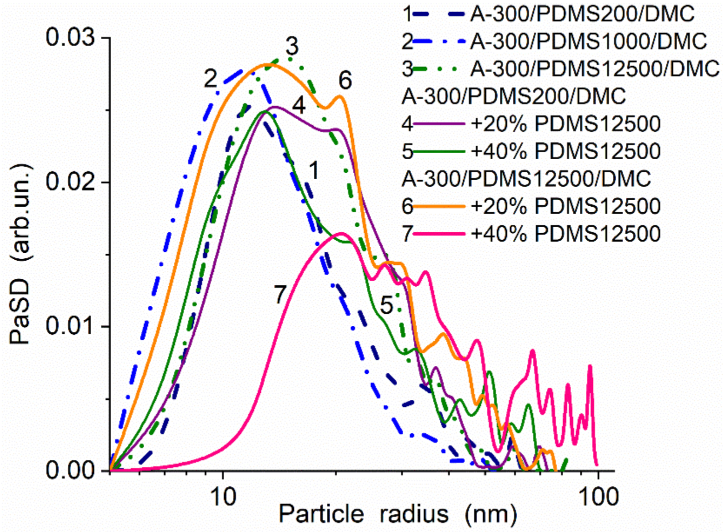


**Figure S31.** Particle size distributions of PDMS/DMC chemically modified A-300 and with additional physical adsorption of PDMS12500 calculated using SEM images of a maximal magnification (scale bar 500 nm) treated with ImageJ/granulometry plugin.

**S5. Quasi-elastic light scattering**

The characteristics of such highly disperse materials as FMO depend strongly on the dispersion media properties because of several factors: (i) surface charging, (ii) NPNP swelling, (iii) colloidal stability and aggregation instability and others [3–8,26–28]. Electrophoretic mobility and secondary particle size distribution (SPSD) investigations could be performed using such apparatus based on the quasi-elastic light scattering (QELS) (photon correlation spectroscopy, static light scattering, or dynamic light scattering) method as a Zetasizer 3000 (Malvern Instruments). Deionized distilled water and FMO samples (1–30 g per $dm^3$ of the water) could be utilized to prepare suspensions sonicated for several min using an ultrasonic disperser (e.g., Sonicator Misonix, power 500 W and frequency 22 kHz). The pH values could be adjusted by the addition of 0.1 M HCl or NaOH solutions, and the salinity could be changed by the addition of NaCl, $NaClO_4$, etc. The electrophoretic behavior and the SPSD concerning the intensity of scattered light ($SPDS_I$), volume, surface ($SPDS_S$), and particle number ($SPDS_N$) of FMO in the aqueous suspensions were described in detail elsewhere [26,27].

According to the Smoluchowski theory [28], there is a linear relationship between the electrophoretic mobility $U_e$ (QELS measured) and the $\zeta$ potential: $U_e = A\zeta$, where $A$ is a constant for a thin electrical double layer (EDL) at $\kappa a >> 1$ (where $a$ denotes the particle radius, and $\kappa$ is the Debye–Hückel parameter). For a thick EDL ($\kappa a < 1$), e.g., at pH close to the isoelectric point (IEP), the equation with the Henry correction factor is more appropriate $U_e = 2\varepsilon\zeta/(3\eta)$, where $\varepsilon$ is the dielectric permittivity; and $\eta$ is the viscosity of the liquid [28]. For FMO dispersions in the aqueous media, aggregation of NPNP is always characteristic [26]. However, sometimes the first peak of SPSD (Figure S32a, curve 4) corresponds to NPNP (Figure S32b). The hydrodynamic diameter of NPNP could be 5–20% larger than the geometric one due to the presence of an immobile layer in

EDL of liquid/bound counter ions depending on pH, IEP, salinity, etc. Other peaks in the SPSD correspond to NPNP aggregates and aggregates/agglomerates (Figure S32b). Note that the QELS PaSD peak of NPNP corresponds to smaller sizes than that estimated from TEM image and nitrogen adsorption data because there were different A-300 samples with 330 $m^2/g$ (QELS) and 294 $m^2/g$ (TEM, nitrogen adsorption). Thus, the QELS method could be used to estimate the aggregation of FMO particles in liquid dispersion media depending on the conditions of the measurements. This FMO behavior can affect the results of the NMR cryoporometry and relaxometry investigations of the suspensions.

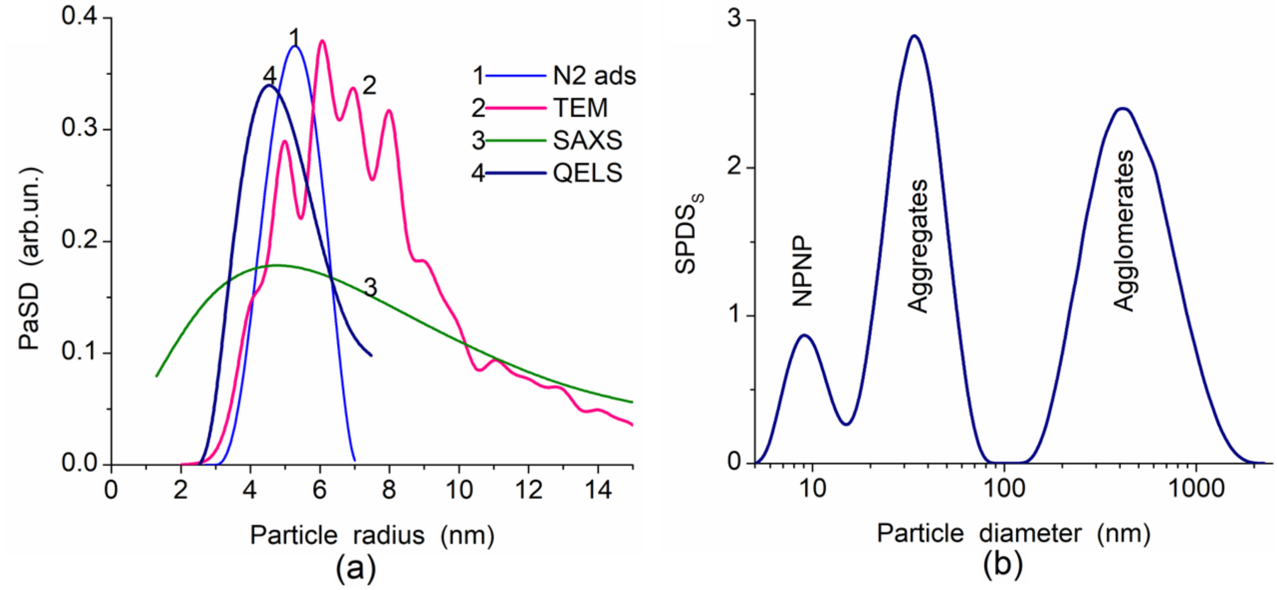


**Figure S32**. (a) Primary nanoparticle size distributions of A-300 ($S_{BET}$ = 294 $m^2/g$) estimated from the data of nitrogen adsorption (V/SCR method), HRTEM (ImageJ with granulometry plugin), SAXS (with a model of spherical particles), and QELS (a model of spherical particles); and (b) particle size distribution (SPSDs) in aqueous suspension of A-300 ($S_{BET}$ = 330 $m^2/g$, concentration of 3 wt %).

**S6. NMR cryoporometry**

The confined space effects play an important role for liquid adsorbates or dispersion media (e.g., water, etc.), especially at temperatures lower than the freezing temperature of the bulk liquid that can be utilized in various methods to estimate the textural characteristics on the basis of the temperature behavior of liquids confined in pores [8]. Water can be frozen in narrower pores (or voids between nanoparticles) at lower temperatures as described by the Gibbs–Thomson (GT) relation for the freezing point depression for liquids confined in cylindrical pores at radius $R$ [8,29,30]

$$\Delta T_m = T_{m,\infty} - T_m(R) = -\frac{2\sigma_{sl} T_{m,\infty}}{\Delta H_f \rho R} = \frac{k_{GT}}{R}, \qquad \text{(S24)}$$

where $T_m(R)$ is the melting temperature of the ice in cylindrical pores of radius $R$, $T_{m,\infty}$ the bulk melting temperature, $\Delta H_f$ the bulk enthalpy of fusion, $\rho$ the density of the solid, $\sigma_{sl}$ the energy of solid–liquid interaction and $k_{GT}$ is the Gibbs–Thomson constant ($k_{GT}$ = 40–90 K nm for various FMO, porous oxides (silica gels), polymers, carbons, etc.). Equation (S24) could be used to determine the cluster size distributions (CSD) ($f_V(R) = dV_{uw}(R)/dR$) of water unfrozen at $T < 273$ K [8] and bound to disperse or porous solids wetted or located in the aqueous suspensions.

Equation (S24) can be transformed into Equation (S25) for two (or more if their signals could be separated in total spectra deconvoluted) immiscible liquids (e.g., water and benzene characterized by different $^1$H NMR signals [8]) with consideration for their amounts in a mixture and their temperature-dependent signal intensity (obtained on deconvolution of the total signal)

$$\frac{dV_{u,i}}{dR} = \frac{A_i}{k_i}(T - T_{m,\infty,i})^2 \frac{dC_{u,i}}{dT}, \qquad \text{(S25)}$$

where $i$ denotes an adsorbate number, $C_{u,i}(T)$ is the integral intensity of a $\delta_H$ band for the $i$-th adsorbate as a function of temperature, and $A_i$ is a weight constant dependent on the molecular volume ($v_i$), the number of

protons ($n_i$) in a molecule of the $i$-th adsorbate and the used units. Equation (S25) can be transformed into integral GT (IGT) equation, replacing $dV/dR$ by $f(R)$, converting $dC/dT$ to $dC/dR$ and integrating by $R$,

$$C_{u,i}(T) = A\int_{R_{\min}}^{R_{\max}} \left(\frac{k_i}{(T_{m,\infty,i} - T_{m,i}(R))R}\right)^2 f_i(R)dR \cdot \qquad \text{(S26)}$$

CSD could be converted into incremental CSD (ICSD) $\Phi_V(R_i) = (f_V(R_{i+1}) + f_V(R_i))(R_{i+1} - R_i)/2$ at $\sum\Phi_{V,i}(R) = V_{uw}$. Integration of the $f_V(R)$ and $f_S(R)$ functions at $R < 1$ nm, 1 nm $< R <$ 25 nm, and 25 nm $< R <$ 100 nm gives the volume and specific surface area of nano-, meso- and macropores, respectively, filled by unfrozen liquid. The specific surface area ($S_{uw}$) of adsorbents in contact with bound water (assuming for simplicity that the density of unfrozen bound water $\rho_{uw}$ = 1 g/cm$^3$) can be determined from the amount of this water $C_{uw}^{\max}$ (estimating pore volume as $V_{uw} = C_{uw}^{\max}/\rho_{uw}$) at 273 K and pore size distribution $f(R)$ with a model of cylindrical pores [8]

$$S_{uw} = \frac{V_{uw}}{2R_{av}} = \frac{2C_{uw}^{\max}}{\rho_{uw}} \int_{R_{\min}}^{R_{\max}} f(R)dR \Big/ \int_{R_{\min}}^{R_{\max}} f(R)RdR, \qquad \text{(S27)}$$

where $R_{\min}$ and $R_{\max}$ are the minimal and maximal radii of pores filled by unfrozen water, respectively. In the case of calculations of the structural characteristics of nanopores ($R < 1$ nm), mesopores (1 nm $< R <$ 25 nm) and macropores ($R >$ 25 nm), the $R_{\min}$ and $R_{\max}$ values are the boundary $R$ values for the corresponding pore types (including $R_{\min}$ = 0.2 nm for nanopores). The $C_{uw}^{\max}/\rho_{uw}$ value should be replaced by the corresponding values of the volumes of nanopores, mesopores, or macropores [8]. The average melting temperature $<T_m>$ could be calculated using a formula related to the ratio of the first and zero moments of the distribution function

$$<T_m> = \int_{T_{\min}}^{T_0} TC_{uw}(T)dT \Big/ \int_{T_{\min}}^{T_0} C_{uw}(T)dT, \qquad \text{(S28)}$$

where $T_0$ = 273.15 K and $T_{\min}$ is the temperature corresponding to $C_{uw}$ = 0.

Figure S33 shows the $f(R)$ functions for the aqueous suspensions of A-300 at different concentrations of silica compared to the $N_2$ adsorption PSD (V/SCR). The value of $C_{A\text{-}300}$ = 12.3 wt % is close to that, causing complete structurization (clusterization) of the dispersion [8]. Therefore, changes in the $f(R)$ curves are non-monotonic with increasing $C_{A\text{-}300}$ value due to different structurization of the dispersion vs. A-300 concentration.

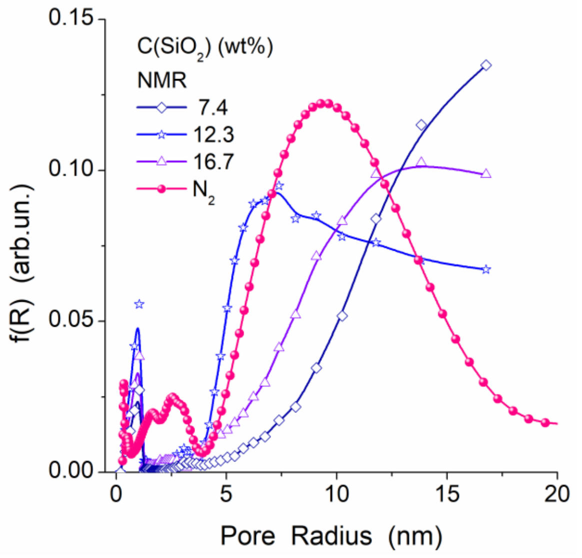


**Figure S33**. Size distributions of pores (nitrogen adsorption/desorption treated with V/SCR method) and clusters with unfrozen water (T < 273 K) filling voids between silica (A-300) NPNP in aggregates located in aqueous suspensions at different concentration of silica (Gibbs–Thomson (GT) method).

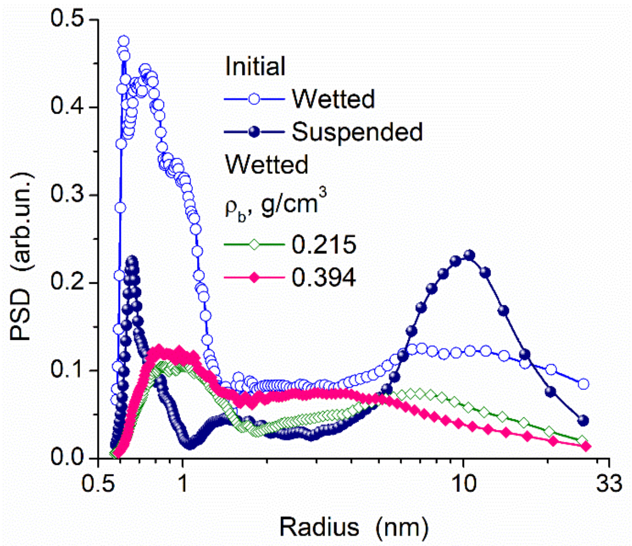


**Figure S34**. Differential functions concerning the sizes of unfrozen water structures for hydrated silica ($h$ = 3.0, 40.7, 1.2 and 1.0 g/g, respectively) with the specific surface area in contact with unfrozen water $S_{uw}$ = 322, 281, 123, 124 m$^2$/g, respectively.

The NMR cryoporometry gives useful information on the behavior of bound water, depending not only on the silica (water) content but also on the A-300 treatment (e.g., MCA) effects (Figure S34). A strong increase in the content of water from $h$ = 3 g/g (wetted powder) to 40.7 g/g (suspension) results in a relative decrease in the content of strongly bound water forming small clusters in narrow voids because, in the suspension, the average distance between NPNP can strongly increase (due to electrostatic repulsion interactions). The MCA compaction of A-300 results in a relative increase in the contribution of small clusters ($R$ < 2 nm) because the contribution of narrow voids increases (Figures S3 and S34). An enhancement in the MCA compaction resulting in a greater $\rho_b$ value (from 0.22 to 0.39 g/cm$^3$) slightly affects the distribution of unfrozen water clusters in wetted samples over the total size range (Figure S34) because the PSD changes too (Figure S3), but the water content is not enough to strongly change the NPNP aggregation that observed in the aqueous suspensions. Thus, NMR cryoporometry gives information not only on the textural characteristics of the materials being in different dispersion media but also on the temperature behavior of unfrozen liquids confined in different pores. Similar information could be obtained using the DSC thermoporometry method based on the Gibbs–Thomson relation for the melting point depression for liquids confined in pores.

**S7. DSC thermoporometry**

Melting DSC curves of bound water (ice) [8,31,32] can be used to calculate the pore sizes filled by this water as follows:

$$R_p(nm) = 0.68 - \frac{32.33}{T_m - T_{m0}}, \qquad \text{(S29)}$$

where $T_m$ and $T_{m0}$ are the melting temperatures of confined and bulk water, respectively. The pore size distribution $dV/dR$ can be calculated from the DSC melting curves of bound ice

$$\frac{dV}{dR}(cm^3 nm^{-1} g^{-1}) = \frac{\frac{dq}{dt}(T_m - T_{m0})^2}{32.33\rho\beta m\Delta H(T)}, \qquad \text{(S30)}$$

where $dq/dt$, ρ, β, $m$ and $\Delta H(T)$ are the DSC heat flow, the water density, the heating rate, the sample mass and the melting enthalpy of ice, respectively. The $\Delta H$ values as a function of temperature for water can be estimated as follows

$$\Delta H(T)(\mathrm{J\ g^{-1}}) = 332 + 11.39(T_m - T_{m0}) + 0.155(T_m - T_{m0})^2 \quad \text{(S31)}$$

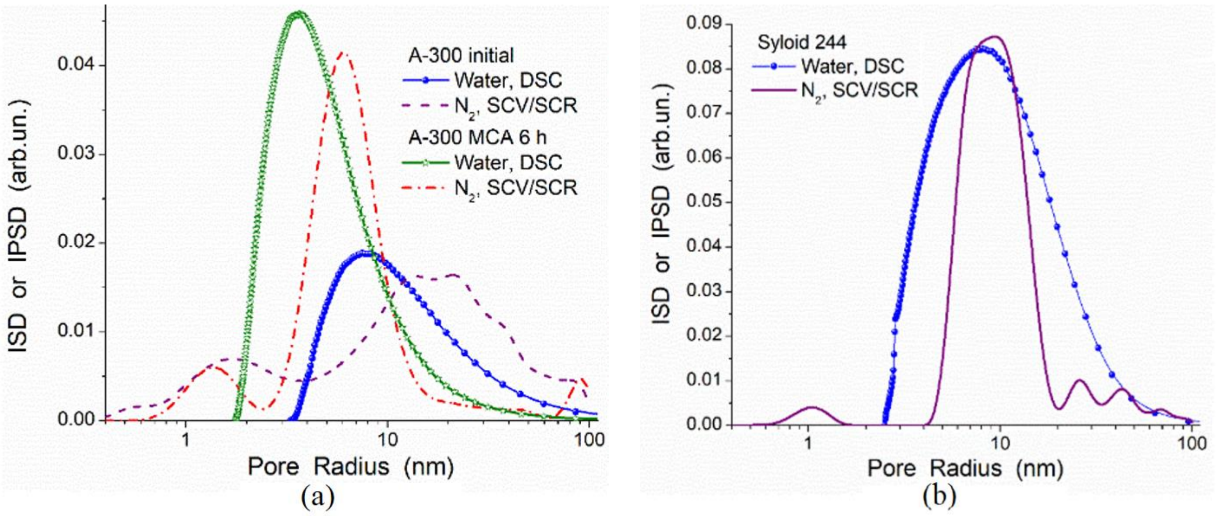


**Figure S35.** Comparison of the IPSD calculated from nitrogen adsorption–desorption isotherms and ISD based on the DSC melting thermograms of water (ice) bound to (a) initial A-300 ($S_{BET}$ = 331 m$^2$/g, $V_p$ = 0.826 cm$^3$/g), mechanochemical activation (MCA) 6 h A-300 ($S_{BET}$ = 331 m$^2$/g, $V_p$ = 0.771 cm$^3$/g), and (b) Syloid 244 ($S_{BET}$ = 336 m$^2$/g, $V_p$ = 1.591 cm$^3$/g.

The Gibbs-Thomson equation applied to DSC melting thermograms allows one to accurately calculate the PSD for silica gels (with the model of cylindrical pores since the errors of this model are relatively small for silica gels [8]) using melting curves at $T < T_f$, *i.e.*, using the low-temperature DSC peaks. Melting curves of $n$-decane at $T$ < 243 K could be used to calculate the PSD for silicas with the GT equation $R = k_{GT}/(T_{0,m} - T_m)$ for cylindrical pore radius at $k_{GT}$ = 64.6 K nm, where $T_{0,m}$ and $T_m$ were the melting temperatures for pure bulk

crystallites and confined in pores of radius *R*, respectively. For the freezing point depression of *n*-decane confined in pores (voids), these calculations resulted in the PSD similar to the NLDFT PSD. For water as a probe, there are certain differences between nitrogen adsorption PSD and DSC PSD for different silicas (Figure S35) that is due to several factors: (i) water can be layer-by-layer frozen in mesopores and macropores; (ii) ice (having a larger volume than liquid water) can affect the PSD; (iii) model errors caused by the difference between model pore shape (e.g., cylindrical) and the real one (e.g., voids between NPNP). However, qualitative changes in the DSC PSD and adsorption PSD are similar following the MCA compaction of A-300 (Figure S35a). The DSC thermoporometry, as well as thermogravimetry porometry, could be used for the characterization of the behavior of adsorbates at low and high temperatures that gives additional information on the confined space effects important on practical applications of the materials under different conditions. Note that the confined space effects can be observed not only for unfrozen liquids but also for frozen ones using the TSDC method [33].

## S8. TSDC relaxometry

Thermally stimulated (de)polarization current (TSDC) method could be used to analyze the confined space effects for frozen polar liquids (e.g., water → ice) bound to various disperse or porous solids even at low their content, *i.e.*, diluted aqueous suspensions could be studied due to high-sensitivity of the TSDC method measuring low depolarization currents at $10^{-15}$–$10^{-7}$ A [33]. Tablets (diameter 30 mm, thickness ~1 mm) with frozen studied materials differently hydrated (hydration $h$ = 0.03–99 gram of water per gram of dry material) could be polarized by the electrostatic field at the intensity $F_p$ = 0.1–0.5 MV/m at 260–265 K then cooled to 80–90 K with the field still applied and heated without the field to 265–270 K at a various heating rate (e.g., 0.05 K/s). The current ($I$) evolving due to sample depolarization could be recorded by an electrometer over the $10^{-15}$–$10^{-7}$ A range. Relative mean errors for measured TSDC are relatively small $\delta_I$ = ±5%, $\delta_T$ = ±2 K for temperature, $\delta_\beta$ = ±5% for the temperature change rate [33].

The temperature range on the TSDC measurements is much broader (80–270 K) than that on the $^1$H NMR measurements (180–290 K) because the dipolar relaxation (TSDC) observed at $T$ < 200–220 K is linked to the polar bond rotations or molecular fragment relaxation (observed in confined ice), which cannot be registered in the $^1$H NMR spectra (of static samples with a narrow bandwidth of 20 kHz [8,33]) of mobile low-molecular molecules (water, benzene, acetone, etc.) that appears only at higher temperatures. However, the temperature ranges of the throughout conductivity (DC relaxation in TSDC) and the molecular mobility ($^1$H NMR) are relatively close, but not the same because the DC relaxation requires the throughout percolation of ions in the samples from one electrode to another, but the molecular mobility reflected in the $^1$H NMR spectra can be due to local (cluster) mobility of bound or bulk water (from individual molecules, clusters, nanodomains to bulk water with elevated temperature). Therefore, the DC relaxation in TSDC is observed at slightly higher temperatures than the $^1$H NMR spectra of unfrozen clusters of confined water.

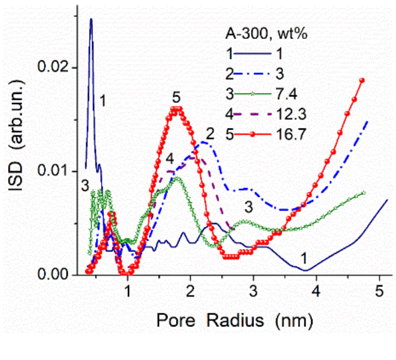


**Figure S36**. Size distributions of water clusters located in voids between silica NPNP in aggregates located in frozen aqueous suspensions of A-300 (TSDC/GT method) at different concentrations

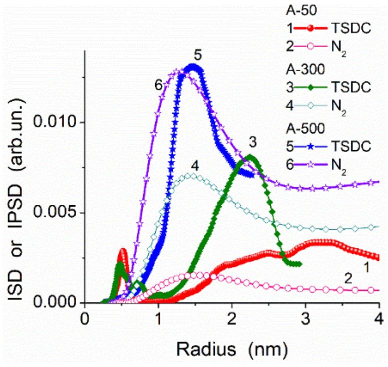


**Figure S37**. Size distributions of pores (nitrogen adsorption/desorption treated with SCV/SCR method) and water clusters located in voids between silica NPNP in aggregates located in frozen aqueous suspensions of A-50, A-300, and A-500 (TSDC/GT method) at 7 wt %.

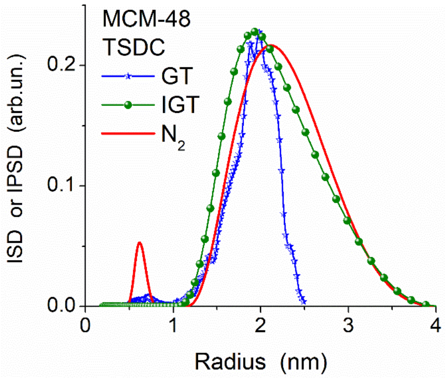


**Figure S38.** Size distributions of pores (nitrogen adsorption/desorption treated with a model of cylindrical pores) and water clusters located in pores of MCM-48 located in frozen aqueous suspension (TSDC/GT and integral GT methods) at 16.7 wt %.

Similar to the $^{1}H$ NMR cryoporometry results for the A-300 suspensions (Figure S33), the water cluster distributions based on the TSDC data (Figure S36) demonstrate nonmonotonic changes in the nanostructured clusters ($R$ < 1 nm) and mesostructured clusters and domains ($R$ = 1–5 nm) with increasing concentration of silica in the aqueous suspensions. However, for different nanosilicas, such as A-50 ($S_{BET}$ = 52 $m^2/g$), A-300 (294 $m^2/g$), and A-500 (492 $m^2/g$), there are practically monotonic changes in the cluster sizes and their contributions (Figure S37) because the smaller the NPNP size, the stronger the aggregation of NPNP affecting the TSDC spectra of confined water (ice). Note that integral GT equation based on the TSDC relaxation vs. temperature (similar to integral GT Equation (26) for NMR cryoporometry, but with a certain dependence of $k_{GT}$ on $T$ [8,33]) gives the $f(R)$ function close to the $N_2$ PSD than that calculated using non-integral equation (Figure S38). Thus, the TSDC relaxation data could be used for additional characterization of confined liquids at $T < T_f$ when only dipolar relaxation of bonds and certain atomic fragments is observed in ice (i.e., with no molecular mobility for confined unfrozen liquids observed in the NMR cryoporometry or DSC thermoporometry). TSDC relaxometry, characterized by certain advantages and disadvantages, as well other described methods, gives additional information allowing one a deeper insight into the interfacial phenomena and complete characterization of the materials in different conditions.

The present study deals with analyses of a set of methods used to investigate the morphological and textural characteristics of nano/meso/macroporous materials being in various dispersion media, as well the interfacial phenomena basic for various approaches in the characterization of materials. There are several ways in these studies: (i) adsorption–desorption of low-molecular-weight probe compounds ($N_2$, Ar, etc.); (ii) adsorption or confinement of low- or high-molecular-weight compounds in pores (voids) of solid particles being in liquid media; (iii) small-angle X-ray scattering (SAXS); (iv) quantitative analysis of images recorded using various microscopic methods; (v) thermoporometry based on differential scanning calorimetry (DSC) with decreasing-increasing temperature using melting thermograms; (vi) cryoporometry based on low-temperature $^{1}H$ NMR spectroscopy giving the dependence of the signal intensity of a mobile phase vs. temperature; (vii) relaxometry based on NMR spectroscopy with transverse relaxation time vs. temperature; and (viii) relaxometry based on thermally stimulated depolarization current (TSDC) measurements. Each method could be characterized by certain systematic errors. However, the use of several aforementioned methods in parallel can allow one to elucidate the reasons and level of systematic errors that are important for the correct characterization of the materials studied. Thus, the larger the number of methods used in parallel, the more comprehensive the morphological and textural characterization of the adsorbents.

One of the main effects of liquid dispersion media onto FMO is changes in the organization of the secondary particles, i.e., NPNP aggregates and agglomerates of aggregates, that non-monotonically depends on the concentration of FMO and typically, the secondary particles become less compacted. The opposite effect is due to any mechanical loading on dry or wetted FMO powders, which become more compacted.

**S9. Confocal laser scanning microscopy**

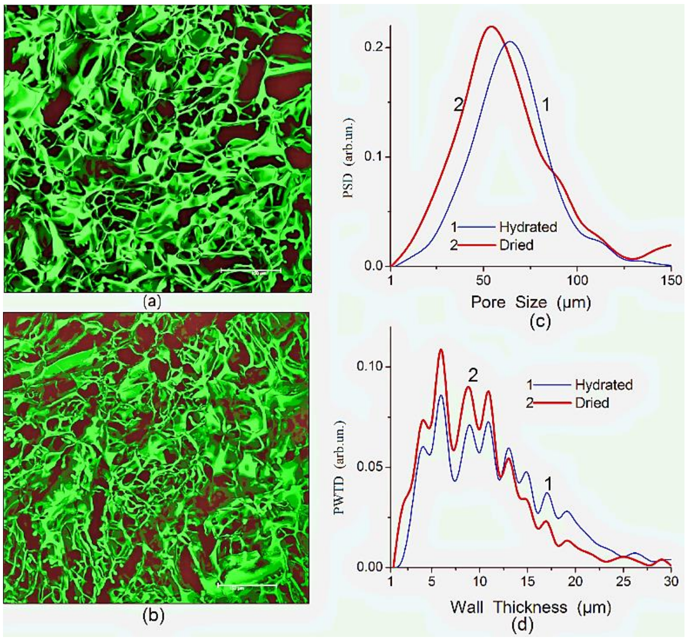


**Figure S39**. Confocal laser scanning microscopy images of macroporous poly(2-hydroxyethyl methacrylate-allyl glycidyl ether) cryogel in (a) hydrated and (b) dried states with the pore (c) size (PSD) and (d) wall thickness (PWTD) distributions [25].

**S10. Models and quantum chemical methods**

In the models used, dozens of polar ($H_2O$, $NH_3$, $CO_2$) and nonpolar ($C_6H_6$, $N_2$, $CH_4$ or fragments of polydimethylsiloxane, PDMS, poly(vinyl alcohol), PVA, poly(ethylene glycol), PEG) molecules and some of their mixtures were adsorbed onto hydrophilic silica (models with 8–88 tetrahedra in DFT and hundreds of tetrahedra in PM7) and hydrophobic silica clusters with attached dimethylsilyl or trimethylsilyl groups. Quantum chemical calculations were carried out using the DFT method with a hybrid functional ωB97X-D, and the cc-pVDZ or aug-cc-pVTZ basis sets using the Gaussian 09 (D.01) [34] and GAMESS 2020.R2 [35] program suits. The solvation effects were analyzed using the SMD method [36]. The gauge-independent atomic orbital (GIAO) method [34] was used to calculate the NMR spectra of certain systems. Larger structures (up to 18,000 atoms) were calculated using semiempirical PM7 method (MOPAC 2016) [37,38]. Visualization of the calculation results was carried out using several programs described in detail elsewhere [39–41].

The used models of nanosilica particles (Figure S40) and pores in silica gels (Figure S41) reflect the main textural features of these adsorbents. Activated carbons (AC) as well silicas are the most important adsorbents used in various industry, medical, and scientific applications. AC and silicas are characterized by very different textures and surface nature. These differences should be reflected in the particulate models used (Figures S40–S42, Tables S2 and S3). One of the important factors in the analyses of the adsorption phenomena is that the results on, e.g., the textural characterization depends on the characteristics not only of adsorbents but also adsorbates. For example, the PSD of a set of AC calculated using the nitrogen and benzene as probes differ (Figure S9) because nitrogen and benzene molecules are of different sizes and nature. Thus, any adsorbate using as an adsorption probe can affect the adsorption results that lead to a certain ambiguity in the adsorbent characterization.

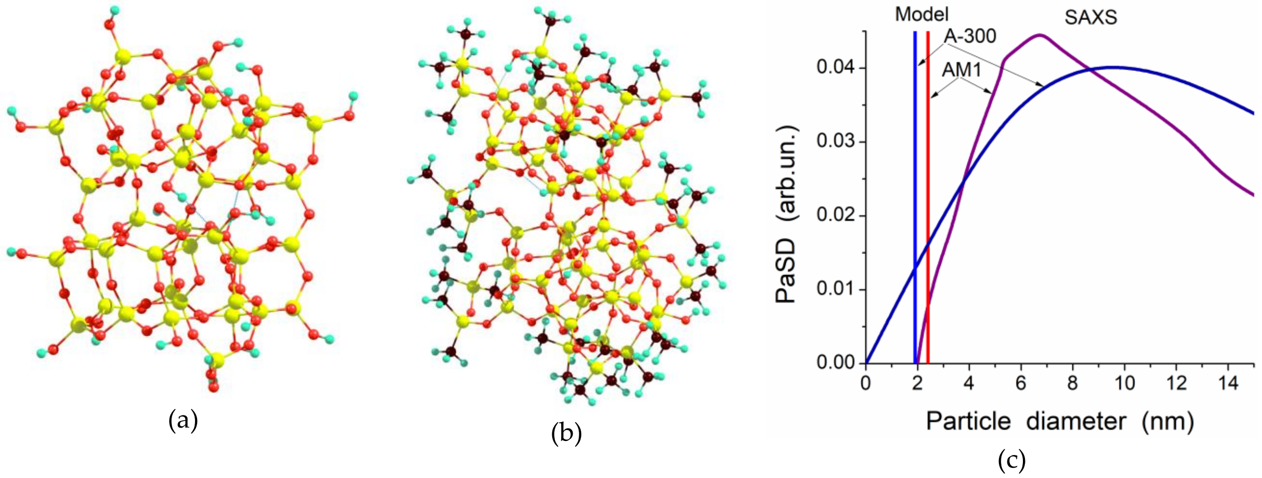


**Figure S40.** Models of hydrophilic (a) and hydrophobic (b) silica nanoparticles: (a) 44 tetrahedrons ($SiO_{4/2}$) with 24OH groups, (b) 44 ($SiO_{4/2}$)+19(=$Si(CH_3)_2$)+5OH modeling A-300 hydrophobized by dimethyldichlorosilane, hydrolyzed and lateral-crosslinked (hydrophobic nanosilica AM1); geometry was optimized using DFT ωB97X-D/cc-pVDZ; (c) particle size distributions (PaSD) of A-300 and AM1 based on SAXS data.

Nonpolar nitrogen molecules are polarized and weakly charged due to interactions with any adsorbent (Tables S2 and S3). However, the confined space effects are absent for silica NPNP (Figure S41a). Therefore, the calculated interaction energy is relatively small since it corresponds to the second peak of the adsorption energy distributions (ED) $f(E)$ upon the interaction of molecules only with one surface (Figures S6–S8 and S11) characterized by several peaks. The first peak at the $E$ values close to the heat of vaporization of nitrogen molecules (5.56 kJ/mol) corresponds to the adsorbed molecules (AM), which do not sense the pore walls, i.e., they adsorbed in broad pores far from the pore walls. The second $f(E)$ peak corresponds to AM sensing only one pore wall in broad mesopores. In narrow pores, AM can weakly and strongly sense two walls that result in the third $f(E)$ peak. In nanopores, AM strongly sense two walls that correspond to the fourth $f(E)$ peak.

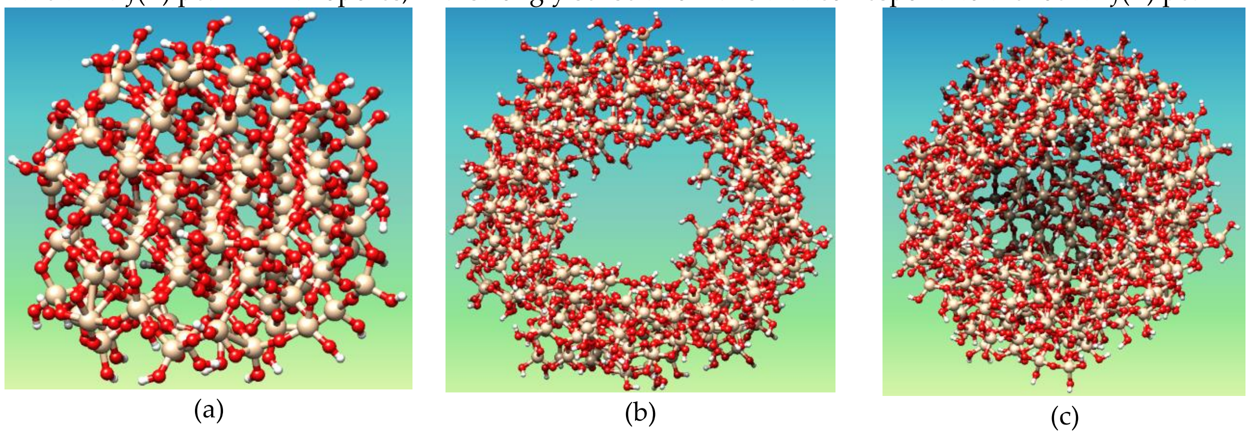


**Figure S41.** Models of nonporous (a) and porous (b,c) particles of silica at (a) particle diameter $d$ = 2.2 nm, (b,c) $d$ = 3.6 nm, pore diameter = 1.2 nm, (c) one-side closed pore 4 nm deep.

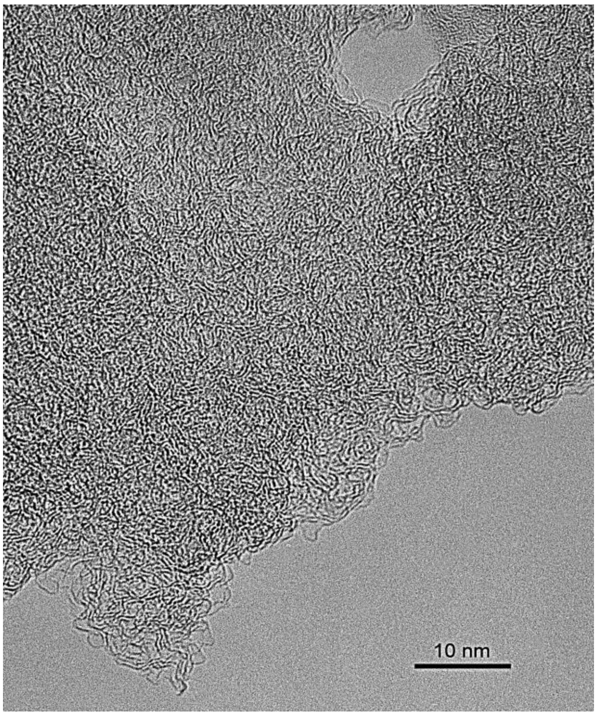


(a)

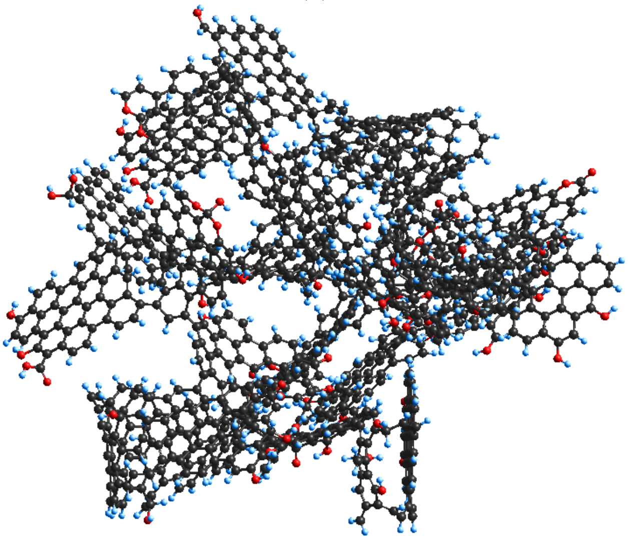

(b)

**Figure S42.** (a) HRTEM image of AC with 50% burn-off, (b) AC model (~5 nm in size, 1589 atoms) used in adsorption studies (PM7 geometry).

**Table S2.** Adsorption energy ($\Delta E_{1,BSSE}$) and charge transfer ($\Delta q_1$) per a molecule for various probes adsorbed onto silica ($\omega$B97X-D/cc-pVDZ).

| Adsorbate | $-\Delta E_1$ (kJ/mol) | $\Delta q_1$ (a.u.) |
|---|---|---|
| $11C_6H_6$ 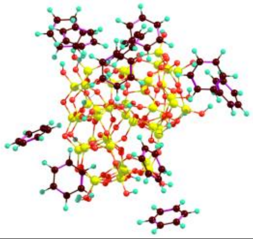 | 32.8 | -0.033 |
| 40 $N_2$ | 6.9 | 0.019 |

| | | |
|---|---|---|
| 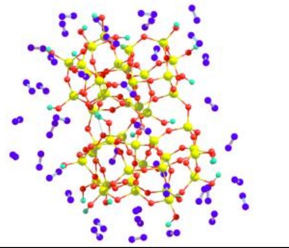 | | |
| $37CO_2$ 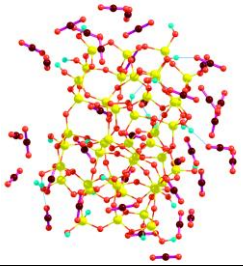 | 14.5 | 0.013 |
| $34NH_3$ $NH_4^+$ 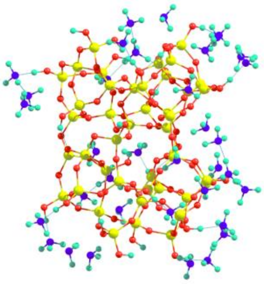 | 62.8 | 0.068 |
| $41H_2O$ $H_3O^+$ 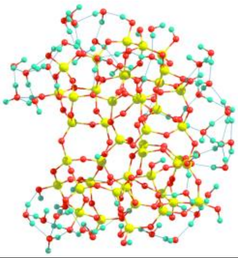 | 74.5 | 0.067 |
| $17H_2O$ $32NH_3$ $2NH_4^+$ 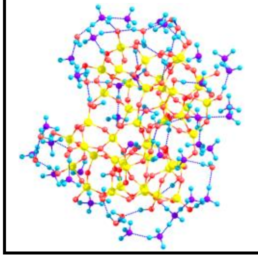 | 63.0 | 0.068 |

**Table S3.** Adsorption energy ($\Delta E_1$) per molecule of various probes adsorbed onto silica and AC (PM7).

| Adsorbent | Adsorb-ate | $-\Delta E_1$ (kJ/mol) |
|---|---|---|
| Silica 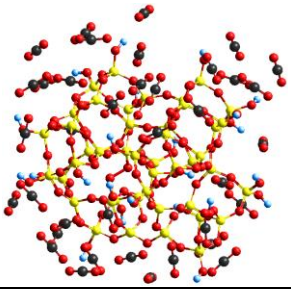 | $37CO_2$ | 13.5 |

| | | |
|---|---|---|
| Silica 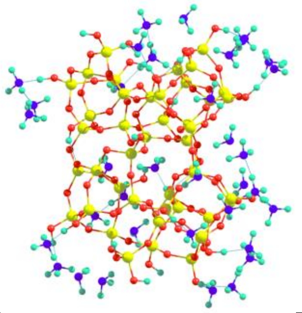 | $35NH_3$ | 27.3 |
| AC 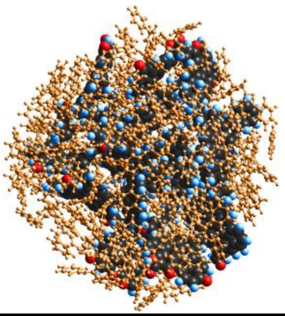 | $227C_6H_6$ | 4.0 |
| AC 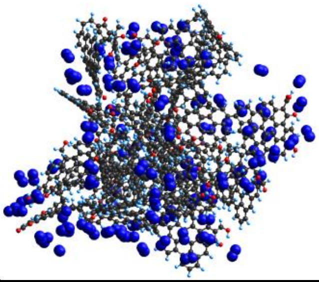 | 99 $N_2$ | 4.6 |
| AC 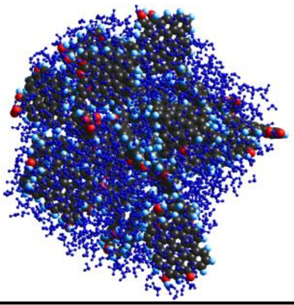 | $1397H_2O$ | 6.3 |

Besides the confined space effects in pores of different sizes and the effects caused by the surface structure and composition, there is an effect of orientation of adsorbed molecules (Tables S2 and S3). The latter depends not only on the nature of a solid surface but also on lateral interactions. Therefore, for silicas and AC, the surface area occupied by $N_2$ molecule $\sigma_{eff}$ = (0.85÷0.90)×$\sigma_0$. Thus, the value of $S_{BET}$ estimated using $\sigma_0$ = 0.162 nm$^2$ is always overestimated.

An increase in the number of adsorbed molecules of water (Figure S43a) or other molecules (Figure S43b) typically leads to a reduction of the interaction energy and charge transfer per molecule because only molecules from the first adsorption layer strongly sense the surface in contrast to distant molecules. For polar molecules interacting with hydroxylated surfaces, it is possible proton transfer, which strongly changes the characteristics of the adsorption layer. Note that for alumina/silica cluster (Si/Al = 38/6) with $40H_2O$+$2H_3O^+$, $\Delta E_{1,w}$ = -77.3 kJ/mol and $\Delta q_{1,w}$ = 0.086 and these values are greater than that for similar pure silica cluster with bound water due to increased polarization effects.

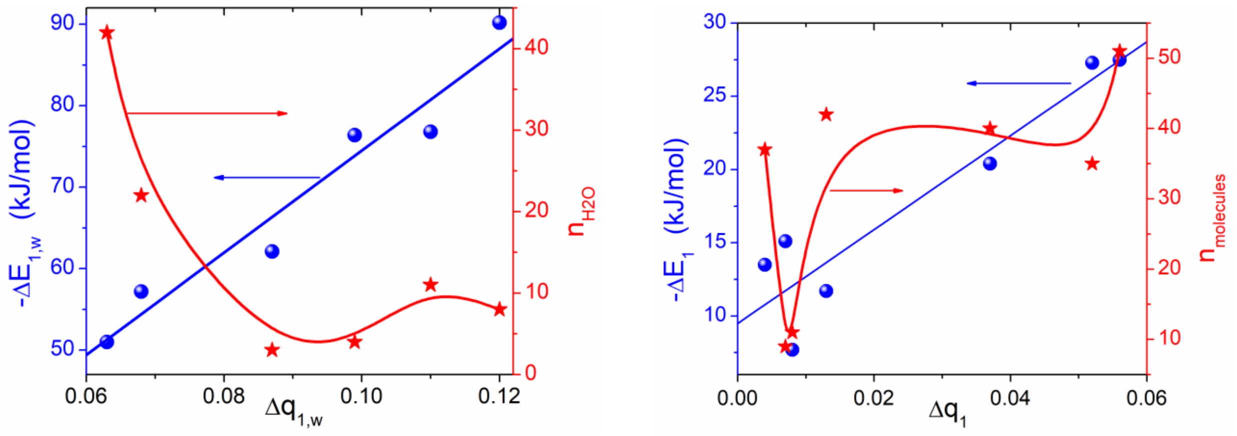


(a) (b)

**Figure S43.** (a) DFT (ωB97X-D/cc-pVDZ) results for bound water and (b) PM7 results for interaction energy vs. charge transfer upon adsorption of $N_2$, $NH_3$, $CO_2$, $H_2O$, $NH_3$+$H_2O$, and $C_6H_6$ (various numbers of molecules) onto hydrophilic and hydrophobic silica nanoparticles (per a molecule).

These phenomena are clear in the $^1$H NMR spectra of bound adsorbates (Figure S44), showing strong down-field shifts (peaks at 9–14 ppm) because of reducing proton shielding. This effect is observed in the NMR spectra of both liquid and solid acids [8]. The confined space effects appear in the $^1$H NMR spectra of water bound in pores or onto NPNP (Figure S45). The former leads to the downfield shift compared to the latter. However, surface modification, e.g., hydrophobization of a silica surface or compaction of FMO, changes the confined space effects for both polar and nonpolar adsorbates. This is especially characteristic of water, which tends to form clusters at both polar or nonpolar surfaces. However, the hydrophobization strongly reduces the interaction energy even in nanopores.

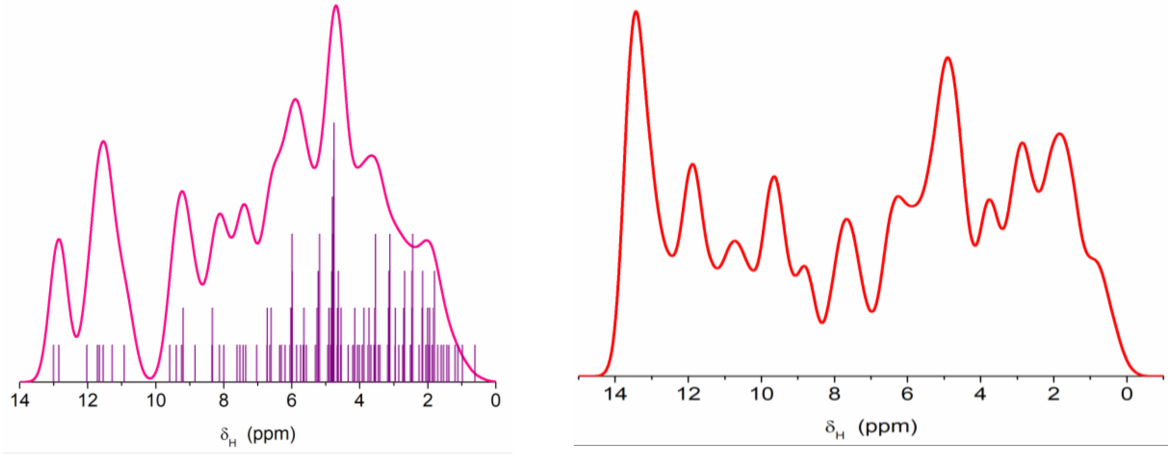


(a) (b)

**Figure S44.** $^1$H NMR spectra of (a) a cluster with $H^+$ transfer 44($SiO_{4/2}$) with 23(OH)($O^-$) + 39$H_2O$+$OH_3^+$ $\Delta E_{BSSE,1}$ = -74.5 kJ/mol, $\Delta q_t$ = 2.81, $\Delta q_1$ = 0.067. Structures around $H_3^+O$, strong H-bonds, H-bonds, weak H-bonds, and free OH; (b) effects of $H^+$ transfer: 44($SiO_{4/2}$) with 22(OH)@2($O^-$) + 17$H_2O$+32$NH_3$ + 2$NH_4^+$ $\Delta E_{BSSE,1}$ = -60 kJ/mol (both ads) and -67.8 kJ/mol (ads1+ads2), $\Delta q_t$ = 3.46, $\Delta q_1$ = 0.068; (b) structures around $NH_4^+$, strong H-bonds, H-bonds, weak H-bonds, and free OH (GIAO/ωB97X-D/cc-pVDZ).

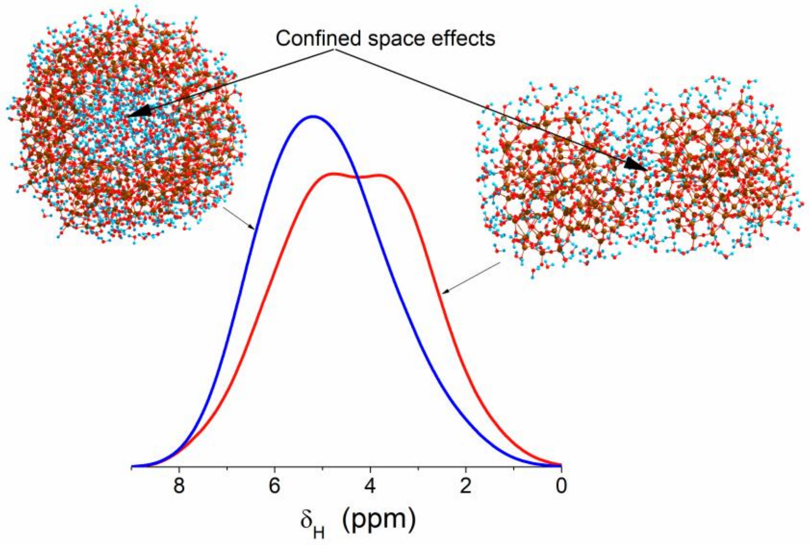


**Figure S45.** Theoretical $^{1}$H NMR spectra of water bound to porous and nonporous nanoparticles (PM7).

The surface structure and texture (i.e., confined space effects), as well conditions (temperature, pressure, time of adsorption, co-adsorbates) (Figures S40–S47, Tables S2 and S3), strongly affect the dynamic behavior of adsorbates (Figures S48–S50). Clearly, physical (non-activated) adsorption decreases with increasing temperature (Figure S48). However, an increase in pressure enhances the adsorption due to the faster return of molecules from the gaseous phase into the adsorption layer. This effect is well seen on comparison of the dynamic behavior of water molecules bound to silica located in a periodic box (the molecules can return to the surface) and upon desorption in open space (with no return of the molecules to the surface) (Figure S49a). If the molecules are adsorbed in pores, their evaporation is restricted (Figure S49b,c), especially in the semi-closed pore (Figure S49d).

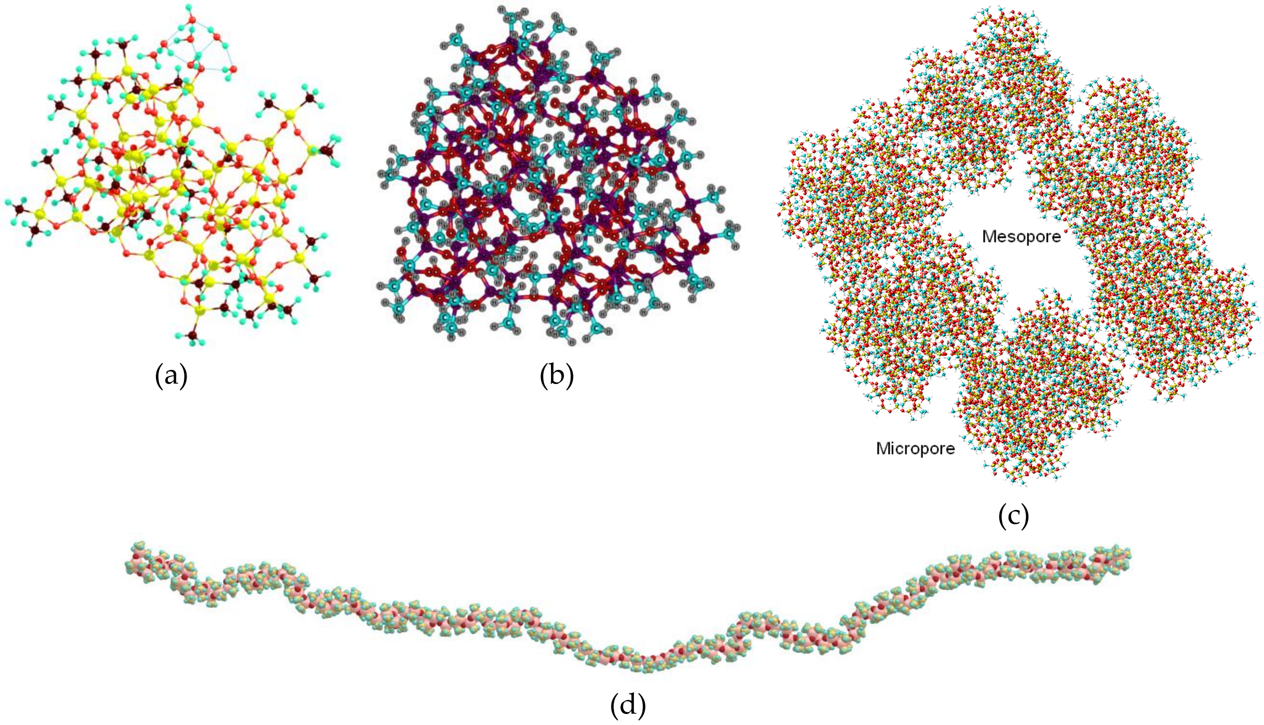


**Figure S46.** (a) Clustered water adsorption onto hydrophobic AM1, $\Delta E_{1,w}$ = −29.0 kJ/mol (with BSSE correction, ωB97X-D/cc-pVDZ), −15.1 kJ/mol (PM7), $\Delta q_{1,w}$ = 0.033 (ωB97X-D/cc-pVDZ), 0.007 (PM7). (b) Dried (dehydrated) PMS particle (PM7 calculation) with 119 tetrahedrons and residual 9 OH groups, (c) multi-nanoparticle PMS structure (porous aggregate), and (d) PDMS molecule with 150 units (PM7 calculations).

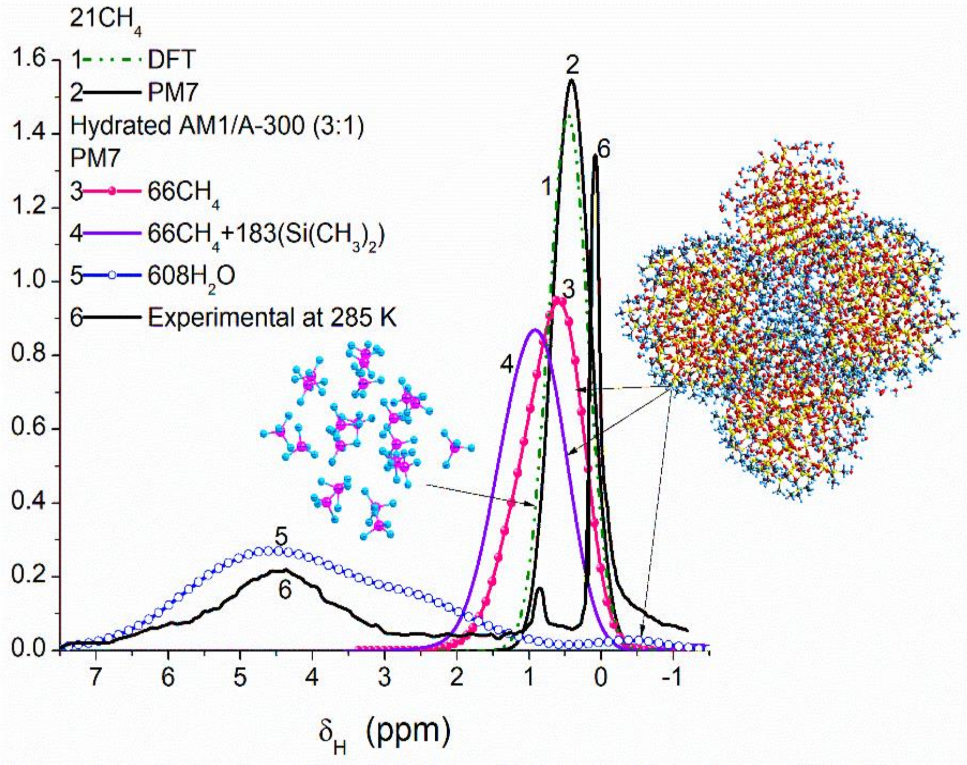


**Figure S47.** Theoretical ($21CH_4$—curves 1 (gauge-independent atomic orbital (GIAO)/ωB97X–D/cc–pVDZ) and 2 (PM7), a model AM1/A-300 (3:1, with 3 AM1 particles (each with 61 groups of $Si(CH_3)_2$ and 123 $SiO_{4/2}$ tetrahedrons) and one silica particles (123 $SiO_{4/2}$ tetrahedrons) and bound $608H_2O$ and $66CH_4$) shown in the insert was calculated using PM7 method—curves 3–5) and experimental (curve 6, AM1/A-300 (3:1) at $h$ = 0.1 g/g and 285 K) $^1$H NMR spectra.

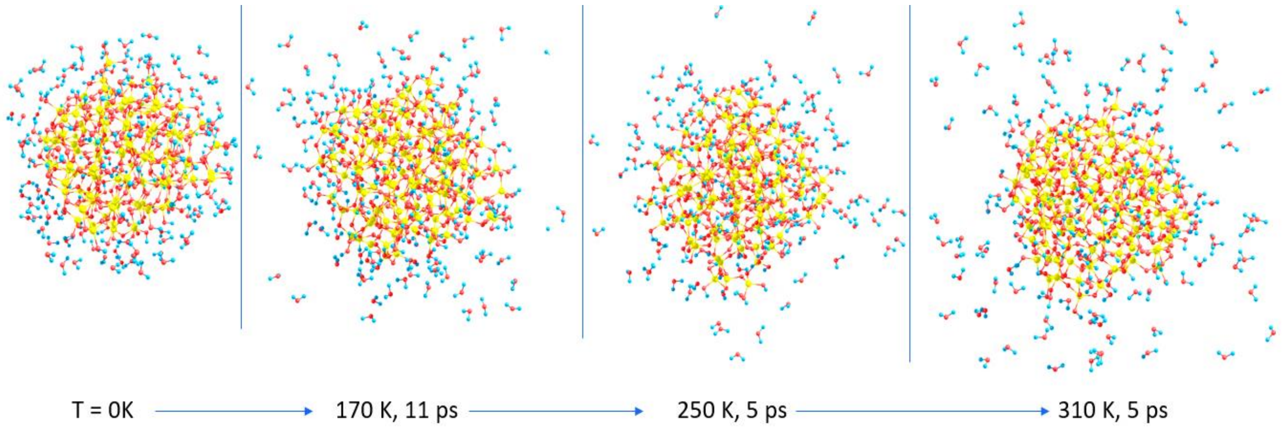


**Figure S48**. MD model of water bound to silica vs. temperature: MM+ force-field calculations with periodic boundary conditions, water shell with 107 $H_2O$ around silica nanoparticles heated at various temperatures (with no confined space effects). This behavior of water molecules results in weak adsorption onto non-aggregated nonporous silica nanoparticles. Nanoparticle aggregation (i.e., the appearance of the textural porosity) leads to a certain increase in the adsorption of water onto fumed silica, but it is much smaller than that adsorbed onto porous micro/macroparticles (e.g., silica gel).

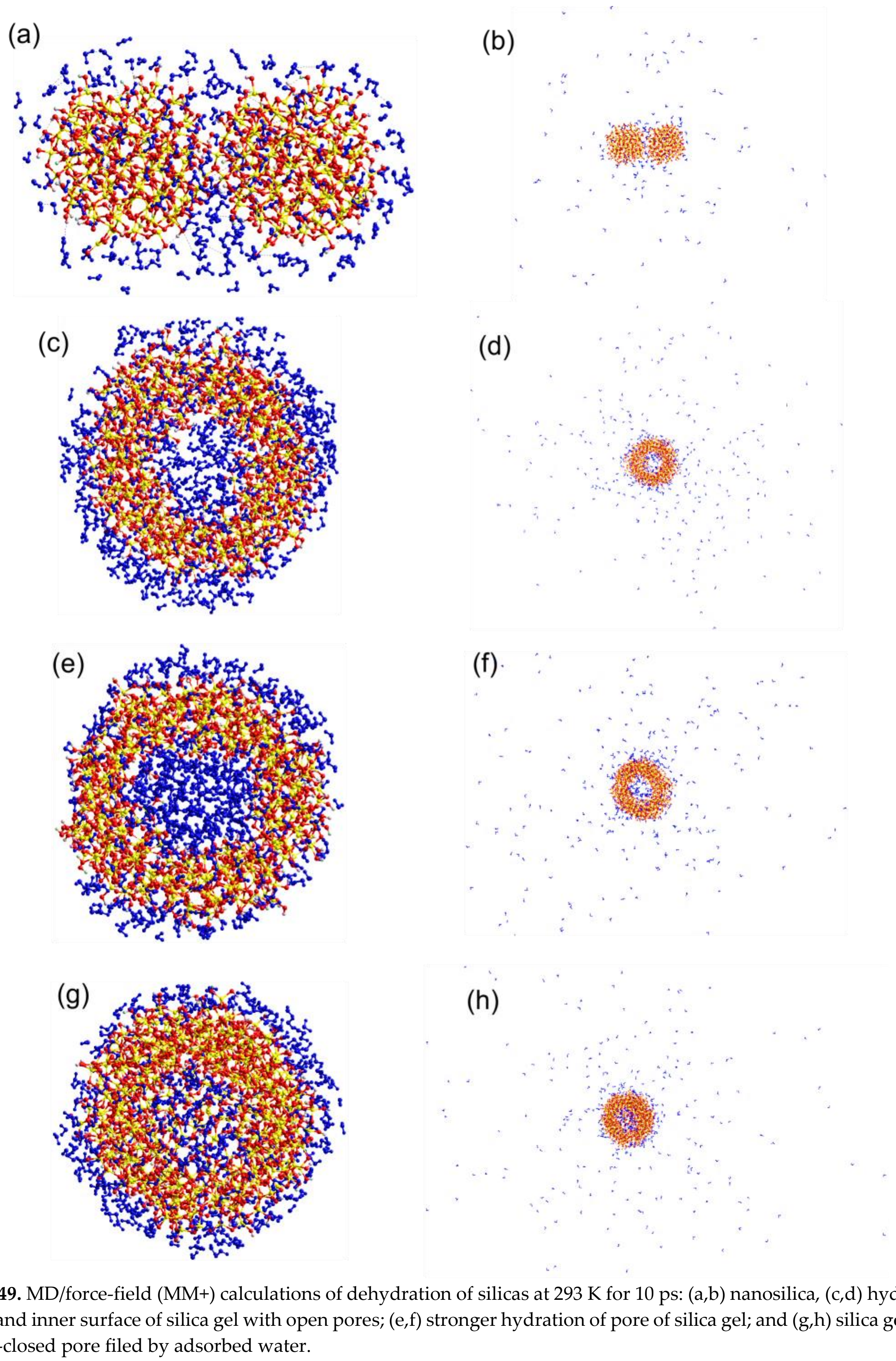


**Figure S49.** MD/force-field (MM+) calculations of dehydration of silicas at 293 K for 10 ps: (a,b) nanosilica, (c,d) hydration of outer and inner surface of silica gel with open pores; (e,f) stronger hydration of pore of silica gel; and (g,h) silica gel with one-side-closed pore filed by adsorbed water.

Desorption of water molecules decreases if they co-adsorbed with both polar or nonpolar polymers in pores (Figure S50) due to enhanced confined space effects. Polar polymers enhance the adsorption energy that also reduced the evaporation of water molecules from pores.

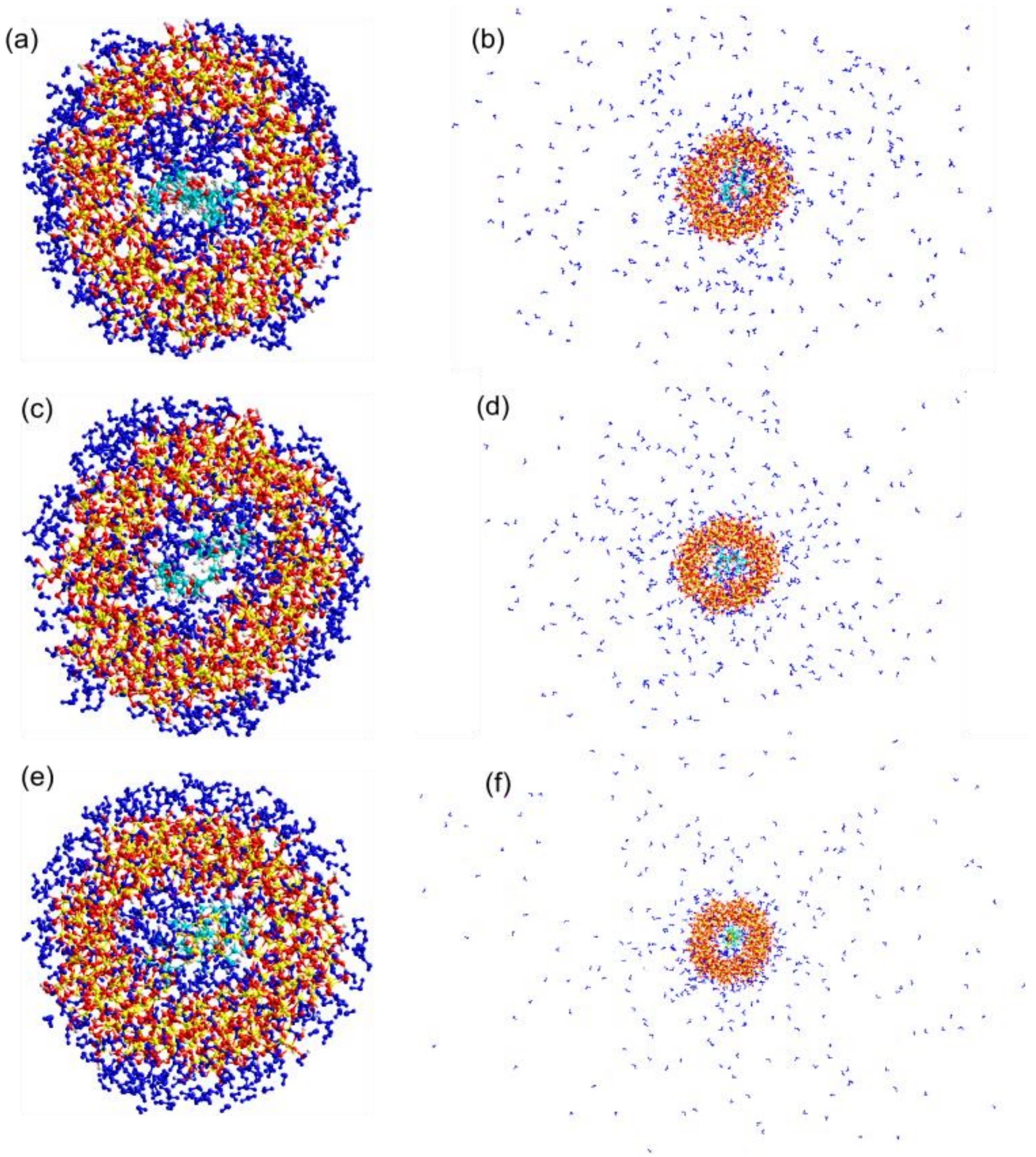


**Figure S50**. Effects of co-adsorbates: MD/force-field (MM+) calculations of dehydration of silica gel particle at 293 K for 10 ps with polymers located in pore: (a, b) PVA (two fragments), (c, d) PEG (two fragments); (e, f) PDMS (one fragment).

**Conclusion remarks**

Note that firm software distributed with related equipment (e.g., adsorption, SAXS, XRD, NMR, TG, FTIR, DSC, QESL, etc.) needs additional hard efforts of the developers. However, there are significant barriers to the fast development of the applied software by the scientific community because firm software is typically proprietary with closed codes, and it is not cheap. As a whole, too high-level of commercialization of science, publishing, software development, etc., strongly decelerates the whole progress (e.g., comp. efficiency of free various Linux OS and closed any Microsoft Windows having numerous problems in each version, upgrade, and update). Therefore, all results of hard computations (quantum chemistry) shown here were obtained using free Linux OS (Clear Linux OS, Fedora, Arch Linux) as well as some free programs (e.g., GAMESS, CONTIN, etc.). The author has developed many programs (and models) (e.g., self-consistent regularization (SCR) procedures for adsorption (DFT, MND, PaSD) and SAXS (PaSD, PSD), different versions of cryoporometry, relaxometry, thermoporometry, programs for treatments of the data of QELS, XRD, NMR, FTIR, TPD MS, TG, DSC, CLSM, rheometry, and other methods), but similar programs (e.g., SCR) are absent in the firm software. Based on wide investigations of dispersing and porous materials over four decades, it is possible to conclude that the use of several methods in parallel with appropriate computational treatments of the data provides a significantly deeper insight into the problems studied.

**References**


1. Gregg, S.J.; Sing K.S.W. *Adsorption, Surface Area and Porosity*. 2nd ed. Academic Press: London, 1982.
2. Adamson, A.W.; Gast, A.P. *Physical Chemistry of Surface*. 6th edition. Wiley: New York, 1997.
3. Somasundaran, P.; Ed.; *Encyclopedia of Surface and Colloid Science*. Third Edition. CRC Press: Boca Raton, 2015.
4. Gun'ko, V.M.; Turov, V.V.; Zarko, V.I.; Goncharuk, O.V.; Pahklov, E.M.; Skubiszewska-Zięba, J.; Blitz, J.P. Interfacial phenomena at a surface of individual and complex fumed nanooxides. *Adv. Colloid Interface Sci.* **2016**, *235,* 108.
5. Iler, R.K. *The Chemistry of Silica*. Wiley: Chichester, 1979.
6. Legrand, A.P.; Ed.; *The Surface Properties of Silicas*. Wiley: New York, 1998.
7. Bergna, H.E.; Roberts W.O. *Colloidal Silica: Fundamentals and Applications*. CRC Press: Boca Raton, 2006.
8. Gun'ko, V.M.; Turov V.V. *Nuclear Magnetic Resonance Studies of Interfacial Phenomena*. CRC Press: Boca Raton, 2013.
9. Gun'ko, V.M. Composite materials: textural characteristics. *Appl. Surf. Sci.* **2014,** *307***,** 444.
10. Gun'ko, V.M.; Meikle, S.T.; Kozynchenko, O.P.; Tennison, S.R.; Ehrburger-Dolle, F.; Morfin, I.; Mikhalovsky, S.V. Comparative characterization of carbon and polymer adsorbents by SAXS and nitrogen adsorption methods. *J. Phys. Chem. C* **2011**, *115***,** 10727.
11. Do, D.D.; Nguyen, C.; Do, H.D. Characterization of micro-mesoporous carbon media. *Colloids Surf. A: Physicochem. Eng. Aspects* **2001**, *187–188***,** 51.
12. Platzer, B.; Maurer, G. Application of a generalized Bender equation of state to the description of vapour-liquid in binary systems. *Fluid Phase Equilibr.* **1993**, *84***,** 79.
13. Muniz, W.B.; Ramos, F.M.; de Campos Velho, H.F. Entropy- and Tikhonov-based regularization techniques applied to the backwards heat equation. *Comput. Mathem. Applic*. **2000**, *40,* 1071.
14. Provencher, S.W. A constrained regularization method for inverting data represented by linear algebraic or integral equations. *Comp. Phys. Comm.* **1982**, *27***,** 213.
15. Gun'ko, V.M.; Turov, V.V.; Turov, A.V.; Zarko, V.I.; Gerda, V.I.; Yanishpolskii, V.V.; Berezovska, I.S.; Tertykh, V.A. Behaviour of pure water and water mixture with benzene or chloroform adsorbed onto ordered mesoporous silicas. *Central European Journal of Chemistry* **2007**, *5,* 420.
16. Gun'ko, V.M.; Turov, V.V.; Pakhlov, E.M.; Krupska, T.V.; Charmas, B. Effect of water content on the characteristics of hydro-compacted nanosilica. *Appl. Surf. Sci*. **2018**, *459***,** 171.
17. Fairén-Jiménez, D.; Carrasco-Marín, F.; Djurado, D.; Bley, F.; Ehrburger-Dolle, F.; Moreno-Castilla, C. Surface area and microporosity of carbon aerogels from gas adsorption and small- and wide-angle X-ray scattering measurements. *J. Phys. Chem. B* **2006**, *110***,** 8681.
18. Brumberger, H.; Ed.; *Small Angle X-ray Scattering*. Gordon & Breach: New York, Syracuse, 1965.
19. Sternik, D.; Galaburda, M.; Bogatyrov, V.M.; Gun'ko, V.M. Influence of the synthesis method on the structural characteristics of novel hybrid adsorbents based on bentonite. *Colloids Interfaces* **2019**, *3*, 18.
20. Ares, A.E.; Ed.; *X-ray Scattering*. InTech: Croatia, 2017. DOI: 10.5772/65049
21. Gun'ko, V.M. Various methods to describe the morphological and textural characteristics of various materials. *Chemistry, Physics and Technology of Surface* **2018**, *9,* 317.
22. Dieudonné, Ph.; Hafidi, A.A.; Delord, P.; Phalippou, J. Transformation of nanostructure of silica gels during drying. *J. Non-Crystal. Solid*. **2000**, *262***,** 155.
23. *ImageJ*. 2021. https://imagej.nih.gov/ij/, https://imagej.nih.gov/ij/plugins/granulometry.html.
24. *Fiji*. 2021. https://fiji.sc/, https://imagej.net/Local_Thickness.
25. Gun'ko, V.M.; Savina, I.N.; Mikhalovsky, S.V. Cryogels: Morphological, structural and adsorption characterization. *Adv. Colloid Interface Sci*. **2013**, *187–188***,** 1.
26. Gun'ko, V.M.; Zarko, V.I.; Leboda, R.; Chibowski, E. Aqueous suspensions of fumed oxides: particle size distribution and zeta potential. *Adv. Colloid Interface Sci*. **2001**, *91***,** 1.
27. Gun'ko, V.M.; Klyueva, A.V.; Levchuk, Yu.N.; Leboda, R. Photon correlation spectroscopy investigations of proteins. *Adv. Colloid Interface Sci*. **2003**, *105***,** 201.
28. Hunter, R.J. *Introduction to Modern Colloid Science.* Oxford University Press: London, 1993.
29. Mitchell, J.; Webber, J.B.W.; Strange, J.H. Nuclear magnetic resonance cryoporometry. *Phys. Rep*. **2008**, *461***,** 1.
30. Petrov, O.V.; Furó, I. NMR cryoporometry: Principles, applications and potential. *Progr. NMR Spectroscopy* **2009**, *54*, 97.
31. Hay, J.N.; Laity, P.R. Observations of water migration during thermoporometry studies of cellulose films. *Polymer* **2000**, *41,* 6171.

32. Landry, M.R. Thermoporometry by differential scanning calorimetry: experimental considerations and applications. *Thermochim Acta* **2005**, *433,* 27.
33. Gun'ko, V.M.; Zarko, V.I.; Goncharuk, E.V.; Andriyko, L.S.; Turov, V.V.; Nychiporuk, Y.M.; Leboda, R.; Skubiszewska-Zięba, J.; Gabchak, A.L.; Osovskii, V.D.; Ptushinskii, Y.G.; Yurchenko, G.R.; Mishchuk, O.A.; Gorbik, P.P.; Pissis, P.; Blitz J.P. TSDC spectroscopy of relaxational and interfacial phenomena. *Adv. Colloid Interface Sci*. **2007**, *131*, 1.
34. Frisch M.J.; Trucks G.W.; Schlegel H.B.; Scuseria G.E.; Robb M.A.; Cheeseman J.R.; Scalmani G.; Barone V.; Mennucci B.; Petersson G.A.; Nakatsuji H.; Caricato M.; Li X.; Hratchian H.P.; Izmaylov A.F.; Bloino J.; Zheng G.; Sonnenberg J.L.; Hada M.; Ehara M.; Toyota K.; Fukuda R.; Hasegawa J.; Ishida M.; Nakajima T.; Honda Y.; Kitao O.; Nakai H.; Vreven T.; Montgomery J.A.; Peralta J.E.; Ogliaro F.; Bearpark M.; Heyd J.J.; Brothers E.; Kudin K.N.; Staroverov V.N.; Kobayashi R.; Normand J.; Raghavachari K.; Rendell A.; Burant J.C.; Iyengar S.S.; Tomasi J.; Cossi M.; Rega N.; Millam J.M.; Klene M.; Knox J.E.; Cross J.B.; Bakken V.; Adamo C.; Jaramillo J.; Gomperts R.; Stratmann R.E.; Yazyev O.; Austin A.J.; Cammi R.; Pomelli C.; Ochterski J.W.; Martin R.L.; Morokuma K.; Zakrzewski V.G.; Voth G.A.; Salvador P.; Dannenberg J.J.; Dapprich S.; Daniels A.D.; Farkas Ö.; Foresman J.B.; Ortiz J.V.; Cioslowski J.; Fox D.J. *Gaussian 09, Revision D.01.* Gaussian, Inc. Wallingford CT, 2013.
35. Barca G.M.J.; Bertoni C.; Carrington L.; Datta D.; De Silva N.; Deustua J.E.; Fedorov D.G.; Gour J.R.; Gunina A.O.; Guidez E.; Harville T.; Irle S.; Ivanic J.; Kowalski K.; Leang S.S.; Li H.; Li W.; Lutz J.J.; Magoulas I.; Mato J.; Mironov V.; Nakata H.; Pham B.Q.; Piecuch P.; Poole D.; Pruitt S.R.; Rendell A.P.; Roskop L.B.; Ruedenberg K. Recent developments in the general atomic and molecular electronic structure system. *J. Chem. Phys.* **2020**, *152*, 154102.
36. Marenich, A.V.; Cramer, C.J.; Truhlar, D.G. Universal solvation model based on solute electron density and on a continuum model of the solvent defined by the bulk dielectric constant and atomic surface tensions. *J. Phys. Chem. B* **2009**, *113*, 6378.
37. Stewart, J.J.P. *MOPAC2016*. Stewart Computational Chemistry, web: HTTP://OpenMOPAC.net. February 21, 2021.
38. Stewart, J.J.P. Optimization of parameters for semiempirical methods VI: more modifications to the NDDO approximations and re-optimization of parameters. *J. Mol. Mod*. **2013**, *19,* 1.
39. Dennington, R.; Keith, T.; Millam, J. GaussView, Version 5.09. Semichem Inc.; Shawnee Mission KS, 2013.
40. Zhurko, G.A.; Zhurko, D.A. *Chemcraft* (version 1.8). 2021, http://www.chemcraftprog.com.
41. Hanwell, M.D.; Curtis, D.E.; Lonie, D.C.; Vandermeersch, T.; Zurek, E.; Hutchison, G.R. Avogadro: an advanced semantic chemical editor, visualization, and analysis platform. *J. Chem. Inf.* **2012**, *4*(17), 1-17.
